# Real-world safety of palbociclib in breast cancer patients in the United States: a new user cohort study

**DOI:** 10.1186/s12885-021-07790-z

**Published:** 2021-01-25

**Authors:** Daniel C. Beachler, Cynthia de Luise, Aziza Jamal-Allial, Ruihua Yin, Devon H. Taylor, Ayako Suzuki, James H. Lewis, James W. Freston, Stephan Lanes

**Affiliations:** 1 HealthCore, Inc., 123 Justison Street, Suite 200, Wilmington, DE 19801 USA; 2 Pfizer Inc., New York, NY USA; 3 Anthem, Inc., Andover, MA USA; 4 Duke University School of Medicine, Durham, NC USA; 5 Georgetown University School of Medicine, Washington, DC USA; 6 University of Connecticut Health Center, Farmington, CT USA

**Keywords:** Palbociclib, Safety, Real-world, Epidemiology, Acute liver injury

## Abstract

**Background:**

There is limited real-world safety information on palbociclib for treatment of advanced stage HR+/HER2- breast cancer.

**Methods:**

We conducted a cohort study of breast cancer patients initiating palbociclib and fulvestrant from February 2015 to September 2017 using the HealthCore Integrated Research Database (HIRD), a longitudinal claims database of commercial health plan members in the United States.

The historical comparator cohort comprised patients initiating fulvestrant monotherapy from January 2011 to January 2015. Propensity score matching and Cox regression were used to estimate hazard ratios for various safety events. For acute liver injury (ALI), additional analyses and medical record validation were conducted.

**Results:**

There were 2445 patients who initiated palbociclib including 566 new users of palbociclib-fulvestrant, and 2316 historical new users of fulvestrant monotherapy. Compared to these historical new users of fulvestrant monotherapy, new users of palbociclib-fulvestrant had a greater than 2-fold elevated risk for neutropenia, leukopenia, thrombocytopenia, stomatitis and mucositis, and ALI. Incidence of anemia and QT prolongation were more weakly associated, and incidences of serious infections and pulmonary embolism were similar between groups after propensity score matching. After adjustment for additional ALI risk factors, the elevated risk of ALI in new users of palbociclib-fulvestrant persisted (e.g. primary ALI algorithm hazard ratio (HR) = 3.0, 95% confidence interval (CI) = 1.1–8.4).

**Conclusions:**

This real-world study found increased risks of several adverse events identified in clinical trials, including neutropenia, leukopenia, and thrombocytopenia, but no increased risk of serious infections or pulmonary embolism when comparing new users of palbociclib-fulvestrant to fulvestrant monotherapy. We observed an increased risk of ALI, extending clinical trial findings of significant imbalances in grade 3/4 elevations of alanine aminotransferase (ALT).

**Supplementary Information:**

The online version contains supplementary material available at 10.1186/s12885-021-07790-z.

## Background

Palbociclib was the first cyclin-dependent kinase 4/6 (CDK4/6) inhibitor to receive accelerated approval by the United States (US) Food Drug Administration (FDA) in February 2015 to treat post-menopausal women for advanced stage Hormone Receptor Positive (HR+)/Human Epidermal Growth Factor Negative (HER2-) breast cancer in combination with letrozole as initial endocrine therapy [1]. In February 2016, palbociclib in combination with fulvestrant received US approval for the treatment of women with HR+/HER2- advanced or metastatic breast cancer with disease progression following endocrine therapy [1].

Randomized-controlled trials (PALOMA 2/3) [2, 3] demonstrated that palbociclib prolonged progression-free survival by 5 to 10 months when used in combination with letrozole or fulvestrant compared to endocrine monotherapy. However, adverse events (AEs) and discontinuations due to AEs were more frequent in the palbociclib (with endocrine therapy) arms compared to the placebo-controlled (with endocrine therapy) arms in PALOMA2 (9.7% vs. 5.9%) and PALOMA3 (2.6% vs. 1.7%) [2, 3]. Some of the most commonly occurring (> 10%) AEs in the palbociclib arms of the trials included neutropenia, infections, leukopenia, and anemia [2, 3]. These trials also revealed an increased risk of grade 3/4 elevations of alanine aminotransferase (ALT), with palbociclib – but were limited in size and breast cancer disease severity in evaluating less common AEs such as acute liver injury (ALI) [1–3]. This study’s objectives were to describe the characteristics of new palbociclib users and to evaluate its safety under real world conditions. Specific safety events of interest were evaluated comparing new users of palbociclib-fulvestrant with historical new users of fulvestrant monotherapy. Based on the initial results, further analyses were conducted to further assess ALI and to validate ALI using medical records.

## Methods

### Study population and design

This new user cohort study was conducted using the HealthCore Integrated Research Database (HIRD). The HIRD includes claims for over 50 million commercially insured health plan members from across the US. Patient enrollment data, inpatient and outpatient medical care, and outpatient prescription drug use are tracked longitudinally for each patient.

This study includes descriptive analyses of all palbociclib new users in the HIRD, and comparative analyses of various pre-specified safety events. The incidence of safety events was evaluated in three subgroups of palbociclib users from 01 February 2015 until 30 September 2017: 1) new users of palbociclib-letrozole, 2) new users of palbociclib-fulvestrant, and 3) all other new users of palbociclib (i.e., past fulvestrant/letrozole use or no fulvestrant/letrozole use at time of palbociclib initiation). These palbociclib groups each required individuals to be at least 18 years of age and have at least 3 months of health plan coverage before initiating palbociclib (without prior use of a CDK 4/6 inhibitor (Supplemental Table 2).

For the comparative analyses, we contrasted one of the palbociclib groups, new users of palbociclib-fulvestrant, to new users of fulvestrant monotherapy, because these regimens have similar indications (disease progression following endocrine therapy) and were expected to have good comparability. In contrast, letrozole monotherapy is approved for treating patients with early stage disease, and thus deemed an unfit comparator for the palbociclib-letrozole group [4]. The comparator group in this study included individuals who were newly dispensed fulvestrant monotherapy from 01 January 2011 until 31 January 2015, before palbociclib was available and met inclusion criteria noted for the palbociclib groups (along with requiring ≥3 months with no dispensing of fulvestrant prior to the index date; Supplemental Table 2).

This historical comparator group was chosen owing to enhance comparability with palbociclib-fulvestrant. After palbociclib became available, the decision to add palbociclib versus initiating fulvestrant monotherapy could be related to differences in patient characteristics potentially related to risk of ALI, such as severity of disease.

### Follow-up and exposure classification

For each treatment group, the start of follow-up (index date) was the day after the date of the first dispensing of palbociclib or, for the comparator group, fulvestrant, with a requirement that they had no palbociclib or fulvestrant dispensings in at least the prior 3 months during the study period. Treatment episodes started on the dispensing date, and continued for the number of days supplied, plus 30 days to account for possible non-concordance of dispensing date and administration. Consecutive dispensings defined in this manner were concatenated into a single continuous treatment episode. Treatment episodes were discontinued after a 30-day gap period without another dispensing or, for palbociclib, after switching to another CDK4/6 inhibitor. If a patient re-initiated palbociclib or fulvestrant monotherapy without a prior censoring/safety event, their subsequent “treated” person-time after re-initiation (treatment episode(s)) was also included. This affected 13% of palbociclib-fulvestrant patients and 27% of historical fulvestrant monotherapy patients. Patients were followed while treated until the earliest of the following dates: end of study period (30 September 2017 for palbociclib, or 01 February 2015 for fulvestrant monotherapy), end of continuous health plan enrollment, or at the end of all palbociclib or fulvestrant monotherapy treatment episode(s). Follow-up also ended on the date of any occurrence of a safety event being analyzed (as defined in claims). For analysis of each safety event, follow-up was not truncated due to occurrence of another safety event.

### Safety events

To identify safety events, we used algorithms based on the International Classification of Diseases, Ninth and Tenth Revisions (ICD-9/10) diagnosis codes and procedure codes associated with insurance claims (defined in Supplemental Table 1). Many of the algorithms were designed to reduce the possibility of missing a case. Safety events included: neutropenia, febrile neutropenia, leukopenia, alopecia, vomiting, QT prolongation, fatigue, various forms of infection, diarrhea, interstitial lung disease/pneumonitis, anemia, nausea, thrombocytopenia, pulmonary embolism, venous embolism and thrombosis, embolism and thrombosis of unspecified artery, cataracts and other ocular disorders, stomatitis and mucositis, fever, anorexia, peripheral neuropathy, sudden cardiac death, diabetes mellitus, type 2 diabetes mellitus, hyperglycemia, ALI, elevated ALT, elevated aspartate amino transferase (AST), abnormal alkaline phosphatase (ALP), second primary malignancies, and non-melanoma skin cancer. For certain safety events of interest (neutropenia, febrile neutropenia, leukopenia, and anemia) a second more specific algorithm that added more stringent criteria to reduce false-positive errors was also evaluated.

Unless otherwise noted, individuals with a history of a particular safety event (defined by the same algorithm) on or prior to the index date were excluded from the computation of incidence for that event. Certain commonly re-occurring safety events (e.g. nausea, diarrhea) allowed a history of these events on or prior to the index date. These events are all defined in supplemental Table 3.

### Additional analyses for ALI

After identifying an increased risk of ALI among new users of palbociclib-fulvestrant, we conducted additional activities including: development of multiple case definitions (ALI algorithms), medical record validation of ALI algorithms, further control for potential confounding using ALI risk factors, and the addition of a contemporaneous comparator group.

Validation studies of claims algorithms for ALI have reported low sensitivity or specificity with positive predictive values (PPVs) as low as 25% [5], so we used multiple algorithms for ALI to assess the robustness of the results with respect to ALI definition. The original ALI algorithm included codes for elevated liver enzymes as well as liver necrosis (Supplemental Table 1). After further review, we used a primary ALI algorithm derived from another safety study [6] and a Mini-Sentinel validation study [5] that restricted to inpatient discharge diagnoses for ALI and acute liver failure (not elevated liver enzymes). Inpatient diagnoses identify more serious events and are considered more reliable than outpatient diagnoses which can include rule-out and presumptive diagnoses. We also developed a more sensitive ALI algorithm that included a broader set of codes in both inpatient and outpatient settings [5, 7], and a more specific ALI algorithm restricting to primary inpatient codes that had a high PPV in the Mini-Sentinel validation study (algorithms defined in Supplemental Table 1) [5].

.Medical record validation of potential ALI cases identified by all four ALI claims algorithms involved review of medical records by at least two independent hepatologists with expertise in drug-induced liver injury. We sought medical records for all potential ALI cases identified by the claims algorithms. Hepatologists who were blinded to study treatment adjudicated potential cases as confirmed ALI or non-ALI based on criteria that required elevation in at least one liver enzyme test (e.g., ALT >3x ULN), specific timing of the enzyme tests, and absence of chronic liver disease [8, 9]. Potential ALI cases that were reviewed but lacked sufficient information in the medical record for adjudication were classified as provisional cases.

We included a contemporaneous comparator of fulvestrant monotherapy which, although smaller and more prone to channeling (i.e. likely to include less severe patients), would not be affected by possible temporal trends (e.g., ICD-9 to ICD-10 code transition in October 2015) that might affect the historical comparator group [10]. The contemporaneous comparator included new users of fulvestrant monotherapy between 01 February 2015 until 30 September 2017, with follow-up censored on the date of initiation of palbociclib or on the date of another previously described censoring criterion.

### Statistical analyses

Study population characteristics were described using measures of central tendency (mean, standard deviation, median, and interquartile range) for continuous variables and frequencies for categorical variables. To assess comparability between treatment groups, absolute standardized differences were computed for each covariate [11].

The incidence of the safety events was calculated for each event in each treatment group by dividing the number of events by the person-time at-risk accumulated in the treatment group. Hazard ratios (HRs) were calculated using Cox proportional hazard regression models, and 95% confidence intervals (CIs) for the incidence estimates [12]. Propensity score matching was used to control for potential confounders [13]. A logistic regression model for propensity score development included covariates that are important predictors of the outcomes of interest [13]. Variables available for inclusion in the propensity score included demographics, medical history, imaging, breast cancer treatments, healthcare utilization, co-morbidities and non-breast cancer related medication within 6 months of palbociclib or fulvestrant initiation [14].

The distributions of propensity scores for each treatment group were examined, and patients having propensity scores outside the region of overlap between the comparison groups were excluded (trimmed) [15]. We stratified the population by propensity score decile using the distribution of propensity scores in the palbociclib-fulvestrant population. Each palbociclib-fulvestrant new user was then matched by propensity score stratum to one new user of fulvestrant monotherapy [15]. Variables incorporated in the propensity score for initial analyses included age, region, Deyo-Charlson Index, number of outpatient visits, number of emergency room visits, secondary malignancy (i.e. metastases) to lymph nodes of head, face, and neck, secondary malignancy to other specified sites, secondary malignancy to respiratory and digestive sites (which includes liver metastases), tamoxifen, everolimus, anastrazole, denosumab or pamidronate, exemestane, chemotherapy, corticosteroids, diagnostic imaging, breast cancer surgery, letrozole, HER2 positive therapy, radiation therapy, CT imaging, mammography, MRI imaging, anticonvulsants, antidepressants, sedatives/hypnotics, secondary malignancy to breast, breast cancer diagnosis code, in situ breast cancer diagnosis, hyperglycemia, and cerebrovascular disease.).

To further evaluate ALI, we created a propensity score that included additional baseline covariates that may be associated with ALI. The ALI propensity score included the previously described variables along with chronic liver disease or alcoholism, chronic or acute disease of gallbladder or pancreas, hepatic, biliary or pancreatic cancer, congestive heart failure, and medications associated with ALI (acetaminophen, allopurinol, amiodarone, amitriptyline, + clavulanic acid, aripiprazole, baclofen, ciprofloxacin, clindamycin, clopidogrel, duloxetine, estrogens, fluoxetine, ketoconazole, lisinopril, losartan, mirtazapine, Nitrofurantoin, NSAIDs, omeprazole, paroxetine, phenothiazine, sertraline, statins, tetracycline, trazodone, and trimethoprim). The development of the ALI propensity scores was the same as described above with the exception that strata were defined by propensity score quartiles to improve ability to identify matching comparators.

In sensitivity analyses, we examined the potential effect of unmeasured confounding by calculating the E-value (the associations between the confounder-exposure and the confounder-outcome needed to attenuate the association of interest to a level indicating no effect (HR) = 1.0)) [16]. We also examined the possible impact of outcome misclassification of ALI on effect estimates using hypothetical values of misclassification rates [17].

## Results

### Descriptive analyses

From February 2015 to September 2017, 2795 individuals received at least one dispensing of palbociclib; 2445 of whom met inclusion criteria which required individuals to be at least 18 years of age, have at least 3 months of health plan coverage, and at least 3 months with no dispensing of palbociclib or CDK 4/6 inhibitor prior to the index date of new palbociclib use (Supplemental Table 2). Among the 2445 eligible new users of palbociclib, there were 566 new users of palbociclib-fulvestrant, 1159 new users of palbociclib-letrozole, and 720 other new users of palbociclib. There were 2316 eligible individuals who received at least one dispensing of fulvestrant during the historical comparator period from January 2011–January 2015 and met inclusion criteria (including ≥3 months with no dispensing of fulvestrant prior to the index date; Supplemental Table 2).

The three palbociclib subgroups were similar at baseline (Table 1). Most palbociclib initiators were between the ages of 45–64 (60.9%), previously used an aromatase inhibitor (62.5%), had a secondary malignancy/metastasis prior to the index date (87.4%), and had advanced stage ER+/HER2- breast cancer (93.5%) [18].Most patients were female, although 53 males (2.2%) were dispensed palbociclib. Healthcare utilization in the previous 6 months was common (mean number of outpatient visits = 39.7), but surgery (mastectomy/lumpectomy), chemotherapy, and radiation therapy during the same period were less common (each < 20%). The most common non-breast cancer related medications received prior to palbociclib initiation included antidepressants (30.5%), antihypertensives (26.7%), and corticosteroids (24.8%). Common co-morbidities included cerebrovascular disease, pure hypercholesterolemia, pathologic fracture, and osteoporosis (each > 8.0%).

**Table 1:** Select Characteristics of New Users of Palbociclib Identified in the HIRD

Characteristics^**a**^	All new users of palbociclib		New users of palbociclib-fulvestrant		New users of palbociclib-letrozole		All other new users of palbociclib	
	N/Mean	%/SD	N/Mean	%/SD	N/Mean	%/SD	N/Mean	%/SD
**Overall**	**2445**	**100%**	**566**	**100%**	**1159**	**100%**	**720**	**100%**
Total duration of follow-up of cohort (in years)	1540		324		812		404	
Duration of follow-up (in years)	0.63	0.53	0.57	0.43	0.70	0.58	0.56	0.50
Age at index date (in years)	59.79	11.62	59.47	11.38	59.21	11.27	60.95	12.29
Sex								
Male	53	2.17	< 10	n/a	14	1.21	30	4.17
Female	2392	97.83	557	98.41	1145	98.79	690	95.83
Calendar year of index date								
2015	791	32.35	99	17.49	456	39.34	236	32.78
2016	942	38.53	269	47.53	406	35.03	267	37.08
2017	712	29.12	198	34.98	297	25.63	217	30.14
Geographic region of residence								
Midwest	400	16.36	95	16.78	185	15.96	120	16.67
South	581	23.76	159	28.09	288	24.85	134	18.61
Northeast	696	28.47	166	29.33	340	29.34	190	26.39
West	768	31.41	146	25.80	346	29.85	276	38.33
Secondary malignancy to any site (metastasis)	2137	87.40	496	87.63	1044	90.08	597	82.92
Secondary malignancy to Lymph nodes of head, face, and neck metastasis	690	28.22	154	27.20	356	30.72	180	25.00
Secondary malignancy to Respiratory and digestive system metastasis (includes liver metastasis)	1058	43.27	257	45.41	490	42.28	311	43.19
Secondary malignancy to Metastasis to other specified sites	2017	82.49	463	81.80	978	84.38	576	80.00
Deyo-Charlson comorbidity index (DCI)	8.39	1.90	8.52	1.76	8.48	1.70	8.13	2.25
Advanced stage ER+/HER2- breast cancer	2285	93.46	548	96.82	1083	93.44	654	90.83
Radiation therapy	483	19.75	112	19.79	233	20.10	138	19.17
Chemotherapy	472	19.30	100	17.67	218	18.81	154	21.39
CT related imaging	602	24.62	139	24.56	333	28.73	130	18.06
Number of outpatient visits	39.67	25.20	38.58	23.63	39.51	23.78	40.79	28.42
Aromatase inhibitor	1527	62.45	326	57.60	815	70.32	386	53.61
HER2+ therapy	72	2.94	15	2.65	35	3.02	22	3.06
Tamoxifen	552	22.58	140	24.73	270	23.30	142	19.72
Fulvestrant	621	25.40	238	42.05	112	9.66	271	37.64
Denosumab or pamidronate	836	34.19	206	36.40	359	30.97	271	37.64
Everolimus	150	6.13	40	7.07	52	4.49	58	8.06
Antihypertensives	653	26.71	173	30.57	293	25.28	187	25.97
Corticosteroids	606	24.79	148	26.15	308	26.57	150	20.83
Lipid lowering agent	528	21.60	132	23.32	251	21.66	145	20.14
Pathologic fracture	211	8.63	46	8.13	110	9.49	55	7.64
Pure hypercholesterolemia	215	8.79	48	8.48	105	9.06	62	8.61

*Abbreviations*: *HIRD* HealthCore Integrated Research Database, *N* number, *SD* standard deviation, *ER* estrogen receptor, *HER2* human epidermal growth factor receptor 2, *CT* computed tomography

^a^All characteristics are measured as presence within six months prior to palbociclib initiation, unless otherwise specified

The incidence rates of safety events after the initiation of palbociclib among all new users are described in Supplemental Table 3. Safety events common to palbociclib users after initiation included neutropenia, anemia, interstitial lung disease/pneumonitis, and serious infections (each incidence rate > 20 per 100 person-years). Less common safety events included sudden cardiac death, stomatitis and mucositis, febrile neutropenia, and ALI (each incidence rate < 5 per 100 person-years).

### Comparative analyses

Descriptive characteristics of the historical comparator group of new users of fulvestrant monotherapy are provided in Table 2 and Supplemental Table 4. Before propensity score matching, new users of palbociclib-fulvestrant (*n* = 565; person-years of follow-up = 322) and the historical comparator group of new users of fulvestrant monotherapy (*n* = 2316, person-years of follow-up = 1686) were similar on many factors related to cancer therapy history, such as chemotherapy and radiation therapy, specific types of hormone therapy use, and most co-morbidities (Table 2). Regarding differences between the groups, new fulvestrant users were older than palbociclib-fulvestrant users (mean ages 64 vs. 59), while the palbociclib-fulvestrant users were more likely to have a secondary malignancy (metastases) to respiratory and digestive systems (45.4% vs. 33.7%) and other specified sites (81.8% vs. 73.8%), a higher DCI score (8.5 vs. 7.9), and use of certain breast cancer medications, such as tamoxifen, everolimus, denosumab, or pamidronate (Table 2). After propensity score matching, these factors were balanced with standardized differences < 0.10 (Table 2 and Supplemental Table 4).

**Table 2:** Select characteristics of new users of palbociclib-fulvestrant and new users of fulvestrant monotherapy (historical comparator group) before and after propensity score matching

Characteristics^**a**^	Before Propensity Score Matching					After Propensity Score Matching^**b**^				
	New users of palbociclib-fulvestrant		New users of fulvestrant monotherapy (pre-2015)		Std. difference	New users of palbociclib-fulvestrant		New users of fulvestrant monotherapy (pre-2015)		Std. difference
	N/Mean	%/SD	N/Mean	%/SD		N/Mean	%/SD	N/Mean	%/SD	
**Overall**	**566**	**100%**	**2316**	**100%**		**561**	**100%**	**561**	**100%**	
Total duration of follow-up (in years)	324		1686			322		373		
**Demographics**										
Age at index date (in years)	59.3	11.0	64.1	12.9	0.4	59.5	11.0	59.9	13.3	0.04
Sex										
Male	≤10	n/a	30	1.30	0.0	≤10	n/a	≤10	n/a	0.10
Female	557	98.4	2286	98.7	0.0	552	98.4	558	99.5	0.10
Geographic region of residence										
Midwest	95	16.8	422	18.2	0.04	95	16.9	93	16.6	0.01
South	159	28.1	690	29.8	0.04	157	28.0	165	29.4	0.03
Northeast	166	29.3	580	25.0	0.10	164	29.2	158	28.2	0.02
West	146	25.8	624	26.9	0.03	145	25.8	145	25.8	0.00
**Medical History**										
Other primary cancer prior to first breast cancer diagnosis code	223	39.4	1026	44.3	0.1	222	39.6	271	48.3	0.18
Secondary malignancy (metastasis)	496	87.6	1823	78.7	0.2	491	87.5	488	87.0	0.02
Lymph nodes of head, face, and neck	154	27.2	476	20.6	0.2	151	26.9	150	26.7	0.00
Respiratory and digestive systems	257	45.4	781	33.7	0.2	254	45.3	252	44.9	0.01
Other specified sites	463	81.8	1710	73.8	0.2	458	81.6	458	81.6	0.00
Deyo-Charlson comorbidity index (DCI) without cancer codes	8.5	1.8	7.85	2.3	0.3	8.5	1.8	8.56	1.6	0.03
**Cancer Therapy History**										
**Radiation therapy**	112	19.8	386	16.7	0.1	112	20.0	117	20.9	0.02
**Surgery**	11	1.9	68	2.9	0.1	11	2.0	13	2.3	0.02
**Chemotherapy**	100	17.7	429	18.5	0.0	99	17.6	98	17.5	0.00
**Imaging**										
CT related imaging in the last 6 months	139	24.6	501	21.6	0.1	138	24.6	146	26.0	0.03
Diagnostic imaging in the last 6 months	72	12.7	461	19.9	0.2	72	12.8	79	14.1	0.04
MRI related imaging	25	4.4	91	3.9	0.0	25	4.5	26	4.6	0.01
**Healthcare Utilization (6 months prior to index date)**										
Number of outpatient visits	37.6	21.7	36.5	24.6	0.0	37.7	21.8	39.3	24.2	0.07
Number of inpatient hospitalizations	0.3	0.7	0.3	0.7	0.0	0.3	0.7	0.4	0.8	0.14
Number of emergency department visits	0.3	0.8	0.2	0.6	0.1	0.3	0.8	0.3	0.7	0.03
**Medication Use (breast cancer related)**										
Palbociclib	0	0.00	0	0.00	n/a	0	0.00	0	0.00	n/a
Aromatase inhibitor	326	57.6	1285	55.5	0.04	321	57.2	303	54.0	0.06
Letrozole	116	20.5	429	18.5	0.05	113	20.1	116	20.7	0.01
Anastrazole	136	24.0	611	26.4	0.05	135	24.1	138	24.6	0.01
Exemestane	86	15.2	358	15.5	0.01	85	15.2	76	13.5	0.05
HER2 positive therapy	15	2.7	127	5.5	0.14	15	2.7	14	2.5	0.01
Trastuzumab	25	4.4	159	6.9	0.11	24	4.3	27	4.8	0.03
Lapatinib	≤10	n/a	16	0.7	0.05	≤10	n/a	≤10	n/a	0.03
Ado-trastuzumab	0	0.0	0	0.0	n/a	0	0.0	0	0.0	n/a
Pertuzumab	0	0.0	0	0.0	n/a	0	0.0	0	0.0	n/a
Tamoxifen	140	24.7	379	16.4	0.21	140	25.0	135	24.1	0.02
Fulvestrant	238	42.0	0	0.0	n/a	236	42.1	0	0.0	n/a
Denosumab or Pamidronate	206	36.4	424	18.3	0.41	201	35.8	197	35.1	0.01
Everolimus	40	7.1	84	3.6	0.15	39	7.0	37	6.6	0.01
**Medication Use (not breast cancer related)**										
Anticonvulsants	126	22.3	347	15.0	0.19	122	21.7	123	21.9	0.00
Antidepressants	178	31.4	631	27.2	0.09	175	31.2	184	32.8	0.03
Antihypertensives	173	30.6	623	26.9	0.08	171	30.5	135	24.1	0.14
Antimycobacterials	109	19.3	446	19.3	0.00	107	19.1	117	20.9	0.04
Antivirals	32	5.7	112	4.8	0.04	32	5.7	30	5.3	0.02
Corticosteroids	148	26.1	416	18.0	0.20	146	26.0	146	26.0	0.00
Lipid lowering agent	132	23.3	556	24.0	0.02	130	23.2	121	21.6	0.04
Sedatives/hypnotics	57	10.1	291	12.6	0.08	57	10.2	63	11.2	0.03
**Co-morbidities (6 months prior to index date)**										
Pathologic fracture	46	8.1	173	7.5	0.02	46	8.2	51	9.1	0.03
Major adverse cardiac events (MACE)	20	3.5	83	3.6	0.00	20	3.6	23	4.1	0.03
Cerebrovascular disease	301	53.2	1148	49.6	0.07	296	52.8	277	49.4	0.07
Hyperglycemia	27	4.8	55	2.4	0.13	23	4.1	29	5.2	0.05
Deyo-Charlson Index (DCI)										
0–3	19	3.4	259	11.2	0.30	19	3.4	17	3.0	0.02
4–7	12	2.1	57	2.5	0.02	12	2.1	4	0.7	0.12
8–11	512	90.5	1937	83.6	0.20	508	90.6	517	92.2	0.06
12 or more	23	4.1	63	2.7	0.07	22	3.9	23	4.1	0.01
**Risk factors associated with ALI**										
Chronic liver disease or Alcoholism^c^	74	13.1	246	10.6	0.09	74	13.1	69	12.2	0.03
Chronic or acute disease of gallbladder or pancreas^c^	36	6.4	145	6.3	0.01	36	6.4	34	6.0	0.01
Hepatic, Biliary or pancreatic cancer^c^	160	28.3	458	19.8	0.18	160	28.3	143	25.3	0.07
Congestive heart failure^c^	29	5.1	159	6.9	0.07	29	5.1	29	5.1	0.00
Any medication associated with liver failure^c^	467	82.51	1749	75.52	0.17	466	82.48	466	82.48	0.00
Acetaminophen (prescription) ^c^	204	36.04	899	38.82	0.06	204	36.11	202	35.75	0.01

*Abbreviations*: *ER* estrogen receptor, *HER2* human epidermal growth factor receptor 2, *N* number, *SD* standard deviation, *Std* standardized, *CT* computed tomography, *MRI* magnetic resonance imaging

^a^All characteristics are measured as presence within 6 months prior to the index date, unless otherwise specified

^b^The following variables were included in the propensity score for all safety event analyses: age, region, Deyo-Charlson Index, number of outpatient visits, number of emergency room visits, secondary malignancy to lymph nodes of head, face, and neck, secondary malignancy to other specified sites, secondary malignancy to respiratory sites, tamoxifen, everolimus, anastrazole, denosumab or pamidronate, exemestane, chemotherapy, corticosteroids, diagnostic imaging, breast cancer surgery, letrozole, HER2 positive therapy, radiation therapy, CT imaging, mammography, MRI imaging, anticonvulsants, antidepressants, sedatives/hypnotics, secondary malignancy to breast, breast cancer diagnosis code, in situ breast cancer diagnosis, hyperglycemia, and cerebrovascular disease

^c^The following variables were included in the propensity score specific for subsequent ALI analyses (conducted after observing an association with ALI in initial analyses): age, region, Deyo-Charlson Index, number of outpatient visits, number of emergency room visits, secondary malignancy to lymph nodes of head, face, and neck, secondary malignancy to other specified sites, secondary malignancy to respiratory sites, tamoxifen, everolimus, anastrazole, denosumab or pamidronate, exemestane, chemotherapy, corticosteroids, diagnostic imaging, breast cancer surgery, letrozole, HER2 positive therapy, radiation therapy, CT imaging, mammography, MRI imaging, anticonvulsants, antidepressants, sedatives/hypnotics, secondary malignancy to breast, breast cancer diagnosis code, in situ breast cancer diagnosis, hyperglycemia, cerebrovascular disease, Chronic liver disease or Alcoholism, Chronic or acute disease of gallbladder or pancreas, Hepatic, Biliary or pancreatic cancer, Congestive heart failure, any medication associated with ALI- Acetaminophen, Allopurinol, Amiodarone, Amitriptyline, + clavulanic acid, Aripiprazole, Baclofen, Ciprofloxacin, Clindamycin, Clopidogrel, Duloxetine, Estrogens, Fluoxetine, Ketoconazole, Lisinopril, Losartan, Mirtazapine, Nitrofurantoin, NSAIDs, Omeprazole, Paroxetine, Phenothiazine, Sertraline, Statins, Tetracycline, Trazodone, and Trimethoprim

When compared to a propensity score-matched historical group of new users of fulvestrant monotherapy (*n* = 561), new users of palbociclib-fulvestrant (*n* = 561) were more likely to develop neutropenia (HR = 7.8, 95% CI = 4.7–13.0), leukopenia (HR = 6.4, 95% CI = 1.9–21.9), stomatitis and mucositis (HR = 5.0, 95% CI = 1.1–23.1), ALI (HR = 4.8, 95% CI = 1.4–16.9), anemia (HR = 1.8, 95% CI = 1.4,-2.3), and QT prolongation (HR = 1.8, 95% CI = 0.9–3.5) (Table 3). Incidence rates of other safety event rates were similar among new users of palbociclib-fulvestrant and new users of fulvestrant monotherapy, including serious infections, type 2 diabetes mellitus, second primary cancers, and pulmonary embolism (Table 3 and Supplemental Table 5).

**Table 3:** Select^a^ Incidence Rates and Propensity Score Adjusted^b^ Hazard Ratios of the Safety Events Comparing New Users of Palbociclib and Fulvestrant and New Users of Fulvestrant Monotherapy

Safety Event^**c**^	After Propensity Score Matching^**b**^								
	New users of palbociclib-fulvestrant (***n*** = 561)			Historical new users of fulvestrant monotherapy (***n*** = 561)			Hazard Ratio Estimates		
	IR (per 100 person-years)			IR (per 100 person-years)			HR	95% LCL	95% UCL
	IR	95% LCL	95% UCL	IR	95% LCL	95% UCL			
Anemia	47.7	40.0	56.5	26.2	21.1	32.2	1.8	1.4	2.3
Neutropenia	36.7	30.0	44.5	4.6	2.7	7.4	7.8	4.7	13.0
Serious infection	25.7	20.4	32.1	22.9	18.2	28.5	1.1	0.8	1.5
Thrombocytopenia	10.5	7.2	14.7	4.6	2.7	7.4	2.3	1.3	4.1
QT prolongation	6.4	3.9	9.9	3.5	1.9	6.0	1.8	0.9	3.5
Leukopenia	5.4	3.1	8.6	0.8	0.2	2.4	6.4	1.9	21.9
Pulmonary embolism	4.4	2.4	7.4	4.1	2.3	6.7	1.0	0.5	2.1
Acute liver injury (ALI)^c^	4.1	2.2	6.9	0.8	0.2	2.4	4.8	1.4	16.9
Stomatitis and mucositis	2.8	1.3	5.3	0.5	0.1	1.7	5.0	1.1	23.1

*Abbreviations*: *IR* incidence rate, *LCL* lower confidence limit, *UCL* upper confidence limit, *HR* hazard ratio

^a^Results for all safety events are provide in supplemental Table 5. Safety events selected for this table include notable safety events on the palbociclib product label or those of particular interest

^b^The following variables were included in the propensity score: age, region, Deyo-Charlson Index (DCI), number of outpatient visits, number of emergency room visits, secondary malignancy (i.e. metastases) to lymph nodes of head, face, and neck, secondary malignancy to other specified sites, secondary malignancy to respiratory and digestive sites (including to the liver), tamoxifen dispensing, everolimus dispensing, anastrazole dispensing, denosumab or pamidronate dispensing, exemestane dispensing, chemotherapy dispensing, corticosteroids dispensing, diagnostic imaging, breast cancer surgery, letrozole dispensing, HER2 positive therapy, radiation therapy, CT (computed tomography) imaging, mammography, MRI (magnetic resonance imaging), anticonvulsants, antidepressants, sedatives/hypnotics, secondary malignancy to breast, breast cancer diagnosis code, in situ breast cancer diagnosis, hyperglycemia, and cerebrovascular disease

^c^All algorithm definitions are provided in Supplemental Table 1. The “original” ALI algorithm for this active surveillance study is noted in this table as the “ALI or elevation of transaminases (ALI definition 2)”

### Additional ALI analyses

Before propensity score matching, several ALI risk factors were more prevalent in the palbociclib-fulvestrant group than the fulvestrant monotherapy group (e.g., medication associated with ALI: 82.5% vs. 75.5%; chronic liver disease or alcoholism: 13.1% vs. 10.6%). After propensity score matching, covariates identified as important risk factors for ALI were balanced between the two groups (standardized differences< 0.10; Table 2 and Supplemental Table 6).

**Fig. 1:**
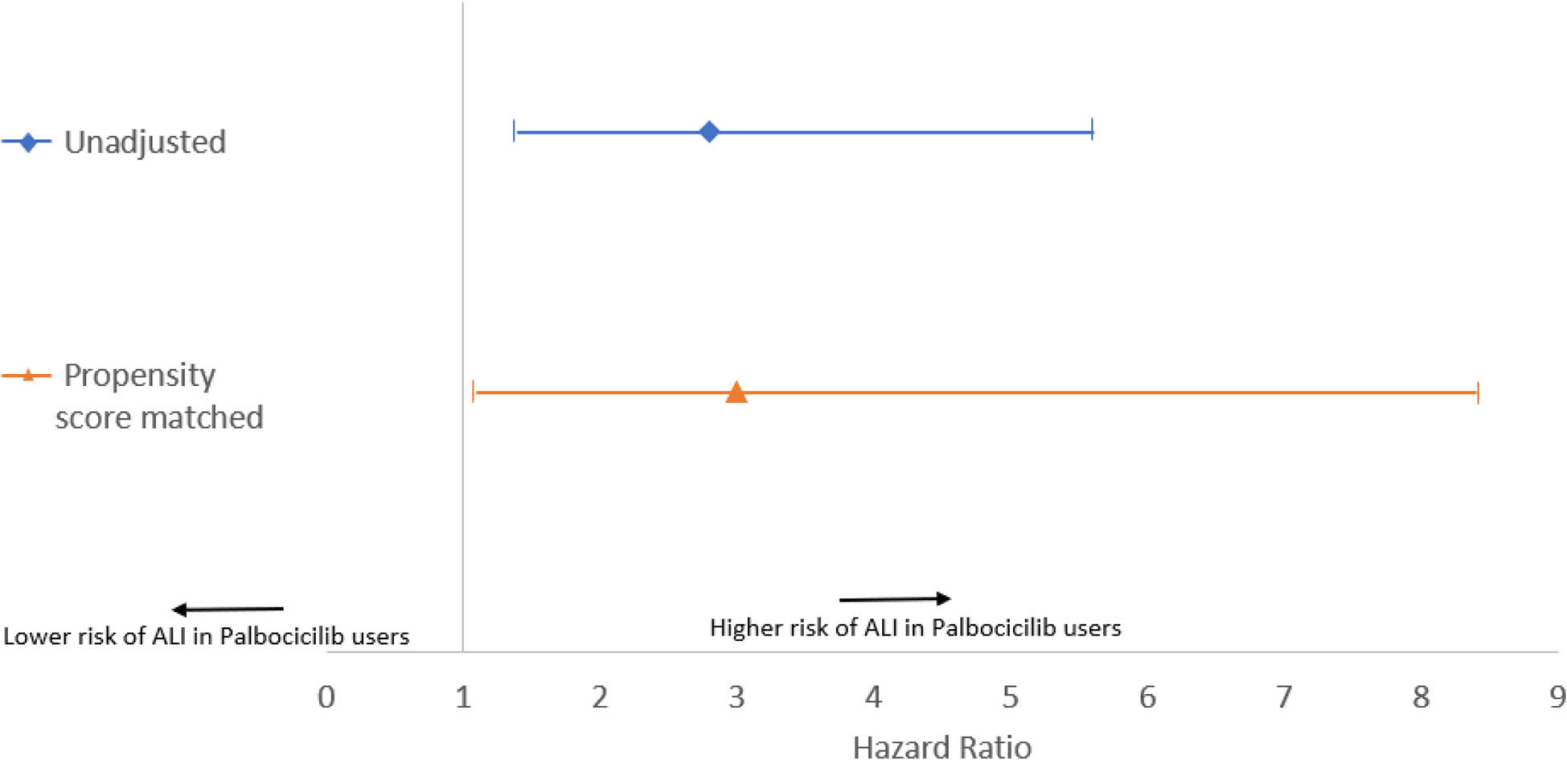
Hazard Ratios of Acute Liver Injury (ALI)^#^ in New Users of Palbociclib-Fulvestrant vs. New Users of Fulvestrant Monotherapy (historical comparator group)^*. ^#^ALI defined using the “primary ALI algorithm” defined in Supplemental Table 1. ^The unadjusted analysis was a comparison prior to propensity score matching. *The propensity score included the following variables: age, region, DCI, number of outpatient visits, number of emergency room visits, secondary malignancy to lymph nodes of head, face, and neck, secondary malignancy to other specified sites, secondary malignancy to respiratory sites, tamoxifen, everolimus, anastrazole, denosumab or pamidronate, exemestane, chemotherapy, corticosteroids, diagnostic imaging, breast cancer surgery, letrozole, HER2+ therapy, radiation therapy, CT imaging, mammography, MRI imaging, anticonvulsants, antidepressants, sedatives/hypnotics, secondary malignancy to breast, breast cancer diagnosis code, in situ breast cancer diagnosis, hyperglycemia, cerebrovascular disease, chronic liver disease or Alcoholism, chronic or acute disease of gallbladder or pancreas, hepatic, biliary or pancreatic cancer, congestive heart failure, any medication associated with ALI, including acetaminophen, allopurinol, amiodarone, amitriptyline, clavulanic acid, aripiprazole, baclofen, ciprofloxacin, clindamycin, clopidogrel, duloxetine, estrogens, fluoxetine, ketoconazole, lisinopril, losartan, mirtazapine, nitrofurantoin, NSAIDs, omeprazole, paroxetine, phenothiazine, sertraline, statins, tetracycline, trazodone, and trimethoprim

Prior to propensity score matching we observed an elevated risk for the primary ALI endpoint, (unadjusted HR = 2.8, 95% CI = 1.4, 5.6; Fig. 1). After propensity score matching, there were 18 cases of ALI in both groups combined using the primary algorithm, 20 cases using the original algorithm (including elevated aminotransferases), 49 cases using the sensitive algorithm, and 0 cases using the specific algorithm. For the primary ALI endpoint, the incidence was 4.0 per 100 person-years in the palbociclib-fulvestrant group, and 1.2 per 100 person-years in the fulvestrant monotherapy historical comparator group (unadjusted HR = 2.8, 95% CI = 1.4, 5.6; Fig. 1). Effect estimates were imprecise but similarly elevated for each algorithm (Primary algorithm: HR = 3.0, 95% CI = 1.1–8.4; original algorithm: HR = 2.2, 95% CI = 0.9, 5.4; sensitive algorithm: HR = 4.6, 95% CI = 2.3, 9.1 (Supplemental Table 7, and Fig. 1)).

For the contemporaneous fulvestrant comparator group, we identified 961 new users of fulvestrant monotherapy between 01 February 2015 and 30 September 2017. Before matching, the contemporaneous fulvestrant group and the palbociclib-fulvestrant group were imbalanced with regard to several covariates (e.g., 55% vs. 31% greater than age 65, and 35% vs. 45% had metastases to the respiratory and digestive systems; Supplemental Table 8), and we could find matches for only 292 of the palbociclib users.

As a result of the initial imbalance between the palbociclib-fulvestrant users and the contemporaneous comparators, propensity score matching reduced the number of cases available for analysis (e.g. 24 vs. < 10 cases of ALI in the combined treatment groups using the primary algorithm). Before matching, the incidence of ALI using the primary algorithm was 4.0 per 100 person-years in the palbociclib-fulvestrant group and 2.0 per 100 person-years in the fulvestrant monotherapy contemporaneous comparator group. The unadjusted HR was 2.1, 95% CI = 0.9–4.7. Effect estimates were imprecise after propensity score matching (primary algorithm HR = 0.5, 95% CI = 0.1–2.2). Results were similar for the other ALI algorithms (Supplemental Table 9). Additional analyses did not indicate any important temporal trend in incidence of ALI in the HIRD (Supplemental Table 10).

For the ALI validation component of this study, we requested medical records for 138 of the 185 patients meeting any of the ALI algorithms, who did not have health plan-based privacy restrictions; 52 of which were obtained. Among the 29 cases meeting the primary ALI claims algorithm with adjudicated results, 21 were confirmed as ALI cases, the others were confirmed as non-cases or remained provisional due to lack of sufficient information in the record. The PPV of the primary ALI algorithm among confirmed cases and non-cases was 84% (95% CI = 64–95%), while the other two ALI algorithms had lower PPVs (72 and 73%; Supplementary Table 11).

### Sensitivity analyses

To evaluate possible unmeasured confounding, we calculated the E-value, which is the minimum strength of the palbociclib-confounder and the confounder-ALI associations needed to explain the observed association between palbociclib-fulvestrant and ALI (i.e. for residual confounding to attenuate the association to the null value, HR = 1.0). For the association between palbociclib-fulvestrant and ALI in the historical comparator analysis (HR = 3.0, 95% CI = 1.1–8.4), a risk factor would need to have an association of at least relative risk (RR) =5.5 between palbociclib-fulvestrant and the confounder, and between the confounder and ALI to explain the observed association (Supplemental Figure 1).

We evaluated outcome misclassification by adjusting our comparative estimates by PPVs from the validation component of this study. The results adjusting for PPV were similar to the main results (Supplemental Table 12). In a hypothetical scenario where we assume 100% sensitivity and 100% PPV in the fulvestrant monotherapy group, the amount of differential outcome misclassification needed to attenuate the palbociclib-fulvestrant ALI association (HR = 3.0, 95% CI = 1.1–8.4) to the null value indicating no association would require a PPV of 33% in palbociclib-fulvestrant group.

## Discussion

This appears to be the first epidemiologic study of palbociclib safety in a real-world setting that compared new users of palbociclib-fulvestrant to a historical comparator of new users receiving fulvestrant monotherapy, and supports findings from clinical trials that myelosuppression events, anemia, thrombocytopenia, and stomatitis and mucositis are more frequent with palbociclib-fulvestrant use [1–3, 19–21]. The myelosuppression events, neutropenia and leukopenia, were the most strongly associated with palbociclib-fulvestrant use (HRs > 6). In addition, we found a 2-fold increased risk of QT prolongation and a 3-fold increased risk of ALI with palbociclib-fulvestrant use, while not observing increased rates of pulmonary embolism or serious infections.

In the two phase 3 trials of palbociclib, grade 3/4 ALT elevations occurred in 2% of the palbociclib group (19/789 patients with advanced breast cancer) with no grade 3/4 ALT elevations (0/394) in the comparator (endocrine monotherapy) group (*p* = 0.001) [1], despite the fact that baseline liver metastases were somewhat more common in the comparator group [22]. In the PALOMA 3 trial, grade 3 or higher hepatic adverse events included two cases of hepatic failure and one drug-induced liver injury in the palbociclib-fulvestrant arm [*n* = 345], while no such events were included in the comparator arm [*n* = 172] [19]. The increased risk of ALI reported here is consistent with imbalances in grade 3/4 ALT elevations in clinical trials, and has been hypothesized in recent reports [23–26]. Additionally, ribociclib and abemaciclib, the two other approved CDK4/6 inhibitors, carry hepatotoxicity warnings in their US package inserts, suggesting hepatotoxicity may be common to this class of medication [27, 28]. Both these warnings advise performing liver function tests (LFTs) before drug initiation and during therapy [27, 28].

Sensitivity analyses supported a positive association between palbociclib-fulvestrant and ALI with the exception of adjusted analyses using a contemporaneous comparator of fulvestrant monotherapy. Owing to lack of comparability between the palbociclib-fulvestrant group and the contemporaneous comparator, we could find matches for only 292 (52%) palbociclib patients, as compared with 565 (> 99%) palbociclib patients who could be matched to the historical comparators. Further, even after matching, there were still differences between the contemporaneous comparators and the palbociclib users; for example, patients in the contemporaneous comparator group were older than palbociclib-treated patients. The high rates of ALI in the contemporaneous comparator group (twice that of the palbociclib group for the primary algorithm) and imprecise HRs reflect these constraints of small size and noncomparability.

There are several limitations to this study including small sample size, exposure and outcome misclassification, and confounding. Except for ALI, safety outcomes were not validated with medical records, and while propensity scores can control for numerous measured confounders, they do not control for confounding from unmeasured factors unless they are associated with the measured covariates. The secondary malignancy (metastases) codes available indicate only the presence or absence of metastases to broad anatomic sites, but do not provide information on the exact site of metastatic disease, the extent of metastases and/or the metastatic tumor burden. Sensitivity analyses suggested that it would require a particularly strong association with an unmeasured confounder (e.g., extent of metastases, liver involvement, extent of visceral disease, etc.) that is strongly associated with ALI apart from its association with ‘measured risk factors (e.g., presence of metastasis), to explain the observed association seen when using the historical fulvestrant monotherapy comparator. Given that ALI is rare in patients with liver metastases [29–31] and given the balance between groups in presence of metastases after propensity score matching, it seems unlikely that there would be a strong residual association with the extent of metastases capable of accounting for the observed association.

Finally, while this real-world study is larger than any of the palbociclib clinical trials, the sample size is limited for assessing relatively uncommon events. Imprecision can be seen in the width of the confidence intervals, particularly for rare outcomes such as stomatitis and mucositis, and ALI. Replication in larger studies in other populations (e.g. those with a lower socioeconomic status on Medicaid or older individuals on Medicare) would strengthen these findings, provide further clarity on effect sizes, and would be better able to evaluate any possible interactions palbociclib may have with other medications associated with liver toxicity commonly used in those with advanced stage breast cancer. Additionally, given the severity of ALI [32], if these results are corroborated, further study is warranted on ALI’s effect on palbociclib treatment adherence, quality of life, and survival.

## Conclusions

In this real-world study comparing new users of palbociclib-fulvestrant to a historical comparator group of new users of fulvestrant monotherapy, safety events commonly associated with palbociclib use were mostly similar to those identified in randomized trials. An increased risk of ALI is a new finding, although the number of cases was small. This result should be corroborated to better understand the risk in patients treated with palbociclib.

## Supplementary Information

**Additional file 1: Supplemental Table 1**. Code List for Identifying the Safety Events of Interest in the HIRD*. **Supplemental Table 2**. Formation of Study Cohorts. **Supplemental Table 3**. Incidence of Safety Events in New Users of Palbociclib, Overall and by Subcohort. **Supplemental Table 4**. Characteristics of New Users of Palbociclib and Fulvestrant and New Users of Fulvestrant Monotherapy (Historical Comparator Group) Before and After Propensity Score Matching (All evaluated characteristics). **Supplemental Table 5**. Incidence Rates and Adjusted Hazard Ratios of the Safety Events of Interest in Propensity Score Matched New Users of Palbociclib and Fulvestrant and Historical New Users of Fulvestrant Monotherapy. **Supplemental Table 6**. Characteristics of New Users of Palbociclib and Fulvestrant and New Users of Fulvestrant Monotherapy (Historical Comparison Group) Before and After Propensity Score Matching (Including ALI Risk Factors). **Supplemental Table 7**. Unadjusted and Adjusted Hazard Ratios of ALI in New Users of Palbociclib and Fulvestrant and New Users of Fulvestrant Monotherapy (Historical Comparator). **Supplemental Table 8**. Characteristics of New Users of Palbociclib and Fulvestrant and New Users of Fulvestrant Monotherapy (Contemporaneous Comparison Group). **Supplemental Table 9**. Unadjusted and Adjusted Hazard Ratios of ALI in New Users of Palbociclib and Fulvestrant and New Users of Fulvestrant Monotherapy (Contemporaneous Comparator). **Supplemental Table 10**. Incidence of ALI in the HealthCore Integrated Database (HIRD) Between April 2014 and March 2017. **Supplemental Table 11**. ALI Algorithm Signal Refinement – Validation of Claims Algorithms Compared to Medical Record Adjudication. **Supplemental Table 12**. PPV Adjusted Hazard Ratios of ALI in New Users of Palbociclib and Fulvestrant and New Users of Fulvestrant Monotherapy (Historical Comparator). **Supplemental Figure 1**. E-value to Explain the Association Between Palbociclib-Fulvestrant and the Primary ALI algorithm (in Historical Fulvestrant Analyses)

## Data Availability

The datasets generated during and/or analysed during the current study are not publicly available due privacy regulations.

## Abbreviations

HR: Hormone receptor
HER2: Human epidermal growth factor receptor 2
HIRD: HealthCore Integrated Research Database
ALI: Acute liver injury
HR: Hazard ratio
CI: Confidence interval
ALT: Alanine aminotransferase
CDK4/6: Cyclin-dependent kinase 4/6
US: United States
FDA: Food Drug Administration
AEs: Adverse events
ICD-9/10: International Classification of Diseases, Ninth and Tenth Revisions
AST: Aspartate amino transferase
ALP: Abnormal alkaline phosphatase
PPV: Positive predictive value
LFTs: Liver function tests
IRB: Institutional Review Board
N: Number
SD: Standard deviation
ER: Estrogen receptor
CT: Computed tomography
Std: Standardized
MRI: Magnetic resonance imaging
IR: Incidence rate
LCL: Lower confidence limit
UCL: Upper confidence limit
