# Supplementary material for: Real-world safety of palbociclib in breast cancer patients in the United States: a new user cohort study
Source: BMC Cancer. 2021 Jan 25;21:97. doi: 10.1186/s12885-021-07790-z (PMC7831235; doi:10.1186/s12885-021-07790-z)
Supplement: Supplementary file 1 — Additional file 1: Supplemental Table 1. Code List for Identifying the Safety Events of Interest in the HIRD*. Supplemental Table 2. Formation of Study Cohorts. Supplemental Table 3. Incidence of Safety Events in New Users of Palbociclib, Overall and by Subcohort. Supplemental Table 4. Characteristics of New Users of Palbociclib and Fulvestrant and New Users of Fulvestrant Monotherapy (Historical Comparator Group) Before and After Propensity Score Matching (All evaluated characteristics). Supplemental Table 5. Incidence Rates and Adjusted Hazard Ratios of the Safety Events of Interest in Propensity Score Matched New Users of Palbociclib and Fulvestrant and Historical New Users of Fulvestrant Monotherapy. Supplemental Table 6. Characteristics of New Users of Palbociclib and Fulvestrant and New Users of Fulvestrant Monotherapy (Historical Comparison Group) Before and After Propensity Score Matching (Including ALI Risk Factors). Supplemental Table 7. Unadjusted and Adjusted Hazard Ratios of ALI in New Users of Palbociclib and Fulvestrant and New Users of Fulvestrant Monotherapy (Historical Comparator). Supplemental Table 8. Characteristics of New Users of Palbociclib and Fulvestrant and New Users of Fulvestrant Monotherapy (Contemporaneous Comparison Group). Supplemental Table 9. Unadjusted and Adjusted Hazard Ratios of ALI in New Users of Palbociclib and Fulvestrant and New Users of Fulvestrant Monotherapy (Contemporaneous Comparator). Supplemental Table 10. Incidence of ALI in the HealthCore Integrated Database (HIRD) Between April 2014 and March 2017. Supplemental Table 11. ALI Algorithm Signal Refinement – Validation of Claims Algorithms Compared to Medical Record Adjudication. Supplemental Table 12. PPV Adjusted Hazard Ratios of ALI in New Users of Palbociclib and Fulvestrant and New Users of Fulvestrant Monotherapy (Historical Comparator). Supplemental Figure 1. E-value to Explain the Association Between Palbociclib-Fulvestrant and the Primary ALI algorithm (in H [file 12885_2021_7790_MOESM1_ESM.docx]

# **Supplemental Table 1:** Code List for Identifying the Safety Events of Interest in the HIRD*

| **Safety event of interest** | **ICD-9-CM diagnosis code** | **ICD-10 diagnosis code** |
| --- | --- | --- |
| Neutropenia (sensitive)* | 288.00 (neutropenia, unspecified)  288.02 (cyclic neutropenia)  288.03 (drug-induced neutropenia)  288.09 (other neutropenia) | D701 D702 D704  D708  D709 |
| Neutropenia (specific – drug induced only)* | 288.03 (drug-induced neutropenia) | D701 D702 |
| Febrile neutropenia (sensitive)* | Meets the definition for neutropenia (sensitive) specified above AND has a medical claim indicating fever on the same date:  780.60 (fever, unspecified)  780.61 (fever presenting with conditions classified elsewhere) | Meets the definition for neutropenia (sensitive) specified above AND has a medical claim indicating fever on the same date: R509  R502  R5081 |
| Febrile neutropenia (specific – drug induced only)* | Meets the definition for neutropenia (specific) specified above AND has a medical claim indicating fever on the same date:  780.60 (fever, unspecified)  780.61 (fever presenting with conditions classified elsewhere) | Meets the definition for neutropenia (specific) specified above AND has a medical claim indicating fever on the same date: R509  R502  R5081 |
| Leukopenia (sensitive)* | 288.5x (decreased white blood cell count) | D72810  D72818  D72819 |
| Leukopenia (specific)* | 288.50, 288.59 | D72818  D72819 |
| Anemia (sensitive)* | 280.xx (iron deficiency anemias)  281.xx (other deficiency anemias)  283.xx (acquired hemolytic anemia)  284.xx (aplastic anemia and other bone marrow failure syndromes)  285.xx (other and unspecified anemias) | D500, D501, D508, D509, D510, D511, D513, D518, D520, D521, D528, D529, D530, D531, D532, D538, D539, D590, D591, D593, D594, D595, D596, D598, D599, D600, D601, D608, D609, D6101, D6109, D611, D612, D6181, D61810, D61811, D61818, D6182, D6189, D619, D62, D630, D631, D638, D640, D641, D642, D643, D644, D6481, D6489, D649, D512, D592, D613 |
| Anemia (specific)* | 281.3, 281.4, 284.89, 284.1, 284.11, 284.12, 284.19, 284.2, 284.9, 285.22, 285.3 | D530, D531, D611, D612, D6181, D61810, D61811, D61818, D6182, D6189, D619, D630, D6481, |
| Pulmonary embolism* | 415.11, 415.13, 415.19 | I2692, I2699, I2602, I2609 |
| Alopecia | 704.00 (alopecia, unspecified)  704.09 (other alopecia) | L658, L659, L660, L662, L668, L630, L631, L640, L648, L649, L651, L652, L669 |
| Vomiting | 787.01 (nausea with vomiting)  787.03 (vomiting alone) | R1110, R1111, R1112, R112 |
| QT prolongation | 426.82 (long QT syndrome)  427.1x (paroxysmal ventricular tachycardia)  427.4x (ventricular fibrillation and flutter)  427.5x (cardiac arrest)  427.9x (cardiac dysrhythmia, unspecified)  780.2x (syncope and collapse) | I4581, I469, I472, I4901, I4902, I499, R55, I462, I468, I470, I493 |
| Fatigue | 780.71 (chronic fatigue syndrome)  780.79 (other malaise and fatigue) | G933, R531, R5381, R5382, R5383, R530 |
| Serious Infection | Assume any of the specific infections below that occur at an inpatient hospitalization or emergency department visit |  |
| Infections |  |  |
| Brain and spinal cord | 320.xx (bacterial meningitis)  321.xx (meningitis due to other organisms)  323.xx (encephalitis myelitis and encephalomyelitis)  324.xx (intracranial and intraspinal abscess) | B451, G000, G001, G002, G003, G008, G009, G01, G02, G0400, G0401, G042, G0430, G0431, G0432, G0439, G0481, G0489, G090, G091, G053, G054, G060, G061, G062, G092, G92, G07, G374 |
| Pericardial/Myocardial | 420.91 (acute idiopathic pericarditis)  422.92 (septic myocarditis) | I300, I400 |
| Pulmonary* | 491.22 (obstructive chronic bronchitis with acute bronchitis)  493.21(chronic obstructive asthma with status asthmaticus)  480.xx (viral pneumonia)  481.xx (pneumococcal pneumonia)  482.xx (other bacterial pneumonia)  483.xx (pneumonia due to other specified organism)  484.xx (pneumonia in infectious diseases classified elsewhere)  485.xx (bronchopneumonia, organism unspecified)  486.xx (pneumonia, organism unspecified)  487.0x (influenza with pneumonia)  513.0x (abscess of lung)  510.xx empyema | B250, B440, J10.0%, J12.%, J13, J14, J15.%, J16.%, J17, J18.%, J20.%, J85.% |
| Gastrointestinal | 566.xx (abscess of anal and rectal regions)  567.2x (other suppurative peritonitis)  567.9 (unspecified peritonitis) | K610, K611, K613, K650, K651, K652, K659, K50014, K50114, K50814, K50914, K612, K614, |
| Genitourinary/Renal | 112.2x (candidiasis of other urogenital sites)  583.9x (nephritis and nephropathy, not specified as acute or chronic, with unspecified pathologic lesion in kidney)  590.xx (infections of kidney)  595.0x (acute cystitis)  595.3x (trigonitis)  595.4x (cystitis in diseases classified elsewhere)  595.89 (other specified types of cystitis)  595.9x (cystitis, unspecified)  597.0x (urethral abscess)  597.80 (urethritis, unspecified)  599.0x (urinary tract infection, site not specified)  614.xx (inflammatory disease of ovary fallopian tube pelvic tissue and peritoneum)  616.3x (abscess of Bartholin’s gland)  996.64 (infection and inflammatory reaction to indwelling urinary catheter) | N059, N10, N110, N118, N12, N151, N159, N16, N2884, N2885, N2886, N3000, N3001, N3030, N3031, N3080, N3081, N3090, N3091, N340, N341,N342, N390, N7001, N7002, N7003, N7011, N7012, N7013, N7091, N7092, N7093, N730, N731, N732, N733, N734, N736, N738, N739, N751, T8351XA, T8351XD, A5601, A5611, B3741, N069, N079,  N111, N119, N136, N735, N74 |
| Dental | 522.4x (acute apical periodontitis of pulpal origin)  522.5x (periapical abscess without sinus)  522.7x (periapical abscess with sinus) | K044, K046, K047 |
| Ear, Nose and Throat | 380.10 (infective otitis externa, unspecified)  382.xx (suppurative and unspecified otitis media)  383.0x (acute mastoiditis)  383.9x (unspecified mastoiditis)  460.xx (acute nasopharyngitis)  461.xx (acute sinusitis)  462.xx (acute pharyngitis)  473.0x (chronic maxillary sinusitis)  475.xx (peritonsillar abscess)  478.19 (other disease of nasal cavity and sinuses) 478.5x (other diseases of vocal cords)  478.21 (cellulitis of pharynx or nasopharynx) 478.22 (parapharyngeal abscess)  478.24 (retropharyngeal abscess)  527.3x (abscess of salivary gland)  528.00 (stomatitis and mucositis, unspecified)  528.3x (cellulitis and abscess of oral soft tissues)  529.0x (glossitis) | H6000, H6010, H60319,  H60329, H60399, H66009,  H66019, H6613, H6623,  H663X9, H6640, H6690,  H679, H70009, H70019,  H70099, H7090, J00, J0100,  J0110, J0120, J0130, J0140,  J0190, J029, J320, J340, J341, J3489, J36, J390, J391,  K113, K122, K1230, K140,  H6001, H6002, H6003, H6011, H6012, H6013,  H60311, H60312, H60313,  H60321, H60322, H60323,  H60391, H60392, H60393,  H66001, H66002, H66003,  H66004, H66005, H66006,  H66007, H66011, H66012,  H66013, H66014, H66015,  H66016, H66017, H6610,  H6611, H6612, H6620,  H6621, H6622, H663X1,  H663X2, H663X3, H6641,  H6642, H6643, H6691,  H6692, H6693, H671,  H672, H673, H70001,  H70002, H70003, H70011,  H70012, H70013, H70091,  H70092, H70093, H7091,  H7092, H7093, J0101,  J0111, J0121, J0131,  J0141, J0180, J0181,  J0191, J028, J349, R0981 |
| Skin, Bones and Joints | 680.xx (carbuncle and furuncle)  681.xx (cellulitis and abscess of finger and toe)  682.xx (other cellulitis and abscess)  683.xx (acute lymphadenitis)  684.xx (impetigo)  685.xx (pilonidal cyst)  686.xx (other local infections of skin and subcutaneous tissue)  690.8x (other erythematosquamous dermatosis)  711.xx (arthropathy associated with infections)  720.81 (inflammatory spondylopathies in diseases classified elsewhere)  728.0x (infective myositis)  727.89 (other disorders of synovium, tendon, and bursa)  728.86 (necrotizing fasciitis)  730.0x (acute osteomyelitis) | A1801, A1802, B781, E832, K122, L0100, L0101, L0102, L0103, L0109, L011, L0201, L0202, L0203, L0211, L0212, L0213, L02211, L02212, L02213, L02214, L02215, L02216, L02219, L02221, L02222, L02223, L02224, L02225, L02226, L02229, L02231, L02232, L02233, L02234, L02235, L02236, L02239, L0231, L0232, L0233, L02411, L02412, L02413, L02414, L02415, L02416, L02419, L02421, L02422, L02423, L02424, L02425, L02426, L02429, L02431, L02432, L02433, L02434, L02435, L02436, L02439, L02511, L02512, L02519, L02521, L02522, L02529, L02531, L02532, L02539, L02611, L02612, L02619, L02621, L02622, L02629, L02631, L02632, L02639, L02811, L02818, L02821, L02828, L02831, L02838, L0291, L0292, L0293, L03011, L03012, L03019, L03021, L03022, L03029, L03031, L03032, L03039, L03041, L03042, L03049, L03111, L03112, L03113, L03114, L03115, L03116, L03119, L03121, L03122, L03123, L03124, L03125, L03126, L03129, L03211, L03212, L03221, L03222, L03311, L03312, L03313, L03314, L03315, L03316, L03317, L03319, L03321, L03322, L03323, L03324, L03325, L03326, L03327, L03329, L03811, L03818, L03891, L03898, L0390, L0391, L040, L041, L042, L043, L048, L049, L0501, L0502, L0591, L0592, L080, L0881, L0882, L0889, L089, L303, L88, L928, L980, L983, M0000, M00011, M00012, M00019, M00021, M00022, M00029, M00031, M00032, M00039, M00041, M00042, M00049, M00051, M00052, M00059, M00061, M00062, M00069, M00071, M00072, M00079, M0008, M0009, M0010, M00111, M00112, M00119, M00121, M00122, M00129, M00131, M00132, M00139, M00141, M00142, M00149, M00151, M00152, M00159, M00161, M00162, M00169, M00171, M00172, M00179, M0018, M0019, M0020, M00211, M00212, M00219, M00221, M00222, M00229, M00231, M00232, M00239, M00241, M00242, M00249, M00251, M00252, M00259, M00261, M00262, M00269, M00271, M00272, M00279, M0028, M0029, M0080, M00811, M00812, M00819, M00821, M00822, M00829, M00831, M00832, M00839, M00841, M00842, M00849, M00851, M00852, M00859, M00861, M00862, M00869, M00871, M00872, M00879, M0088, M0089, M009, M01X0, M01X11, M01X12, M01X19, M01X21, M01X22, M01X29, M01X31, M01X32, M01X39, M01X41, M01X42, M01X49, M01X51, M01X52, M01X59, M01X61, M01X62, M01X69, M01X71, M01X72, M01X79, M01X8, M01X9, M0210, M02111, M02112, M02119, M02121, M02122, M02129, M02131, M02132, M02139, M02141, M02142, M02149, M02151, M02152, M02159, M02161, M02162, M02169, M02171, M02172, M02179, M0218, M0219, M0230, M02311, M02312, M02319, M02321, M02322, M02329, M02331, M02332, M02339, M02341, M02342, M02349, M02351, M02352, M02359, M02361, M02362, M02369, M02371, M02372, M02379, M0238, M0239, M0280, M02811, M02812, M02819, M02821, M02822, M02829, M02831, M02832, M02839, M02841, M02842, M02849, M02851, M02852, M02859, M02861, M02862, M02869, M02871, M02872, M02879, M0288, M0289, M352, M4980, M4981, M4982, M4983, M4984, M4985, M4986, M4987, M4988, M4989, M60000, M60001, M60002, M60003, M60004, M60005, M60009, M60011, M60012, M60019, M60021, M60022, M60029, M60031, M60032, M60039, M60041, M60042, M60043, M60044, M60045, M60046, M60051, M60052, M60059, M60061, M60062, M60069, M60070, M60071, M60072, M60073, M60074, M60075, M60076, M60077, M60078, M6008, M6009, M6500, M65011, M65012, M65019, M65021, M65022, M65029, M65031, M65032, M65039, M65041, M65042, M65049, M65051, M65052, M65059, M65061, M65062, M65069, M65071, M65072, M65079, M6508, M6720, M67211, M67212, M67219, M67221, M67222, M67229, M67231, M67232, M67239, M67241, M67242, M67249, M67251, M67252, M67259, M67261, M67262, M67269, M67271, M67272, M67279, M6728, M6729, M6780, M67811, M67812, M67813, M67814, M67819, M67821, M67822, M67823, M67824, M67829, M67831, M67832, M67833, M67834, M67839, M67841, M67842, M67843, M67844, M67849, M67851, M67852, M67853, M67854, M67859, M67861, M67862, M67863, M67864, M67869, M67871, M67872, M67873, M67874, M67879, M6788, M6789, M7100, M71011, M71012, M71019, M71021, M71022, M71029, M71031, M71032, M71039, M71041, M71042, M71049, M71051, M71052, M71059, M71061, M71062, M71069, M71071, M71072, M71079, M7108, M7109, M7180, M71811, M71812, M71819, M71821, M71822, M71829, M71831, M71832, M71839, M71841, M71842, M71849, M71851, M71852, M71859, M71861, M71862, M71869, M71871, M71872, M71879, M7188, M7189, M726, M8600, M86011, M86012, M86019, M86021, M86022, M86029, M86031, M86032, M86039, M86041, M86042, M86049, M86051, M86052, M86059, M86061, M86062, M86069, M86071, M86072, M86079, M8608, M8609, M8610, M86111, M86112, M86119, M86121, M86122, M86129, M86131, M86132, M86139, M86141, M86142, M86149, M86151, M86152, M86159, M86161, M86162, M86169, M86171, M86172, M86179, M8618, M8619, M8620, M86211, M86212, M86219, M86221, M86222, M86229, M86231, M86232, M86239, M86241, M86242, M86249, M86251, M86252, M86259, M86261, M86262, M86269, M86271, M86272, M86279, M8628, M8629 |
| Hepatitis B | 070.2x (viral hepatitis B with hepatic coma)  070.3x (viral hepatitis B without mention of hepatic coma) | B160, B161, B162, B169, B180, B181, B1910, B1911 |
| Influenza | 487.xx (influenza)  488.xx (influenza due to certain identified influenza viruses) | J09019, J0902, J0903, J09090, J09098, J09119, J0912, J0913, J09190, J09198, J09X1, J09X2, J09X3, J09X9, J1008, J101, J1100, J111, J112, J1181, J1189, J129, J09010, J09018, J09091, J09092, J09110, J09118, J09191, J09192, J1000, J1001, J102, J1081, J1082, J1083, J1089, J1108, J1182, J1183 |
| Other infections | 001.xx- to 139.xx (infectious and parasitic diseases)  245.0x (acute thyroiditis)  254.1x (abscess of thymus)  360.00 (purulent endophthalmitis)  611.0x (inflammatory disease of breast) | A000, A001, A009, A0100, A0101, A0102, A0103, A0104, A0105, A0109, A011, A012, A013, A014, A020, A021, A0220, A0221, A0222, A0223, A0224, A0225, A0229, A028, A029, A030, A031, A032, A033, A038, A039, A040, A041, A042, A043, A044, A045, A046, A047, A048, A049, A050, A051, A052, A053, A054, A055, A058, A059, A060, A061, A062, A063, A064, A065, A066, A067, A0681, A0682, A0689, A069, A070, A071, A072, A073, A074, A078, A079, A080, A0811, A0819, A082, A0831, A0832, A0839, A084, A088, A09, A150, A154, A155, A156, A157, A158, A159, A170, A171, A1781, A1782, A1783, A1789, A179, A1801, A1802, A1803, A1809, A1810, A1811, A1812, A1813, A1814, A1815, A1816, A1817, A1818, A182, A1831, A1832, A1839, A184, A1850, A1851, A1852, A1853, A1854, A1859, A186, A187, A1881, A1882, A1883, A1884, A1885, A1889, A190, A191, A192, A198, A199, A200, A201, A202, A203, A207, A208, A209, A210, A211, A212, A213, A217, A218, A219, A220, A221, A222, A227, A228, A229, A230, A231, A232, A233, A238, A239, A240, A241, A242, A243, A249, A250, A251, A259, A260, A267, A268, A269, A270, A2781, A2789, A279, A280, A281, A282, A288, A289, A300, A301, A302, A303, A304, A305, A308, A309, A310, A311, A312, A318, A319, A320, A3211, A3212, A327, A3281, A3282, A3289, A329, A35, A360, A361, A362, A363, A3681, A3682, A3683, A3684, A3685, A3686, A3689, A369, A3700, A3701, A3710, A3711, A3780, A3781, A3790, A3791, A380, A381, A388, A389, A390, A391, A392, A393, A394, A3950, A3951, A3952, A3953, A3981, A3982, A3983, A3984, A3989, A399, A400, A401, A403, A408, A409, A410, A4101, A4102, A411, A412, A413, A414, A4150, A4151, A4152, A4153, A4159, A4181, A4189, A419, A420, A421, A422, A427, A4281, A4282, A4289, A429, A430, A431, A438, A439, A440, A441, A448, A449, A46, A480, A482, A483, A484, A4851, A4852, A488, A490, A4901, A4902, A491, A492, A493, A498, A499, A5001, A5002, A5003, A5004, A5005, A5006, A5007, A5008, A5009, A501, A502, A5030, A5031, A5032, A5039, A5040, A5041, A5042, A5043, A5044, A5045, A5049, A5051, A5052, A5053, A5054, A5055, A5056, A5057, A5059, A506, A507, A509, A510, A511, A512, A5131, A5132, A5139, A5141, A5142, A5143, A5144, A5145, A5146, A5149, A515, A519, A5200, A5201, A5202, A5203, A5204, A5205, A5206, A5209, A5210, A5211, A5212, A5213, A5214, A5215, A5216, A5217, A5219, A522, A523, A5271, A5272, A5273, A5274, A5275, A5276, A5277, A5278, A5279, A528, A529, A530, A539, A5400, A5401, A5402, A5403, A5409, A541, A5421, A5422, A5423, A5424, A5429, A5430, A5431, A5432, A5433, A5439, A5440, A5441, A5442, A5443, A5449, A545, A546, A5481, A5482, A5483, A5484, A5485, A5486, A5489, A549, A55, A5600, A5601, A5602, A5609, A5611, A5619, A562, A563, A564, A568, A57, A58, A5900, A5901, A5902, A5903, A5909, A598, A599, A6000, A6001, A6002, A6003, A6004, A6009, A601, A609, A630, A638, A64, A65, A660, A661, A662, A663, A664, A665, A666, A667, A668, A669, A670, A671, A672, A673, A679, A680, A681, A689, A690, A691, A6920, A6921, A6922, A6923, A6929, A698, A699, A70, A710, A711, A719, A740, A7481, A7489, A749, A750, A751, A752, A753, A759, A770, A771, A772, A773, A7740, A7741, A7749, A778, A779, A78, A790, A791, A7981, A7989, A799, A800, A801, A802, A8030, A8039, A804, A809, A8100, A8101, A8109, A811, A812, A8181, A8182, A8183, A8189, A819, A820, A821, A829, A830, A831, A832, A833, A834, A835, A836, A838, A839, A840, A841, A848, A849, A850, A851, A852, A858, A86, A870, A871, A872, A878, A879, A880, A881, A888, A89, A90, A91, A920, A921, A922, A9230, A9231, A9232, A9239, A924, A928, A929, A930, A931, A932, A938, A94, A950, A951, A959, A960, A961, A962, A968, A969, A980, A981, A982, A983, A984, A985, A988, A99, B000, B001, B002, B003, B004, B0050, B0051, B0052, B0053, B0059, B007, B0081, B0082, B0089, B009, B010, B0111, B0112, B012, B0181, B0189, B019, B020, B021, B0221, B0222, B0223, B0224, B0229, B0230, B0231, B0232, B0233, B0234, B0239, B027, B028, B029, B03, B04, B050, B051, B052, B053, B054, B0581, B0589, B059, B0600, B0601, B0602, B0609, B0681, B0682, B0689, B069, B070, B078, B079, B08010, B08011, B0802, B0803, B0804, B0809, B081, B0820, B0821, B0822, B083, B084, B085, B0860, B0861, B0862, B0869, B0870, B0871, B0872, B0879, B088, B09, B1001, B1009, B1081, B1082, B1089, B150, B159, B160, B161, B162, B169, B170, B1710, B1711, B172, B178, B179, B180, B181, B182, B188, B189, B190, B1910, B1911, B1920, B1921, B199, B20, B250, B251, B252, B258, B259, B260, B261, B262, B263, B2681, B2682, B2683, B2684, B2685, B2689, B269, B2700, B2701, B2702, B2709, B2710, B2711, B2712, B2719, B2780, B2781, B2782, B2789, B2790, B2791, B2792, B2799, B300, B301, B302, B303, B308, B309, B330, B331, B3320, B3321, B3322, B3323, B3324, B333, B334, B338, B340, B341, B342, B343, B344, B348, B350, B351, B352, B353, B354, B355, B356, B358, B359, B360, B361, B362, B363, B368, B369, B370, B371, B372, B373, B3741, B3742, B3749, B375, B376, B377, B3781, B3782, B3783, B3784, B3789, B379, B380, B381, B382, B383, B384, B387, B3881, B3889, B389, B390, B391, B392, B393, B394, B395, B399, B400, B401, B402, B403, B407, B4081, B4089, B409, B410, B417, B418, B419, B420, B421, B427, B4281, B4282, B4289, B429, B430, B431, B432, B438, B439, B440, B441, B442, B447, B4489, B449, B450, B451, B452, B453, B457, B458, B459, B460, B461, B462, B463, B464, B465, B468, B469, B470, B471, B479, B480, B481, B482, B483, B484, B488, B49, B500, B508, B509, B510, B518, B519, B520, B528, B529, B530, B531, B538, B54, B550, B551, B552, B559, B560, B561, B569, B570, B571, B572, B5730, B5731, B5732, B5739, B5740, B5741, B5742, B5749, B575, B5800, B5801, B5809, B581, B582, B583, B5881, B5882, B5883, B5889, B589, B59, B600, B6010, B6011, B6012, B6013, B6019, B602, B608, B64, B650, B651, B652, B653, B658, B659, B660, B661, B662, B663, B664, B665, B668, B669, B670, B671, B672, B6731, B6732, B6739, B674, B675, B6761, B6769, B677, B678, B6790, B6799, B680, B681, B689, B690, B691, B6981, B6989, B699, B700, B701, B710, B711, B718, B719, B72, B7300, B7301, B7302, B7309, B731, B740, B741, B742, B743, B744, B748, B749, B75, B760, B761, B768, B769, B770, B7781, B7789, B779, B780, B787, B789, B79, B80, B810, B811, B812, B813, B814, B818, B820, B829, B830, B831, B832, B833, B834, B838, B839, B850, B851, B852, B853, B854, B86, B870, B871, B872, B873, B874, B8781, B8782, B8789, B879, B880, B881, B882, B883, B888, B889, B89, B900, B901, B902, B908, B909, B91, B92, B940, B941, B942, B948, B949, B950, B951, B952, B953, B954, B955, B956, B9561, B9562, B957, B958, B960, B961, B962, B9620, B9621, B9622, B9623, B9629, B963, B964, B965, B966, B967, B9681, B9682, B9689, B970, B9710, B9711, B9712, B9719, B9721, B9729, B9730, B9731, B9732, B9733, B9734, B9735, B9739, B974, B975, B976, B977, B9781, B9789, B998, B999, D860, D861, D862, D863, D8681, D8682, D8683, D8684, D8685, D8686, D8687, D8689, D869, E060, E321, G02, G032, G14, H32, H44001, H44002, H44003, H44009, I32, I39, I673, J020, J0300, J0301, J17, J200, J201, J202, J203, J204, J205, J206, J207, K9081, L081, L444, L946, M0010, M00111, M00112, M00119, M00121, M00122, M00129, M00131, M00132, M00139, M00141, M00142, M00149, M00151, M00152, M00159, M00161, M00162, M00169, M00171, M00172, M00179, M0018, M0019, M0230, M02311, M02312, M02319, M02321, M02322, M02329, M02331, M02332, M02339, M02341, M02342, M02349, M02351, M02352, M02359, M02361, M02362, M02369, M02371, M02372, M02379, M0238, M0239, M352, M60009, N341, N61, NO DX, R1111, Z16 |
| Diarrhea | 787.91 (Diarrhea) | K522, K5289, R197 |
| Interstitial lung disease (ILD)/ Pneumonitis | 415.11 (iatrogenic pulmonary embolism and infarction)  486.xx (pneumonia, organism unspecified)  515.xx (postinflammatory pulmonary fibrosis), 508.8x (respiratory conditions due to other specified external agents)  508.9x (respiratory conditions due to unspecified external agent)  511.0x (pleurisy without mention of effusion or current tuberculosis)  511.1x (pleurisy with effusion, with mention of bacterial cause other than tuberculosis)  511.89 (other specified forms of effusion, except tuberculosis)  511.9x (unspecified plural effusion)  516.3x (idiopathic interstitial pneumonia)  516.8x (other specified alveolar and parietoalveolar pneumonopathies)  516.9x (unspecified alveolar and parietoalveolar pneumonopathy)  518.3x (pulmonary eosinophilia)  518.82 (other pulmonary insufficiency, not elsewhere classified) | I2690, I2699, J189, J708,  J709, J80, J82, J840,  J8409, J841, J8410,  J84111, J84112, J84113,  J84114, J84115, J84116,  J84117, J842, J848,  J8489, J849, J869,  J90, J918, J941, J942,  J948, J949, R091,  T800XXA, T800XXD,  T81718A, T81718D,  T8172XA, T8172XD,  T82817A, T82817D,  T82818A, T82818D,  J188, J702, J703, J704, J8417, J920, J929, J940 |
| Ischemic colitis | 557.xx (vascular insufficiency of intestine) within three months of a colonoscopy (CPT codes 45378-45387; ICD-9-CM procedure 45.23, 45.25) or colectomy (CPT codes 44140-44160; ICD-9-CM procedure 45.7x, 45.8x) AND none of the following alternative diagnoses:  558.9x (non-infectious gastroenteritis and colitis)  Crohn’s disease (555.xx)  Ulcerative colitis (556.xx)  Clostridium dificile enteritis (008.45) | K55% (vascular insufficiency of intestine) within three months of a colonoscopy (CPT codes 45378-45387; ICD-10-CM procedure codes: 0DJD8ZZ, 0D9EZX, 0D9E4ZX, 0D9E7ZX, 0D9F%, 0D9G%, 0D9H%, 0D9K%, 0D9L%, 0D9M%, 0D9N%, 0DB%) or colectomy (CPT codes 44140-44160; ICD-10-CM procedure 0DT%, ODB%) AND none of the following alternative diagnoses:  K52% (non-infectious gastroenteritis and colitis)  Crohn’s disease (K50%)  Ulcerative colitis (K51%)  Clostridium dificile enteritis (A04.7) |
| Nausea | 787.02 (nausea alone)  787.01 (nausea with vomiting) | R110, R112 |
| Thrombocytopenia | 287.1x (qualitative platelet defects)  287.4x (secondary thrombocytopenia)  287.5x (thrombocytopenia, unspecified)  287.3x (primary thrombocytopenia) | D473, D691, D693, D6941, D6942, D6949, D6951, D696 |
| Other venous embolism and thrombosis* | 451.xx (phlebitis and thrombophlebitis)  453.xx (other venous embolism and thrombosis) | *I82.%* |
| Acute venous embolism and thrombosis of deep vessels of lower extremity **(DVT)** | 453.4x (acute venous embolism and thrombosis of deep vessels of lower extremity) | I82409, I82401, I82402, I82403, I82419, I82429,  I82439, I824Y9, I82411, I82412, I82413, I82421, I82422, I82423, I82431,  I82432, I82433, I824Y1,  I824Y2, I824Y3,  I82449, I82499, I824Z9, I82441, I82442, I82443,  I82491, I82492, I82493,  I824Z1, I824Z2,I824Z3 |
| Chronic venous embolism and thrombosis of deep vessels of lower extremity | 453.5x (chronic venous embolism and thrombosis of deep vessels of lower extremity) | I82509, I82599, I82501,  I82502, I82503, I82591,  I82592, I82593, I82519,  I82529, I82539, I825Y9,  I82511, I82512, I82513,  I82521, I82522, I82523,  I82531, I82532, I82533,  I825Y1, I825Y2, I825Y3,  I82549, I825Z9, I82541, I82542, I82543, I825Z1, I825Z2, I825Z3 |
| Venous embolism and thrombosis of superficial vessels of lower extremity | 453.6x (venous embolism and thrombosis of superficial vessels of lower extremity) | I82811, I82812, I82813, I82819 |
| Chronic venous embolism and thrombosis of other specified vessels | 453.7x (chronic venous embolism and thrombosis of other specified vessels) | I82719, I82711, I82712,  I82713, I82729, I82721,  I82722, I82723, I82709,  I82701, I82702, I82703,  I82A29, I82A21, I82A22,  I82A23, I82B29, I82B21,  I82B22, I82B23, I82C29, I82C21, I82C22, I82C23,  I82291, I82211, I82891 |
| Acute venous embolism and thrombosis of other specified veins | 453.8x (acute venous embolism and thrombosis of other specified veins) | I82609, I82619, I82629, I82890, I82A19, I82B19, I82C19, I82210, I82601, I82602, I82603, I82611, I82612, I82613, I82621, I82622, I82623, I8290, I82A11, I82A12, I82A13, I82B11, I82B12, I82B13, I82C11, I82C12, I82C13 |
| Other venous embolism and thrombosis of unspecified site | 453.9x (other venous embolism and thrombosis of unspecified site) | I9291 |
| Embolism and thrombosis of unspecified artery | 444.9x (embolism and thrombosis of unspecified artery) | I749 |
| Cataracts and other ocular disorders | 366.xx (cataract)  368.xx (visual disturbances)  379.3 (aphakia and other disorders of the lens) | E0836, E0936, E1036, E1136, E1336, H25011, H25012, H25013, H25019, H25031, H25032, H25033, H25039, H25041, H25042, H25043, H25049, H25091, H25092, H25093, H25099, H2510, H2511, H2512, H2513, H2520, H2521, H2522, H2523, H25811, H25812, H25813, H25819, H2589, H259, H26001, H26002, H26003, H26009, H26011, H26012, H26013, H26019, H26031, H26032, H26033, H26039, H26041, H26042, H26043, H26049, H26051, H26052, H26053, H26059, H26061, H26062, H26063, H26069, H2609, H26101, H26102, H26103, H26109, H26111, H26112, H26113, H26119, H26121, H26122, H26123, H26129, H26131, H26132, H26133, H26139, H2620, H26211, H26212, H26213, H26219, H26221, H26222, H26223, H26229, H26231, H26232, H26233, H26239, H2630, H2631, H2632, H2633, H2640, H26411, H26412, H26413, H26419, H26491, H26492, H26493, H26499, H268, H269, H28, H53001, H53002, H53003, H53009, H53011, H53012, H53013, H53019, H53021, H53022, H53023, H53029, H53031, H53032, H53033, H53039, H5310, H5311, H53121, H53122, H53123, H53129, H53131, H53132, H53133, H53139, H53141, H53142, H53143, H53149, H5315, H5316, H5319, H532, H5330, H5331, H5332, H5333, H5334, H5340, H53411, H53412, H53413, H53419, H53421, H53422, H53423, H53429, H53431, H53432, H53433, H53439, H53451, H53452, H53453, H53459, H53461, H53462, H53469, H5347, H53481, H53482, H53483, H53489, H5350, H5351, H5352, H5353, H5354, H5355, H5359, H5360, H5361, H5362, H5363, H5369, H5371, H5372, H538, H539, R441, R483 |
| Stomatitis and mucositis* | 528.0x (stomatitis and mucositis) | K12, K120, K121, K122, K123, K1230, K1231, K1232, K1233, K1239 (i.e. K12.%) |
| Fever | 780.6 (fever and other physiological disturbances of temperature regulation)  780.60 (fever, unspecified)  780.61 (fever presenting with conditions classified elsewhere) | R502, R5081, R509 |
| Anorexia (decreased appetite) | 783.0x (anorexia) | R630 |
| Peripheral neuropathy | 356.8x (other specified idiopathic peripheral neuropathy)  356.4x (idiopathic progressive neuropathy)  356.9x (unspecified idiopathic peripheral neuropathy)  357.0x (acute infective polyneuritis)  357.1x (polyneuropathy in collagen vascular disease)  357.6x (polyneuropathy due to drugs)  357.7x (polyneuropathy due to other toxic agents)  357.8x (other inflammatory and toxic neuropathies)  357.9x (unspecified inflammatory and toxic neuropathies)  729.2x (neuralgia, neuritis, and radiculitis, unspecified | G603, G608, G609, G610,  G6181, G6189, G619, G620,  G622, G6281, G63, M5410,  M792, G611, G6282, G6289,  G629, G64, M0550, M05511,  M05512, M05519, M05521,  M05522, M05529, M05531,  M05532, M05539, M05541,  M05542, M05549, M05551, M05552, M05559, M05561, M05562, M05569, M05571,  M05572, M05579, M0559,  M5418 |
| Sudden cardiac death | 798.1x (instantaneous death)  798.2x (death occurring in less than 24 hours from onset of symptoms, not otherwise explained)  798.9x (unattended death)  799.9x (other unknown and unspecified cause of morbidity and mortality) | R69, R99 |
| Diabetes mellitus* | 250.xx (diabetes mellitus) | E10.%, E11.% (includes E1010, E1011, E1021, E1029, E10311, E10319, E1036, E1039, E1040, E1051, E10618,  E10620, E10621, E10622, E10628, E10630, E10638,  E10641, E10649,  E1065, E1069, E108, E109, E1100, E1101, E1121, E1129, E11311, E11319, E1136, E1139, E1140, E1151, E11618, E11620, E11621, E11622, E11628, E11630, E11638, E11641, E11649, E1165, E1169, E118, E119, E1022, E10321, E10329, E10331, E10339, E10341, E10349, E10351, E10359, E1041, E1042, E1043, E1044, E1049, E1052, E1059, E10610, E1122, E11321, E11329, E11331, E11339, E11341, E11349, E11351, E11359,  E1141, E1142, E1143, E1144, E1149,  E1152, E1159, E11610 |
| Type 2 Diabetes mellitus* | 250.x0, 250.x2 | E11.% |
| Hyperglycemia | 790.29 (other abnormal glucose) | R7309, R739 |
| Acute liver injury*^ (primary ALI algorithm) | Inpatient diagnoses of any of the following:  570 (acute necrosis of liver)  572.2 (hepatic coma)  572.4 (hepatorenal syndrome)  996.82 (Compl. liver transplant)  V42.7 (liver transplant status)  CPT: 47133 (Donor hepatectomy; cadaver)  CPT 47135 (Liver allotransplantation; orthotopic; partial or whole)  CPT: 47136 (Liver allotransplantation; heterotopic; partial or whole)  CPT: 47143 (Prep cadaver donor allotransplantation; no split)  CPT: 47144 (Prep cadaver donor allotransplantation; Trisegment split)  CPT: 47145 (Prep Cadaver donor allotransplantation; Lobe split)  CPT: 47146 (Reconstruction liver graft pre-allotransplantation cadaver or live donor; Venous anastomosis)  CPT: 47147 (Reconstruction liver graft pre-allotransplantation; Cadaver or live donor; Arterial anastomosis)  ICD-9-Px: 50.4 (Total Hepatectomy)  ICD-9-Px: 50.51 (Auxiliary Liver Transplant)  ICD-9-Px: 50.59 (Liver Transplant Nec) | Inpatient diagnoses of any of the following:  K72.00 (Acute and subacute hepatic failure without coma)  K72.01 (Acute and subacute necrosis of liver)  K76.2 (Central hemorrhagic necrosis of liver)  K72.90 (Hepatic failure, unspecified without coma)  K72.91 (Hepatic failure, unspecified with coma)  K70.41 (Alcoholic hepatic failure with coma)  K71.11 (Toxic liver disease with hepatic necrosis, with coma)  K72.11 (Chronic hepatic failure with Coma)  K76.7 (Hepatorenal syndrome)  T86.40 (Unspecified complication of liver transplant)  T86.41 (Liver transplant rejection)  T86.42 (Liver transplant failure)  T86.43 (Liver transplant infection)  T86.49 (Other complications of liver transplant)  Z48.23 (Encounter for aftercare following liver transplant)  Z94.4 (Liver transplant status)  CPT: 47133 (Donor hepatectomy; cadaver)  CPT 47135 (Liver allotransplantation; orthotopic; partial or whole)  CPT: 47136 (Liver allotransplantation; heterotopic; partial or whole)  CPT: 47143 (Prep cadaver donor allotransplantation; no split)  CPT: 47144 (Prep cadaver donor allotransplantation; Trisegment split)  CPT: 47145 (Prep Cadaver donor allotransplantation; Lobe split)  CPT: 47146 (Reconstruction liver graft pre-allotransplantation cadaver or live donor; Venous anastomosis)  CPT: 47147 (Reconstruction liver graft pre-allotransplantation; Cadaver or live donor; Arterial anastomosis)  ICD-10-Px: 0FT00ZZ (Resection of Liver, Open Approach)  ICD-10-Px: 0FT04ZZ (Resection of Liver, Percutaneous Endoscopic Approach)  ICD-10-Px: 0FY00Z0 (Transplantation of Liver, Allogenic, Open Approach)  ICD-10-Px: 0FY00Z1 (Transplantation of Liver, Syngeneic, Open Approach)  ICD-10-Px: 0FY00Z2 (Transplantation of Liver, Zooplastic, Open Approach) |
| Acute liver injury or elevation of transaminases (ALI definition 2 – the original ALI algorithm) | 570.xx (acute and subacute necrosis of the liver)  790.4x (Nonspecific elevation of levels of transaminase or lactic acid dehydrogenase [LDH]) | K72.00 (Acute and subacute hepatic failure without coma) K76.2 (Central hemorrhagic necrosis of liver), R740 (Nonspecific elevation of levels of transaminase or lactic acid dehydrogenase [LDH])  K72.01 (Acute and subacute necrosis of liver) |
| ALI definition 3 (more sensitive algorithm) | All ICD-9 codes listed in ALI definition 1 or 2 OR:  572.8 (Other sequela of chronic liver disease)  573.3 (Unspecified hepatitis)  573.8 (Other specified disorders of liver)  782.4 (Jaundice, unspecified, not of newborn) | All ICD-10 codes listed in ALI definition 1 or 2 OR:  K72.10 (Chronic hepatic failure without coma)  K71.6 (Toxic liver disease with hepatitis, not elsewhere classified)  K75.9 (Inflammatory liver disease, unspecified)  K76.1 (Chronic passive congestion of liver)  K76.89 (Other specified diseases of liver)  R17 (Unspecified jaundice) |
| ALI definition 4 (more specific algorithm) | Principal inpatient diagnosis of either acute hepatic necrosis (ICD-9: 570; ICD-10: K72.00, K76.2, K72.01) OR hepatorenal syndrome (ICD-9: 572.4; ICD-10: K76.7) AND at least one other ICD-9/ICD-10 acute liver injury code from Algorithm 1. | Principal inpatient diagnosis of either acute hepatic necrosis (ICD-9: 570; ICD-10: K72.00, K76.2, K72.01) OR hepatorenal syndrome (ICD-9: 572.4; ICD-10: K76.7) AND at least one other ICD-9/ICD-10 acute liver injury code from Algorithm 1. |
|  | | |
| **(Second primary cancers)** |  |  |
| Melanoma | 172.xx (malignant melanoma of skin) | C43% |
| Colon | 153.xx (malignant neoplasm of colon)  154.xx (malignant neoplasm of rectum rectosigmoid junction and anus)  235.2x (neoplasm of uncertain behavior of stomach, intestines, and rectum) | C18%, C19%, C20% |
| Gynecologic | 183.xx (malignant neoplasm of ovary and other uterine adnexa)  236.2x (neoplasm of uncertain behavior of ovary)  182.xx (malignant neoplasm of body of uterus)  179.xx (malignant neoplasm of uterus, part unspecified)  180.xx (malignant neoplasm of cervix uteri)  184.xx (malignant neoplasm of other and unspecified female genital organs)  236.1x (neoplasm of uncertain behavior of placenta) | C56%, CD391%, C54%, C55%, C53%, C57%, C58%, |
| Non melanomatous skin cancer | 173.xx (other and unspecified malignant neoplasm of skin)  238.2x (neoplasm of uncertain behavior of skin) | C44%, D485% |
| Urinary Tract | 188.xx (malignant neoplasm of bladder)  189.xx (malignant neoplasm of kidney and other and unspecified urinary organs)  236.7x (neoplasm of uncertain behavior of bladder)  236.91 (neoplasm of uncertain behavior of kidney and ureter)  239.4x (neoplasm of unspecified nature of bladder)  239.5x (neoplasm of unspecified nature of other genitourinary organs) | C67%, C64%, D41%, D494%, D495% |
| Head/neck | 140.xx to 149.9x (malignant neoplasm of lip, oral cavity, and pharynx)  160.xx (malignant neoplasm of nasal cavities middle ear and accessory sinuses)  161.xx (malignant neoplasm of larynx)  162.xx (malignant neoplasm of trachea bronchus and lung)  195.0x (malignant neoplasm of head, face, neck) | C00% to C14%, C30% to C34% |
| Lung | 162.xx (malignant neoplasm of trachea bronchus and lung)  235.9 (neoplasm of uncertain behavior of other and unspecified respiratory organs)  239.1x (neoplasm of unspecified nature of respiratory system) | C34%, D385%, D386% |
| Non colon gastrointestinal (GI) | 150.xx (malignant neoplasm of esophagus)  151.xx (malignant neoplasm of stomach)  152.xx (malignant neoplasm of small intestine including duodenum)  155.xx (malignant neoplasm of liver and intrahepatic bile ducts)  156.xx (malignant neoplasm of gallbladder and extrahepatic bile ducts)  157.xx (malignant neoplasm of pancreas)  158.xx (malignant neoplasm of retroperitoneum and peritoneum)  159.xx (malignant neoplasm of other and ill-defined sites within the digestive organs and peritoneum)  235.xx (neoplasm of uncertain behavior of digestive and respiratory organs)  235.0x (neoplasm of uncertain behavior of major salivary glands)  235.1x (neoplasm of uncertain behavior of lip, oral cavity, and pharynx)  235.2x (neoplasm of uncertain behavior of stomach, intestines, and rectum)  235.3x (neoplasm of uncertain behavior of liver and biliary passages)  239.0x (neoplasm of unspecified nature of digestive system) | C15%, C16%, C17%, C22%, C23%, C25%, C48%, C26%, D370%, D37%, D38%, D490% |
| Brain | 190.xx (malignant neoplasm of eye)  191.xx (malignant neoplasm of brain)  192.xx (malignant neoplasm of other and unspecified parts of nervous system)  237.5x (neoplasm of uncertain behavior of brain and spinal cord)  237.6x (neoplasm of uncertain behavior of meninges)  239.6x (neoplasm of unspecified nature of brain) | C68%, C70%, C71%, C72%, D43%, D42%, D496% |
| Bone/Soft tissue | 170.xx (malignant neoplasm of bone and articular cartilage)  171.xx (malignant neoplasm of connective and other soft tissue)  238.1x (neoplasm of uncertain behavior of connective and other soft tissue)  238.2x (neoplasm of uncertain behavior of skin) | C40%, C47%, C49%, D48% |
| Endocrine | 193.xx (malignant neoplasm of thyroid gland)  194.xx (malignant neoplasm of other endocrine glands and related structures)  237.0x (neoplasm of uncertain behavior of pituitary gland and craniopharyngeal duct)  237.4x (neoplasm of uncertain behavior of other and unspecified endocrine glands)  239.7x (neoplasm of unspecified nature of endocrine glands and other parts of nervous system) | C73%, C74%, C75%, D44%, D497% |
| Pleura/mediastinum | 163.xx (malignant neoplasm of pleura)  164.xx (malignant neoplasm of thymus heart and mediastinum) | C37, C38% |
| Non-specific site | 195.xx (malignant neoplasm of other and ill-defined sites)  199.xx (malignant neoplasm without specification of site)  238.8x (neoplasm of uncertain behavior of other specified sites)  238.9x (neoplasm of uncertain behavior, site unspecified)  239.8x (neoplasm of unspecified nature of other specified sites)  239.9x (neoplasm of unspecified nature, site unspecified) | C76%, C80%, D487, D489, D498%, D499% |
| Abbreviations: HIRD, HealthCore Integrated Research Database; ICD-9-CM, International Classification of Diseases, Ninth Revision, Clinical Modification; ICD-10, International Classification of Diseases, Tenth Revision. | | |
| *****All events were identified based on at least one inpatient hospitalization with the diagnosis code of interest OR two outpatient visits (including emergency department visits) on separate dates with one of the diagnosis codes of interest. The event date was defined as the second service date for a qualifying event for those requiring two visits. | | |
| ^Defined based on algorithm defined in Lo Re V, Carbonari DM, Saine ME, *et al*. Postauthorization safety study of the DPP-4 inhibitor saxagliptin: a large-scale multinational family of cohort studies of five outcomes. *BMJ Open Diab Res Care* 2017;5:e000400. doi:10.1136/bmjdrc-2017-000400 | | |

# **Supplemental Table 2:** Formation of Study Cohorts

|  | **Total** | |
| --- | --- | --- |
|  | **N** | **%** |
| **Palbociclib User Groups** |  |  |
| Received palbociclib during the study period (01 Feb 2015 to 30 Sept 2017) | 2,795 | 100.00% |
| ≥18 years of age | 2,795 | 100.00% |
| ≥=3 months of health plan coverage | 2,445 | 87.48% |
| ≥3 months with no dispensing of palbociclib or CDK4/6 inhibitor before first dispensing of palbociclib | 2,445 | 87.48% |
| New users of palbociclib-fulvestrant | 566 | 23.15% |
| New users of palbociclib-letrozole | 1,159 | 47.40% |
| All other new users of palbociclib | 720 | 29.65% |
| **Historical Comparator group** |  |  |
| Received fulvestrant during the study period (01 Jan 2011 to 31 Jan 2015) | 3,315 | 100.00% |
| ≥18 years of age | 3,315 | 100.00% |
| ≥3 months of health plan coverage | 2,773 | 83.65% |
| ≥3 months with no dispensing of fulvestrant before first dispensing of fulvestrant | 2,316 | 69.86% |
| All propensity score matched patients with fulvestrant | 561 | 16.92% |
| Abbreviation: CDK, cyclin-dependent kinase, Feb, February; Jan, January; Sept, September. | | |

# **Supplemental Table 3:** Incidence of Safety Events in New Users of Palbociclib, Overall and by Subcohort

| **Event** | **All new users of palbociclib (N=2,445)** | | | **New users of palbociclib-fulvestrant (n=566)** | | | **New users of palbociclib-letrozole (n=1,159)** | | | **All other new users of palbociclib(n=720)** | | | |
| --- | --- | --- | --- | --- | --- | --- | --- | --- | --- | --- | --- | --- | --- |
|  | **IR (per 100 person-years)** | | | **IR (per 100 person-years)** | | | **IR (per 100 person-years)** | | | **IR (per 100 person-years)** | | | |
|  | **IR** | **95% Lower CI** | **95% Upper CI** | **IR** | **95% Lower CI** | **95% Upper CI** | **IR** | **95% Lower CI** | **95% Upper CI** | **IR** | **95% Lower CI** | **95% Upper CI** |  |
| Neutropenia (sensitive)^#^ | **35.0** | **31.9** | **38.3** | 37.5 | 30.8 | 45.4 | 33.9 | 29.7 | 38.4 | 35.1 | 29.3 | 41.8 |  |
| Neutropenia (specific)^#^ | **22.1** | **19.7** | **24.7** | 23.8 | 18.6 | 30.1 | 20.7 | 17.6 | 24.2 | 23.5 | 18.9 | 29.0 |  |
| Febrile neutropenia (sensitive)^#^ | **2.9** | **2.1** | **3.9** | 3.4 | 1.7 | 6.1 | 2.5 | 1.5 | 3.8 | 3.3 | 1.7 | 5.6 |  |
| Febrile neutropenia (specific)^#^ | **0.7** | **0.4** | **1.3** | 0.9 | 0.2 | 2.7 | 0.6 | 0.2 | 1.4 | 0.7 | 0.2 | 2.2 |  |
| Leukopenia (sensitive)^#^ | **6.6** | **5.4** | **8.1** | 5.3 | 3.1 | 8.6 | 7.1 | 5.4 | 9.3 | 6.6 | 4.3 | 9.7 |  |
| Leukopenia (specific)^#^ | **6.6** | **5.3** | **8.0** | 5.3 | 3.1 | 8.6 | 7.0 | 5.3 | 9.1 | 6.6 | 4.3 | 9.7 |  |
| Alopecia | **0.8** | **0.4** | **1.4** | 0.3 | 0.0 | 1.7 | 1.0 | 0.4 | 2.0 | 0.7 | 0.2 | 2.2 |  |
| Vomiting* | **8.7** | **7.2** | **10.3** | 10.4 | 7.1 | 14.6 | 6.1 | 4.5 | 8.1 | 12.4 | 9.2 | 16.4 |  |
| QT prolongation | **5.3** | **4.2** | **6.6** | 6.3 | 3.9 | 9.8 | 4.6 | 3.3 | 6.4 | 5.8 | 3.7 | 8.7 |  |
| Fatigue* | **17.3** | **15.1** | **19.7** | 18.7 | 14.0 | 24.5 | 14.2 | 11.5 | 17.3 | 22.3 | 17.6 | 27.8 |  |
| Serious infection | **19.9** | **17.7** | **22.3** | 25.6 | 20.2 | 31.9 | 17.5 | 14.6 | 20.7 | 20.3 | 16.1 | 25.3 |  |
| Brain/spinal infection | **0.6** | **0.3** | **1.2** | 1.2 | 0.3 | 3.2 | 0.7 | 0.3 | 1.6 | 0.0 | 0.0 | 0.0 |  |
| Pericardial/myocardial infection | **0.0** | **0.0** | **0.0** | 0.0 | 0.0 | 0.0 | 0.0 | 0.0 | 0.0 | 0.0 | 0.0 | 0.0 |  |
| Pulmonary infection | **9.1** | **7.6** | **10.8** | 10.7 | 7.4 | 14.9 | 8.8 | 6.9 | 11.2 | 8.3 | 5.7 | 11.7 |  |
| GI infection | **0.8** | **0.4** | **1.4** | 0.6 | 0.1 | 2.2 | 0.7 | 0.3 | 1.6 | 1.0 | 0.3 | 2.5 |  |
| Genitourinary/renal infection | **9.4** | **7.9** | **11.0** | 11.2 | 7.8 | 15.5 | 9.3 | 7.3 | 11.7 | 8.1 | 5.5 | 11.4 |  |
| Dental infection | **0.1** | **0.0** | **0.4** | 0.0 | 0.0 | 0.0 | 0.1 | 0.0 | 0.7 | 0.0 | 0.0 | 0.0 |  |
| Ear, nose, and throat infection | **5.5** | **4.4** | **6.9** | 4.4 | 2.4 | 7.3 | 6.5 | 4.8 | 8.6 | 4.6 | 2.7 | 7.2 |  |
| Skin, bones, and joint infection | **5.4** | **4.3** | **6.7** | 5.3 | 3.1 | 8.5 | 5.8 | 4.3 | 7.7 | 4.8 | 2.9 | 7.4 |  |
| Hepatitis B infection | **0.2** | **0.0** | **0.6** | 0.0 | 0.0 | 0.0 | 0.2 | 0.0 | 0.9 | 0.2 | 0.0 | 1.4 |  |
| Influenza infection | **0.4** | **0.1** | **0.9** | 1.2 | 0.3 | 3.2 | 0.0 | 0.0 | 0.0 | 0.5 | 0.1 | 1.8 |  |
| Other infection | **19.7** | **17.4** | **22.1** | 21.2 | 16.4 | 27.1 | 19.3 | 16.3 | 22.7 | 19.1 | 15.0 | 24.0 |  |
| Diarrhea* | **4.1** | **3.1** | **5.2** | 4.4 | 2.4 | 7.4 | 3.4 | 2.3 | 5.0 | 5.1 | 3.1 | 7.9 |  |
| Interstitial lung disease/pneumonitis | **24.5** | **22.0** | **27.2** | 34.0 | 27.8 | 41.2 | 22.3 | 19.0 | 25.9 | 21.3 | 17.0 | 26.5 |  |
| Anemia (sensitive)^#^ | **39.5** | **36.2** | **43.0** | 47.4 | 39.7 | 56.1 | 39.1 | 34.6 | 44.0 | 34.1 | 28.4 | 40.6 |  |
| Anemia (specific)^#^ | **22.0** | **19.6** | **24.5** | 26.1 | 20.7 | 32.5 | 21.1 | 18.0 | 24.7 | 20.3 | 16.1 | 25.4 |  |
| Nausea* | **14.2** | **12.3** | **16.3** | 16.5 | 12.2 | 21.8 | 10.8 | 8.6 | 13.5 | 19.1 | 14.9 | 24.1 |  |
| Thrombocytopenia | **10.0** | **8.4** | **11.7** | 10.4 | 7.2 | 14.6 | 8.5 | 6.6 | 10.8 | 12.6 | 9.3 | 16.7 |  |
| Pulmonary embolism* | **3.0** | **2.2** | **4.0** | 4.4 | 2.4 | 7.4 | 2.0 | 1.2 | 3.3 | 3.8 | 2.1 | 6.2 |  |
| Pulmonary embolism (incident only) | **3.6** | **2.7** | **4.6** | 4.7 | 2.6 | 7.8 | 3.4 | 2.2 | 4.9 | 3.0 | 1.6 | 5.3 |  |
| Other venous embolism and thrombosis* | **4.3** | **3.3** | **5.5** | 3.6 | 1.8 | 6.4 | 3.6 | 2.4 | 5.2 | 6.3 | 4.1 | 9.3 |  |
| Acute venous embolism and thrombosis of deep vessels of lower extremity (DVT) | **3.0** | **2.2** | **4.0** | 2.2 | 0.9 | 4.6 | 2.1 | 1.2 | 3.4 | 5.2 | 3.3 | 8.0 |  |
| "other venous embolism and thrombosis" (incident only) | **6.2** | **5.0** | **7.6** | 7.3 | 4.6 | 11.0 | 6.3 | 4.7 | 8.3 | 5.2 | 3.3 | 8.0 |  |
| Acute venous embolism and thrombosis of deep vessels of lower extremity (DVT) | **4.0** | **3.1** | **5.2** | 5.1 | 2.9 | 8.2 | 3.5 | 2.3 | 5.1 | 4.2 | 2.5 | 6.8 |  |
| Embolism and thrombosis of unspecified artery | **0.2** | **0.0** | **0.6** | 0.3 | 0.0 | 1.7 | 0.0 | 0.0 | 0.0 | 0.5 | 0.1 | 1.8 |  |
| Cataracts and other ocular disorders | **6.3** | **5.1** | **7.7** | 6.0 | 3.6 | 9.4 | 6.9 | 5.2 | 9.0 | 5.3 | 3.3 | 8.1 |  |
| Stomatitis and mucositis | **2.3** | **1.6** | **3.2** | 2.8 | 1.3 | 5.3 | 1.4 | 0.7 | 2.4 | 3.7 | 2.1 | 6.2 |  |
| Fever | **10.1** | **8.6** | **11.9** | 11.6 | 8.2 | 16.1 | 9.8 | 7.7 | 12.2 | 9.6 | 6.8 | 13.2 |  |
| Anorexia | **2.0** | **1.3** | **2.8** | 3.1 | 1.5 | 5.7 | 1.7 | 1.0 | 2.9 | 1.5 | 0.6 | 3.2 |  |
| Peripheral neuropathy | **7.3** | **6.0** | **8.8** | 7.6 | 4.9 | 11.3 | 6.1 | 4.5 | 8.1 | 9.5 | 6.7 | 13.0 |  |
| Sudden cardiac death | **0.3** | **0.1** | **0.7** | 0.0 | 0 | 0 | 0.4 | 0.1 | 1.1 | 0.2 | 0.0 | 1.4 |  |
| Diabetes mellitus | **12.0** | **10.3** | **13.9** | 14.8 | 10.9 | 19.8 | 13.5 | 11.0 | 16.4 | 6.9 | 4.5 | 10.0 |  |
| Type 2 Diabetes mellitus | **11.9** | **10.2** | **13.9** | 14.8 | 10.9 | 19.8 | 13.5 | 11.0 | 16.4 | 6.6 | 4.3 | 9.7 |  |
| Hyperglycemia | **2.0** | **1.4** | **2.9** | 1.5 | 0.5 | 3.6 | 2.5 | 1.5 | 3.9 | 1.5 | 0.6 | 3.3 |  |
| Liver failure (Acute liver injury) | **3.1** | **2.3** | **4.1** | 4.0 | 2.1 | 6.9 | 2.7 | 1.7 | 4.1 | 3.0 | 1.5 | 5.2 |  |
| Abnormal ALT (incident)^ | **5.7** | **3.9** | **8.1** | 7.4 | 3.4 | 14.0 | 3.4 | 1.6 | 6.4 | 8.4 | 4.5 | 14.3 |  |
| Abnormal ALT (incident or prevalent)^ | **10.8** | **8.5** | **13.6** | 11.6 | 6.9 | 18.3 | 9.2 | 6.3 | 13.1 | 13.0 | 8.4 | 19.2 |  |
| Abnormal AST (incident)^ | **6.2** | **4.3** | **8.6** | 7.0 | 3.2 | 13.3 | 4.4 | 2.4 | 7.5 | 8.8 | 4.8 | 14.7 |  |
| Abnormal AST (incident or prevalent)^ | **12.1** | **9.6** | **15.0** | 13.8 | 8.5 | 21.1 | 9.1 | 6.2 | 12.9 | 16.1 | 10.9 | 22.9 |  |
| Abnormal ALP (incident)^ | **3.9** | **2.5** | **5.8** | 5.0 | 2.0 | 10.4 | 2.1 | 0.9 | 4.4 | 6.2 | 3.1 | 11.1 |  |
| Abnormal ALP (incident or prevalent)^ | **9.4** | **7.3** | **11.9** | 11.2 | 6.5 | 17.9 | 6.7 | 4.3 | 10.0 | 12.8 | 8.3 | 18.9 |  |
| Secondary malignancies (second primary cancers) | **30.0** | **27.2** | **33.1** | 36.5 | 29.9 | 44.1 | 28.7 | 24.9 | 32.9 | 27.5 | 22.4 | 33.4 |  |
| Non-melanoma skin cancer | **2.4** | **1.7** | **3.4** | 2.19 | 0.9 | 4.5 | 2.1 | 1.2 | 3.4 | 3.3 | 1.7 | 5.6 |  |
| Abbreviations: N, number; GI, gastrointestinal; PT, person-time; IR, incidence rate; CI, confidence interval; DVT, deep vein thrombosis; ALT, alanine transaminase; AST, aspartate transaminase; ALP, alkaline phosphatase. | | | | | | | | | | | | | |
| ^#^Select safety events were evaluated using a “sensitive” algorithm that prioritized minimizing false negative error, and a “specific” algorithm that prioritized minimizing false positive error. | | | | | | | | | | | | | |
| *Allowed history of these events on or prior to index date. All other events excluded individuals with a history of these events prior to the index date | | | | | | | | | | | | | |
| ^ The ALT, AST, and ALP measures were estimated using electronic outpatient laboratory data. This data was available on ~1/3 of our study population, and does not include any results for ER or inpatient settings. We classified the lab outcomes as follows: Abnormal AST > 40 U/L; Abnormal ALT > 40 U/L; Abnormal ALP > 147 U/L; incident analyses required a normal lab value prior to the index date, and an abnormal value after the index date. "Prevalent and Incident" analyses only required an abnormal value after the index date and included individuals who did not have a lab value prior to the index date and individuals who had abnormal values prior to the index date. | | | | | | | | | | | | | |

# **Supplemental Table 4:** Characteristics of New Users of Palbociclib and Fulvestrant and New Users of Fulvestrant Monotherapy (Historical Comparator Group) Before and After Propensity Score Matching (All evaluated characteristics)

| **Characteristics*** | **Before Propensity Score Matching** | | | | | **After Propensity Score Matching^** | | | | |
| --- | --- | --- | --- | --- | --- | --- | --- | --- | --- | --- |
|  | **New users of palbociclib-fulvestrant** | | **New users of fulvestrant monotherapy (pre-2015)** | | **Standardized difference** | **New users of palbociclib-fulvestrant** | | **New users of fulvestrant monotherapy (pre-2015)** | | **Standardized difference** |
|  |  |  |  |  |  |  |  |  |  |  |
|  | **N/Mean** | **%/SD** | **N/Mean** | **%/SD** |  | **N/Mean** | **%/SD** | **N/Mean** | **%/SD** |  |
| **Overall** | **566** | **100%** | **2,316** | **100%** |  | **561** | **100%** | **561** | **100%** |  |
|  |  |  |  |  |  |  |  |  |  |  |
| **Demographics** | | | | | | | | | | |
| Age at index date (in years) | 59.3 | 11.0 | 64.1 | 12.9 | 0.4 | 59.5 | 11.0 | 59.9 | 13.3 | 0.04 |
| Age (years) |  |  |  |  |  |  |  |  |  |  |
| <45 | 51.0 | 9.0 | 127.0 | 5.5 | 0.1 | 49.0 | 8.7 | 63.0 | 11.2 | 0.08 |
| 45-64 | 362.0 | 64.0 | 1121.0 | 48.4 | 0.3 | 360.0 | 64.2 | 306.0 | 54.5 | 0.20 |
| 65+ | 153.0 | 27.0 | 1068.0 | 46.1 | 0.4 | 152.0 | 27.1 | 192.0 | 34.2 | 0.16 |
| Sex |  |  |  |  |  |  |  |  |  |  |
| Male | ≤10 | n/a | 30 | n/a | 0.0 | ≤10 | n/a | ≤10 | n/a | 0.10 |
| Female | 557 | 98.4 | 2286 | 98.7 | 0.0 | 552 | 98.4 | 558 | 99.5 | 0.10 |
| Calendar year of index date |  |  |  |  |  |  |  |  |  |  |
| 2011 | 0 | 0.00 | 612 | 26.4 | n/a | 0 | 0.00 | 103 | 18.4 | n/a |
| 2012 | 0 | 0.00 | 507 | 21.9 | n/a | 0 | 0.00 | 138 | 24.6 | n/a |
| 2013 | 0 | 0.00 | 569 | 24.6 | n/a | 0 | 0.00 | 159 | 28.3 | n/a |
| 2014 | 0 | 0.00 | 628 | 27.1 | n/a | 0 | 0.00 | 161 | 28.7 | n/a |
| 2015 | 99 | 17.5 | 0 | 0.00 | n/a | 98 | 17.5 | 0 | 0.00 | n/a |
| 2016 | 269 | 47.5 | 0 | 0.00 | n/a | 267 | 47.6 | 0 | 0.00 | n/a |
| 2017 | 198 | 35.0 | 0 | 0.00 | n/a | 196 | 34.9 | 0 | 0.00 | n/a |
| Geographic region of residence |  |  |  |  |  |  |  |  |  |  |
| Midwest | 95 | 16.8 | 422 | 18.2 | 0.04 | 95 | 16.9 | 93 | 16.6 | 0.01 |
| South | 159 | 28.1 | 690 | 29.8 | 0.04 | 157 | 28.0 | 165 | 29.4 | 0.03 |
| Northeast | 166 | 29.3 | 580 | 25.0 | 0.10 | 164 | 29.2 | 158 | 28.2 | 0.02 |
| West | 146 | 25.8 | 624 | 26.9 | 0.03 | 145 | 25.8 | 145 | 25.8 | 0.00 |
| **Medical History** | | | | | | | | | | |
| Other primary cancer prior to first breast cancer diagnosis code | 223 | 39.4 | 1026 | 44.3 | 0.1 | 222 | 39.6 | 271 | 48.3 | 0.18 |
| Secondary malignancy (metastasis) | 496 | 87.6 | 1823 | 78.7 | 0.2 | 491 | 87.5 | 488 | 87.0 | 0.02 |
| Lymph nodes of head, face, and neck | 154 | 27.2 | 476 | 20.6 | 0.2 | 151 | 26.9 | 150 | 26.7 | 0.00 |
| Respiratory and digestive systems | 257 | 45.4 | 781 | 33.7 | 0.2 | 254 | 45.3 | 252 | 44.9 | 0.01 |
| Other specified sites | 463 | 81.8 | 1710 | 73.8 | 0.2 | 458 | 81.6 | 458 | 81.6 | 0.00 |
| Deyo-Charlson comorbidity index (DCI) without cancer codes | 8.5 | 1.8 | 7.85 | 2.3 | 0.3 | 8.5 | 1.8 | 8.56 | 1.6 | 0.03 |
| Secondary malignant neoplasm of breast | 66 | 11.7 | 204 | 8.8 | 0.1 | 65 | 11.6 | 67 | 11.9 | 0.01 |
| Breast cancer (female) diagnosis code | 558 | 98.6 | 2262 | 97.7 | 0.1 | 553 | 98.6 | 556 | 99.1 | 0.05 |
| InSitu breast cancer | 44 | 7.8 | 151 | 6.5 | 0.0 | 42 | 7.5 | 39 | 7.0 | 0.02 |
| **Cancer Therapy History** | | | | | | | | | | |
| **Radiation therapy** | 112 | 19.8 | 386 | 16.7 | 0.1 | 112 | 20.0 | 117 | 20.9 | 0.02 |
| External Beam | 112 | 19.8 | 386 | 16.7 | 0.1 | 112 | 20.0 | 117 | 20.9 | 0.02 |
| **Surgery** | 11 | 1.9 | 68 | 2.9 | 0.1 | 11 | 2.0 | 13 | 2.3 | 0.02 |
| Mastectomy in the last six months | ≤10 | n/a | 32 | 1.4 | 0.1 | ≤10 | n/a | ≤10 | n/a | 0.06 |
| Lumpectomy in the last six months | ≤10 | n/a | 25 | 1.1 | 0.1 | ≤10 | n/a | ≤10 | n/a | 0.04 |
| Radical mastectomy in the last six months | ≤10 | n/a | 22 | 0.9 | 0.0 | ≤10 | n/a | ≤10 | n/a | 0.02 |
| **Chemotherapy** | 100 | 17.7 | 429 | 18.5 | 0.0 | 99 | 17.6 | 98 | 17.5 | 0.00 |
| Infusion based chemo (procedure) | ≤10 | n/a | 31 | 1.3 | 0.1 | ≤10 | n/a | 12 | 2.1 | 0.16 |
| **Imaging** |  |  |  |  |  |  |  |  |  |  |
| CT related imaging in the last six months | 139 | 24.6 | 501 | 21.6 | 0.1 | 138 | 24.6 | 146 | 26.0 | 0.03 |
| MR related imaging for needle placement | 0 | 0.0 | ≤10 | n/a | --- | 0 | 0.0 | ≤10 | n/a | --- |
| Diagnostic imaging in the last six months | 72 | 12.7 | 461 | 19.9 | 0.2 | 72 | 12.8 | 79 | 14.1 | 0.04 |
| Mammography | ≤10 | n/a | 107 | 4.6 | 0.2 | ≤10 | n/a | ≤10 | n/a | 0.03 |
| MRI related imaging | 25 | 4.4 | 91 | 3.9 | 0.0 | 25 | 4.5 | 26 | 4.6 | 0.01 |
| Tomosynthesis (3D mammography) | 0 | 0.0 | 0 | 0.0 | n/a | 0 | 0.0 | 0 | 0.0 | n/a |
| **Healthcare Utilization (six months prior to index date)** | | | | | | | | | | |
| Number of outpatient visits | 37.6 | 21.7 | 36.5 | 24.6 | 0.0 | 37.7 | 21.8 | 39.3 | 24.2 | 0.07 |
| Number of outpatient visits to an oncologist | 0.0 | 0.2 | 0.0 | 0.3 | 0.0 | 0.0 | 0.2 | 0.0 | 0.3 | 0.08 |
| Number of inpatient hospitalizations | 0.3 | 0.7 | 0.3 | 0.7 | 0.0 | 0.3 | 0.7 | 0.4 | 0.8 | 0.14 |
| Number of inpatient hospitalizations for breast cancer | 0.0 | 0.2 | 0.3 | 0.6 | 0.5 | 0.0 | 0.2 | 0.3 | 0.7 | 0.58 |
| Number of inpatient hospitalizations for any cancer | 0.3 | 0.6 | 0.3 | 0.7 | 0.1 | 0.3 | 0.6 | 0.4 | 0.7 | 0.15 |
| Number of emergency department visits | 0.3 | 0.8 | 0.2 | 0.6 | 0.1 | 0.3 | 0.8 | 0.3 | 0.7 | 0.03 |
| **Medication Use (breast cancer related)** | | | | | | | | | | |
| Palbociclib | 0 | 0.00 | 0 | 0.00 | n/a | 0 | 0.00 | 0 | 0.00 | n/a |
| Aromatase inhibitor | 326 | 57.6 | 1285 | 55.5 | 0.04 | 321 | 57.2 | 303 | 54.0 | 0.06 |
| Letrozole | 116 | 20.5 | 429 | 18.5 | 0.05 | 113 | 20.1 | 116 | 20.7 | 0.01 |
| Anastrazole | 136 | 24.0 | 611 | 26.4 | 0.05 | 135 | 24.1 | 138 | 24.6 | 0.01 |
| Exemestane | 86 | 15.2 | 358 | 15.5 | 0.01 | 85 | 15.2 | 76 | 13.5 | 0.05 |
| HER2 positive therapy | 15 | 2.7 | 127 | 5.5 | 0.14 | 15 | 2.7 | 14 | 2.5 | 0.01 |
| Trastuzumab | 25 | 4.4 | 159 | 6.9 | 0.11 | 24 | 4.3 | 27 | 4.8 | 0.03 |
| Lapatinib | ≤10 | n/a | 16 | 0.7 | 0.05 | ≤10 | n/a | ≤10 | n/a | 0.03 |
| Ado-trastuzumab | 0 | 0.0 | 0 | 0.0 | n/a | 0 | 0.0 | 0 | 0.0 | n/a |
| Pertuzumab | 0 | 0.0 | 0 | 0.0 | n/a | 0 | 0.0 | 0 | 0.0 | n/a |
| Tamoxifen | 140 | 24.7 | 379 | 16.4 | 0.21 | 140 | 25.0 | 135 | 24.1 | 0.02 |
| Fulvestrant | 238 | 42.0 | 0 | 0.0 | n/a | 236 | 42.1 | 0 | 0.0 | n/a |
| Denosumab or Pamidronate | 206 | 36.4 | 424 | 18.3 | 0.41 | 201 | 35.8 | 197 | 35.1 | 0.01 |
| Everolimus | 40 | 7.1 | 84 | 3.6 | 0.15 | 39 | 7.0 | 37 | 6.6 | 0.01 |
| **Medication Use (not breast cancer related)** | | | | | | | | | | |
| Anticonvulsants | 126 | 22.3 | 347 | 15.0 | 0.19 | 122 | 21.7 | 123 | 21.9 | 0.00 |
| Antidepressants | 178 | 31.4 | 631 | 27.2 | 0.09 | 175 | 31.2 | 184 | 32.8 | 0.03 |
| Antidiabetics | 67 | 11.8 | 252 | 10.9 | 0.03 | 66 | 11.8 | 75 | 13.4 | 0.05 |
| Antifungals | 28 | 4.9 | 119 | 5.1 | 0.01 | 28 | 5.0 | 35 | 6.2 | 0.05 |
| Antihypertensives | 173 | 30.6 | 623 | 26.9 | 0.08 | 171 | 30.5 | 135 | 24.1 | 0.14 |
| Antimycobacterials | 109 | 19.3 | 446 | 19.3 | 0.00 | 107 | 19.1 | 117 | 20.9 | 0.04 |
| Antivirals | 32 | 5.7 | 112 | 4.8 | 0.04 | 32 | 5.7 | 30 | 5.3 | 0.02 |
| Corticosteroids | 148 | 26.1 | 416 | 18.0 | 0.20 | 146 | 26.0 | 146 | 26.0 | 0.00 |
| Oral contraceptive use (progestin) | 0 | 0.0 | ≤10 | n/a | n/a | 0 | 0.0 | 0 | 0.0 | n/a |
| Oral contraceptive use (combination) | ≤10 | n/a | ≤10 | n/a | 0.02 | ≤10 | n/a | ≤10 | n/a | 0.00 |
| Oral contraceptive use (unspecified) | ≤10 | n/a | 23 | 1.0 | 0.02 | ≤10 | n/a | ≤10 | n/a | 0.02 |
| Lipid lowering agent | 132 | 23.3 | 556 | 24.0 | 0.02 | 130 | 23.2 | 121 | 21.6 | 0.04 |
| Vaginal estrogen (local hormone treatment) | 0 | 0.0 | ≤10 | n/a | n/a | 0 | 0.0 | ≤10 | n/a | n/a |
| Macrolides | ≤10 | n/a | 22 | 0.9 | 0.06 | ≤10 | n/a | ≤10 | n/a | 0.08 |
| Sedatives/hypnotics | 57 | 10.1 | 291 | 12.6 | 0.08 | 57 | 10.2 | 63 | 11.2 | 0.03 |
| Selective estrogen receptor modulators | ≤10 | n/a | ≤10 | n/a | 0.03 | ≤10 | n/a | ≤10 | n/a | 0.08 |
| Unopposed estrogen hormone replacement therapy (HRT) | 0 | 0.0 | ≤10 | n/a | n/a | 0 | 0.0 | ≤10 | n/a | n/a |
| **Co-morbidities (six months prior to index date)** | | | | | | | | | | |
| Pathologic fracture | 46 | 8.1 | 173 | 7.5 | 0.02 | 46 | 8.2 | 51 | 9.1 | 0.03 |
| Osteoporosis | 56 | 9.9 | 225 | 9.7 | 0.01 | 55 | 9.8 | 52 | 9.3 | 0.02 |
| Uterine malignancies | ≤10 | n/a | 11 | 0.5 | 0.02 | ≤10 | n/a | ≤10 | n/a | 0.03 |
| Pure hypercholesterolemia | 48 | 8.5 | 237 | 10.2 | 0.06 | 48 | 8.6 | 54 | 9.6 | 0.04 |
| Major adverse cardiac events (MACE) | 20 | 3.5 | 83 | 3.6 | 0.00 | 20 | 3.6 | 23 | 4.1 | 0.03 |
| Acute myocardial infarction (MI) | ≤10 | n/a | 23 | 1.0 | 0.03 | ≤10 | n/a | ≤10 | n/a | 0.10 |
| Cerebrovascular disease | 301 | 53.2 | 1148 | 49.6 | 0.07 | 296 | 52.8 | 277 | 49.4 | 0.07 |
| Stroke | 16 | 2.8 | 63 | 2.7 | 0.01 | 16 | 2.9 | 15 | 2.7 | 0.01 |
| Hyperglycemia | 27 | 4.8 | 55 | 2.4 | 0.13 | 23 | 4.1 | 29 | 5.2 | 0.05 |
| Deyo-Charlson Index (DCI) |  |  |  |  |  |  |  |  |  |  |
| 0-3 | 19 | 3.4 | 259 | 11.2 | 0.30 | 19 | 3.4 | 17 | 3.0 | 0.02 |
| 4-7 | 12 | 2.1 | 57 | 2.5 | 0.02 | 12 | 2.1 | ≤10 | n/a | 0.12 |
| 8-11 | 512 | 90.5 | 1937 | 83.6 | 0.20 | 508 | 90.6 | 517 | 92.2 | 0.06 |
| 12 or more | 23 | 4.1 | 63 | 2.7 | 0.07 | 22 | 3.9 | 23 | 4.1 | 0.01 |
| Abbreviations: ER, estrogen receptor; HER2, human epidermal growth factor receptor 2; N, number; SD, standard deviation; CT, computed tomography; MRI, magnetic resonance imaging. | | | | | | | | | | |
| *All characteristics are measured as presence within six months prior to the index date, unless otherwise specified. | | | | | | | | | | |
| ^The following variables were included in the propensity score: age, region, Deyo-Charlson Index, number of outpatient visits, number of emergency room visits, secondary malignancy to lymph nodes of head, face, and neck, secondary malignancy to other specified sites, secondary malignancy to respiratory sites, tamoxifen, everolimus, anastrazole, denosumab or pamidronate, exemestane, chemotherapy, corticosteroids, diagnostic imaging, breast cancer surgery, letrozole, HER2 positive therapy, radiation therapy, CT imaging, mammography, MRI imaging, anticonvulsants, antidepressants, sedatives/hypnotics, secondary malignancy to breast, breast cancer diagnosis code, in situ breast cancer diagnosis, hyperglycemia, and cerebrovascular disease. | | | | | | | | | | |

# **Supplemental Table 5:** Incidence Rates and Adjusted Hazard Ratios of the Safety Events of Interest in Propensity Score Matched New Users of Palbociclib and Fulvestrant and Historical New Users of Fulvestrant Monotherapy

| **Event** |  | | | | | | | | |
| --- | --- | --- | --- | --- | --- | --- | --- | --- | --- |
|  | **New users of palbociclib-fulvestrant (n=561)** | | | **Historical new users of fulvestrant monotherapy (n=561)** | | | **Adjusted Hazard Ratios** | | |
|  | **IR (per 100 person-years)** | | | **IR (per 100 person-years)** | | | **aHR** | **95% LCL** | **95%UCL** |
|  | **IR** | **95% Lower CI** | **95% Upper CI** | **IR** | **95% Lower CI** | **95% Upper CI** |  |  |  |
| Neutropenia (sensitive) | 36.7 | 30.0 | 44.5 | 4.6 | 2.7 | 7.4 | 7.8 | 4.7 | 13.0 |
| Neutropenia (specific) | 24.0 | 18.7 | 30.3 | 3.8 | 2.1 | 6.4 | 6.3 | 3.6 | 11.2 |
| Febrile neutropenia (sensitive) | 3.1 | 1.5 | 5.8 | 0.3 | 0.0 | 1.5 | 11.1 | 1.4 | 87.1 |
| Febrile neutropenia (specific) | 0.9 | 0.2 | 2.7 | 0.0 | . | 1.0 | n/a | . | . |
| Leukopenia (sensitive) | 5.4 | 3.1 | 8.6 | 0.8 | 0.2 | 2.4 | 6.4 | 1.9 | 21.9 |
| Leukopenia (specific) | 5.4 | 3.1 | 8.6 | 0.8 | 0.2 | 2.4 | 6.4 | 1.9 | 21.9 |
| Alopecia | 0.3 | 0.0 | 1.7 | 0.0 | . | 1.0 | n/a | 0.0 | . |
| Vomiting* | 9.8 | 6.6 | 14.0 | 14.4 | 10.7 | 19.0 | 0.7 | 0.4 | 1.1 |
| QT prolongation | 6.4 | 3.9 | 9.9 | 3.5 | 1.9 | 6.0 | 1.8 | 0.9 | 3.5 |
| Fatigue* | 18.1 | 13.5 | 23.8 | 24.1 | 19.1 | 29.9 | 0.7 | 0.5 | 1.0 |
| Serious infection | 25.7 | 20.4 | 32.1 | 22.9 | 18.2 | 28.5 | 1.1 | 0.8 | 1.5 |
| Brain/spinal infection | 1.2 | 0.3 | 3.2 | 0.0 | . | 1.0 | n/a | . | . |
| Pericardial/myocardial infection | 0.0 | . | 1.2 | 0.0 | . | 1.0 | n/a | . | . |
| Pulmonary infection | 10.8 | 7.5 | 15.0 | 7.9 | 5.3 | 11.3 | 1.4 | 0.8 | 2.3 |
| GI infection | 0.6 | 0.1 | 2.3 | 0.5 | 0.1 | 1.9 | 1.1 | 0.2 | 8.1 |
| Genitourinary/Renal infection | 11.2 | 7.8 | 15.6 | 9.1 | 6.2 | 12.7 | 1.2 | 0.7 | 1.9 |
| Dental infection | 0.0 | . | 1.2 | 0.5 | 0.1 | 1.9 | n/a | . | . |
| Ear, nose, and throat infection | 4.4 | 2.4 | 7.4 | 4.9 | 2.9 | 7.8 | 0.8 | 0.4 | 1.7 |
| Skin, bones, and joint infection | 5.4 | 3.1 | 8.6 | 4.6 | 2.7 | 7.4 | 1.1 | 0.6 | 2.2 |
| Hepatitis B infection | 0.0 | . | 1.2 | 1.9 | 0.8 | 3.9 | n/a | . | . |
| Influenza infection | 1.2 | 0.3 | 3.2 | 0.5 | 0.1 | 1.9 | 2.3 | 0.4 | 12.9 |
| Other infection | 21.4 | 16.5 | 27.2 | 42.1 | 35.3 | 49.8 | 0.5 | 0.4 | 0.7 |
| Diarrhea* | 4.5 | 2.4 | 7.5 | 6.8 | 4.4 | 10.1 | 0.6 | 0.3 | 1.2 |
| Interstitial lung disease/pneumonitis | 34.2 | 27.9 | 41.5 | 24.1 | 19.2 | 29.8 | 1.4 | 1.1 | 1.9 |
| Anemia (sensitive) | 47.7 | 40.0 | 56.5 | 26.2 | 21.1 | 32.2 | 1.8 | 1.4 | 2.3 |
| Anemia (specific) | 26.3 | 20.8 | 32.8 | 9.0 | 6.2 | 12.7 | 2.9 | 1.9 | 4.3 |
| Nausea* | 16.3 | 12.0 | 21.6 | 21.0 | 16.4 | 26.5 | 0.8 | 0.5 | 1.1 |
| Thrombocytopenia | 10.5 | 7.2 | 14.7 | 4.6 | 2.7 | 7.4 | 2.3 | 1.3 | 4.1 |
| Pulmonary embolism* | 4.4 | 2.4 | 7.4 | 4.1 | 2.3 | 6.7 | 1.0 | 0.5 | 2.1 |
| No history | 4.7 | 2.7 | 7.8 | 3.2 | 1.7 | 5.7 | 1.4 | 0.7 | 3.0 |
| Other venous embolism and thrombosis* | 3.6 | 1.8 | 6.4 | 7.2 | 4.7 | 10.6 | 0.5 | 0.2 | 1.0 |
| Acute venous embolism and thrombosis of deep vessels of lower extremity (DVT) | 2.3 | 0.9 | 4.7 | 4.1 | 2.3 | 6.7 | 0.5 | 0.2 | 1.3 |
| No history of "Other venous embolism and thrombosis" | 7.0 | 4.4 | 10.6 | 6.9 | 4.5 | 10.2 | 1.0 | 0.6 | 1.8 |
| Acute venous embolism and thrombosis of deep vessels of lower extremity (DVT) | 4.8 | 2.7 | 7.9 | 4.1 | 2.3 | 6.7 | 1.2 | 0.6 | 2.4 |
| Embolism and thrombosis of unspecified artery | 0.3 | 0.0 | 1.7 | 0.3 | 0.0 | 1.5 | 1.1 | 0.1 | 17.4 |
| Cataracts and other ocular disorders | 6.0 | 3.6 | 9.5 | 7.4 | 4.9 | 10.8 | 0.8 | 0.4 | 1.4 |
| Stomatitis and mucositis | 2.8 | 1.3 | 5.3 | 0.5 | 0.1 | 1.9 | 5.0 | 1.1 | 23.1 |
| Fever | 11.4 | 8.0 | 15.8 | 6.0 | 3.8 | 9.1 | 1.9 | 1.1 | 3.2 |
| Anorexia | 3.1 | 1.5 | 5.7 | 1.3 | 0.4 | 3.1 | 2.3 | 0.8 | 6.6 |
| Peripheral neuropathy | 7.6 | 4.9 | 11.4 | 4.6 | 2.7 | 7.4 | 1.6 | 0.9 | 3.0 |
| Sudden cardiac death | 0.0 | . | 1.2 | 0.0 | . | 1.0 | n/a | . | . |
| Diabetes mellitus | 14.9 | 10.9 | 19.9 | 19.1 | 14.7 | 24.2 | 0.8 | 0.5 | 1.1 |
| Type 2 Diabetes mellitus | 14.9 | 10.9 | 19.9 | 18.7 | 14.5 | 23.9 | 0.8 | 0.5 | 1.2 |
| Hyperglycemia | 1.6 | 0.5 | 3.6 | 0.8 | 0.2 | 2.4 | 2.0 | 0.5 | 8.4 |
| Liver failure (Acute liver injury) | 4.1 | 2.2 | 6.9 | 0.8 | 0.2 | 2.4 | 4.8 | 1.4 | 16.9 |
| Abnormal ALT (incident)^ | 6.6 | 2.8 | 13.0 | 5.7 | 2.3 | 11.8 | 1.0 | 0.4 | 2.8 |
| Abnormal ALT (incident or prevalent)^ | 11.0 | 6.4 | 17.6 | 11.2 | 6.4 | 18.3 | 0.8 | 0.4 | 1.6 |
| Abnormal AST (incident)^ | 7.1 | 3.2 | 13.4 | 4.9 | 1.8 | 10.6 | 1.3 | 0.5 | 3.6 |
| Abnormal AST (incident or prevalent)^ | 13.2 | 8.1 | 20.4 | 13.2 | 7.9 | 20.6 | 0.9 | 0.5 | 1.7 |
| Abnormal ALP (incident)^ | 5.1 | 2.0 | 10.4 | 2.2 | 0.5 | 6.6 | 2.0 | 0.5 | 7.6 |
| Abnormal ALP (incident or prevalent)^ | 10.6 | 6.0 | 17.2 | 11.2 | 6.4 | 18.2 | 0.7 | 0.3 | 1.5 |
| Secondary malignancies (second primary cancer) | 36.4 | 29.8 | 44.0 | 39.2 | 32.8 | 46.4 | 0.9 | 0.7 | 1.2 |
| Non-melanoma skin cancer | 2.2 | 0.9 | 4.5 | 0.8 | 0.2 | 2.4 | 3.2 | 0.8 | 13.2 |
| Abbreviations: GI, gastrointestinal; n; number; IR, incidence rate; CI, confidence interval; aHR, adjusted hazards ratio; LCL, lower confidence limit; UCL, upper confidence limit; ALT, alanine transaminase; AST, aspartate transaminase; ALP, alkaline phosphatase; DVT, deep vein thrombosis. | | | | | | | | | |
| *Allowed history of these events on or prior to index date. All other events excluded individuals with a history of these events prior to the index date. | | | | | | | | | |
| ^The ALT, AST, and ALP measures were estimated using electronic outpatient laboratory data. This data was available on ~1/3 of our study population, and does not include any results for ER or inpatient settings. We classified the lab outcomes as follows: Abnormal AST > 40 U/L; Abnormal ALT > 40 U/L; Abnormal ALP > 147 U/L. Incident analyses required a normal lab value prior to the index date, and an abnormal value after the index date. "Prevalent and Incident" analyses only required an abnormal value after the index date and included individuals who did not have a lab value prior to the index date and individuals who had abnormal values prior to the index date. | | | | | | | | | |

# **Supplemental Table 6:** Characteristics of New Users of Palbociclib and Fulvestrant and New Users of Fulvestrant Monotherapy (Historical Comparison Group) Before and After Propensity Score Matching (Including ALI Risk Factors)

|  | **Before Propensity Score Matching** | | | | | **After Propensity Score Matching^** | | | | |
| --- | --- | --- | --- | --- | --- | --- | --- | --- | --- | --- |
| **Characteristics*** | **New users of palbociclib-fulvestrant** | | **Historical new users of fulvestrant monotherapy** | | **Standardized difference** | **New users of palbociclib-fulvestrant** | | **Historical new users of fulvestrant monotherapy** | | **Standardized difference** |
|  | **N/Mean** | **%/STD** | **N/Mean** | **%/STD** |  | **N/Mean** | **%/STD** | **N/Mean** | **%/STD** |  |
| **Overall** | **566** | **100%** | **2,316** | **100%** |  | **565** | **1.00** | **565** | **1.00** |  |
|  |  |  |  |  |  |  |  |  |  |  |
| **Demographics** | | | | | | | | | | |
| Age at index date (in years) | 59.5 | 11.4 | 64.1 | 12.9 | 0.40 | 59.5 | 11.4 | 60.0 | 12.9 | 0.04 |
| Age (years) |  |  |  |  |  |  |  |  |  |  |
| <45 | 51 | 9.0 | 127 | 5.5 | 0.14 | 51 | 9.0 | 53 | 9.4 | 0.01 |
| 45-64 | 337 | 59.5 | 1121 | 48.4 | 0.32 | 336 | 59.5 | 307 | 54.3 | 0.10 |
| 65+ | 178 | 31.4 | 1068 | 46.1 | 0.40 | 178 | 31.5 | 205 | 36.3 | 0.10 |
| Gender |  |  |  |  |  |  |  |  |  |  |
| Male | ≤10 | n/a | 30 | 1.3 | 0.02 | ≤10 | n/a | ≤10 | n/a | 0.10 |
| Female | 557 | 98.4 | 2286 | 98.7 | 0.02 | 556 | 98.4 | 556 | 98.4 | 0.00 |
| Calendar year of index date |  |  |  |  |  |  |  |  |  |  |
| 2011 | 0 | 0.0 | 612 | 26.4 | n/a | 0 | 0.0 | 118 | 20.9 | n/a |
| 2012 | 0 | 0.0 | 507 | 21.9 | n/a | 0 | 0.0 | 113 | 20.0 | n/a |
| 2013 | 0 | 0.0 | 569 | 24.6 | n/a | 0 | 0.0 | 151 | 26.7 | n/a |
| 2014 | 0 | 0.0 | 628 | 27.1 | n/a | 0 | 0.0 | 183 | 32.4 | n/a |
| 2015 | 99 | 17.5 | 0 | 0.0 | n/a | 99 | 17.5 | 0 | 0.0 | n/a |
| 2016 | 269 | 47.5 | 0 | 0.0 | n/a | 269 | 47.6 | 0 | 0.0 | n/a |
| 2017 | 198 | 35.0 | 0 | 0.0 | n/a | 197 | 34.9 | 0 | 0.0 | n/a |
| Geographic region of residence |  |  |  |  |  |  |  |  |  |  |
| Midwest | 95 | 16.8 | 422 | 18.2 | 0.04 | 95 | 16.8 | 103 | 18.2 | 0.04 |
| South | 159 | 28.1 | 690 | 29.8 | 0.04 | 159 | 28.1 | 143 | 25.3 | 0.06 |
| Northeast | 166 | 29.3 | 580 | 25.0 | 0.10 | 165 | 29.2 | 154 | 27.3 | 0.04 |
| West | 146 | 25.8 | 624 | 26.9 | 0.03 | 146 | 25.8 | 165 | 29.2 | 0.08 |
| Duration of health plan enrollment prior to index date (days) | 1263.9 | 758.3 | 694.1 | 422.9 | 0.93 | 1264.2 | 759.0 | 744.6 | 444.5 | 0.84 |
| **Medical History** | | | | | | | | | | |
| Other primary cancer prior to first breast cancer diagnosis code | 223 | 39.40 | 1026 | 44.30 | 0.10 | 223 | 39.47 | 265 | 46.90 | 0.15 |
| Secondary malignancy (metastasis) |  |  |  |  |  |  |  |  |  |  |
| Lymph nodes of head, face, and neck | 154 | 27.2 | 476 | 20.6 | 0.16 | 153 | 27.1 | 160 | 28.3 | 0.03 |
| Respiratory and digestive systems | 257 | 45.4 | 781 | 33.7 | 0.24 | 257 | 45.5 | 244 | 43.2 | 0.05 |
| Other specified sites | 463 | 81.8 | 1710 | 73.8 | 0.19 | 462 | 81.8 | 458 | 81.1 | 0.02 |
| Deyo-Charlson comorbidity index (DCI) | 8.52 | 1.8 | 7.85 | 2.3 | 0.31 | 9 | 1.8 | 8.44 | 1.7 | 0.05 |
| Secondary malignant neoplasm of breast | 66 | 11.7 | 204 | 8.8 | 0.09 | 66 | 11.7 | 64 | 11.3 | 0.01 |
| Breast Cancer (Female) diagnosis code | 558 | 98.6 | 2262 | 97.7 | 0.07 | 557 | 98.6 | 556 | 98.4 | 0.01 |
| InSitu Breast Cancer | 44 | 7.8 | 151 | 6.5 | 0.05 | 43 | 7.6 | 45 | 8.0 | 0.01 |
| History of Breast Cancer | 370 | 65.4 | 1338 | 57.8 | 0.16 | 349 | 61.8 | 370 | 65.5 | 0.08 |
| **Cancer Therapy History** | | | | | | | | | | |
| **Radiation therapy** | 112 | 19.8 | 386 | 16.7 | 0.08 | 112 | 19.8 | 109 | 19.3 | 0.01 |
| Implantation (Brachytherapy) | 0 | 0.0 | ≤10 | n/a | n/a | 0 | 0.0 | 0 | 0.0 | n/a |
| External Beam | 112 | 19.8 | 386 | 16.7 | 0.08 | 112 | 19.8 | 109 | 19.3 | 0.01 |
| **Surgery** | 11 | 1.9 | 68 | 2.9 | 0.06 | 11 | 1.9 | 13 | 2.3 | 0.02 |
| Mastectomy in the last six months | ≤10 | n/a | 32 | 1.4 | 0.09 | ≤10 | n/a | ≤10 | n/a | 0.06 |
| Lumpectomy in the last six months | ≤10 | n/a | 25 | 1.1 | 0.06 | ≤10 | n/a | ≤10 | n/a | 0.04 |
| Radical Mastectomy in the last six months | ≤10 | n/a | 22 | 0.9 | 0.01 | ≤10 | n/a | ≤10 | n/a | 0.02 |
| **Chemotherapy** | 100 | 17.7 | 429 | 18.5 | 0.02 | 100 | 17.7 | 105 | 18.6 | 0.02 |
| Infusion based chemo (procedure) | ≤10 | n/a | 31 | 1.3 | 0.11 | ≤10 | n/a | 12 | 2.1 | 0.16 |
| **Imaging** |  |  |  |  |  |  |  |  |  |  |
| CT related imaging in the last six months | 139 | 24.6 | 501 | 21.6 | 0.07 | 139 | 24.6 | 132 | 23.4 | 0.03 |
| MR related imaging for needle placement | 0 | 0.0 | ≤10 | n/a | --- | 0 | 0.0 | ≤10 | n/a | --- |
| Diagnostic imaging in the last six months | 72 | 12.7 | 461 | 19.9 | 0.20 | 72 | 12.7 | 80 | 14.2 | 0.04 |
| Mammography | ≤10 | n/a | 107 | 4.6 | 0.19 | ≤10 | n/a | ≤10 | n/a | 0.03 |
| MRI related imaging | 25 | 4.4 | 91 | 3.9 | 0.02 | 25 | 4.4 | 24 | 4.2 | 0.01 |
| Tomosynthesis (3D Mammography) | 0 | 0.0 | 0 | 0.0 | n/a | 0 | 0.0 | 0 | 0.0 | n/a |
| **Healthcare Utilization (Six months prior to index date)** | | | | | | | | | | |
| Number of outpatient visits | 38.58 | 23.6 | 36.5 | 24.6 | 0.05 | 39 | 23.7 | 39.2 | 25.0 | 0.02 |
| Number of outpatient visits to an oncologist | 0.02 | 0.2 | 0.0 | 0.3 | 0.04 | 0 | 0.2 | 0.0 | 0.2 | 0.00 |
| Number of inpatient hospitalizations | 0.33 | 0.7 | 0.3 | 0.7 | 0.04 | 0 | 0.7 | 0.4 | 0.8 | 0.08 |
| Number of inpatient hospitalizations for breast cancer | 0.03 | 0.2 | 0.3 | 0.6 | 0.51 | 0 | 0.2 | 0.3 | 0.7 | 0.55 |
| Number of inpatient hospitalizations for any cancer | 0.29 | 0.6 | 0.3 | 0.7 | 0.06 | 0 | 0.6 | 0.4 | 0.7 | 0.10 |
| Number of emergency department visits | 0.31 | 0.8 | 0.2 | 0.6 | 0.11 | 0 | 0.8 | 0.3 | 0.6 | 0.09 |
| **Medication Use (breast cancer related)** | | | | | | | | | | |
| Palbociclib | 0 | 0.0 | 0 | 0.0 | n/a | 0 | 0.0 | 0 | 0.0 | n/a |
| Hormone Therapy | 326 | 57.6 | 1285 | 55.5 | 0.04 | 325 | 57.5 | 293 | 51.9 | 0.11 |
| Letrozole | 116 | 20.5 | 429 | 18.5 | 0.05 | 115 | 20.4 | 121 | 21.4 | 0.03 |
| Anastrazole | 136 | 24.0 | 611 | 26.4 | 0.05 | 136 | 24.1 | 128 | 22.7 | 0.03 |
| Exemestane | 86 | 15.2 | 358 | 15.5 | 0.01 | 86 | 15.2 | 74 | 13.1 | 0.06 |
| HER2 positive Therapy | 15 | 2.7 | 127 | 5.5 | 0.14 | 15 | 2.7 | 19 | 3.4 | 0.04 |
| Trastuzumab | 25 | 4.4 | 159 | 6.9 | 0.11 | 25 | 4.4 | 33 | 5.8 | 0.06 |
| Lapatinib | ≤10 | n/a | 16 | 0.7 | 0.05 | ≤10 | n/a | ≤10 | n/a | 0.03 |
| Ado-trastuzumab | 0 | 0.0 | 0 | 0.0 | n/a | 0 | 0.0 | 0 | 0.0 | n/a |
| Pertuzumab | 0 | 0.0 | 0 | 0.0 | n/a | 0 | 0.0 | 0 | 0.0 | n/a |
| Tamoxifen | 140 | 24.7 | 379 | 16.4 | 0.21 | 140 | 24.8 | 125 | 22.1 | 0.06 |
| Fulvestrant | 0 | 0.0 | 0 | 0.0 | n/a | 0 | 0.0 | 0 | 0.0 | n/a |
| Denosumab or Pamidronate | 206 | 36.4 | 424 | 18.3 | 0.41 | 205 | 36.3 | 178 | 31.5 | 0.10 |
| Everolimus | 40 | 7.1 | 84 | 3.6 | 0.15 | 40 | 7.1 | 33 | 5.8 | 0.05 |
| **Medication Use (not breast cancer related)** | | | | | | | | | | |
| Anticonvulsants | 126 | 22.3 | 347 | 15.0 | 0.19 | 125 | 22.1 | 115 | 20.4 | 0.04 |
| Antidepressants | 178 | 31.4 | 631 | 27.2 | 0.09 | 177 | 31.3 | 176 | 31.2 | 0.00 |
| Antidiabetics | 67 | 11.8 | 252 | 10.9 | 0.03 | 67 | 11.9 | 75 | 13.3 | 0.04 |
| Antifungals | 28 | 4.9 | 119 | 5.1 | 0.01 | 28 | 5.0 | 35 | 6.2 | 0.05 |
| Antihypertensives | 173 | 30.6 | 623 | 26.9 | 0.08 | 173 | 30.6 | 179 | 31.7 | 0.02 |
| Antimycobacterials | 109 | 19.3 | 446 | 19.3 | 0.00 | 109 | 19.3 | 120 | 21.2 | 0.05 |
| Antivirals | 32 | 5.7 | 112 | 4.8 | 0.04 | 32 | 5.7 | 33 | 5.8 | 0.01 |
| Corticosteroids | 148 | 26.1 | 416 | 18.0 | 0.20 | 147 | 26.0 | 142 | 25.1 | 0.02 |
| Oral contraceptive use (progestin) | 0 | 0.0 | ≤10 | n/a | n/a | 0 | 0.0 | 0 | 0.0 | n/a |
| Oral contraceptive use (combination) | ≤10 | n/a | ≤10 | n/a | 0.02 | ≤10 | n/a | ≤10 | n/a | 0.00 |
| Oral contraceptive use (unspecified) | ≤10 | n/a | 23 | 1.0 | 0.02 | ≤10 | n/a | ≤10 | n/a | 0.02 |
| Lipid lowering agent | 132 | 23.3 | 556 | 24.0 | 0.02 | 131 | 23.2 | 137 | 24.2 | 0.02 |
| Vaginal estrogen (local hormone treatment) | 0 | 0.0 | ≤10 | n/a | n/a | 0 | 0.0 | ≤10 | n/a | n/a |
| Macrolides | ≤10 | n/a | 22 | 0.9 | 0.06 | ≤10 | n/a | ≤10 | n/a | 0.08 |
| Sedatives/hypnotics | 57 | 10.1 | 291 | 12.6 | 0.08 | 57 | 10.2 | 63 | 11.2 | 0.03 |
| Selective estrogen receptor modulators | ≤10 | n/a | ≤10 | n/a | 0.03 | ≤10 | n/a | ≤10 | n/a | 0.08 |
| Unopposed estrogen hormone replacement therapy (HRT) | 0 | 0.0 | ≤10 | n/a | n/a | 0 | 0.0 | ≤10 | n/a | n/a |
| **Co-morbidities (six months prior to index date)** | | | | | | | | | | |
| Pathologic fracture | 46 | 8.1 | 173 | 7.5 | 0.02 | 46 | 8.1 | 43 | 7.6 | 0.02 |
| Osteoporosis | 56 | 9.9 | 225 | 9.7 | 0.01 | 56 | 9.9 | 41 | 7.3 | 0.09 |
| Uterine malignancies | ≤10 | n/a | 11 | 0.5 | 0.02 | ≤10 | n/a | ≤10 | n/a | 0.03 |
| Pure hypercholesterolemia | 48 | 8.5 | 237 | 10.2 | 0.06 | 48 | 8.5 | 65 | 11.5 | 0.10 |
| Major adverse cardiac events (MACE) | 20 | 3.5 | 83 | 3.6 | 0.00 | 20 | 3.5 | 21 | 3.7 | 0.01 |
| Acute myocardial infarction (MI) | ≤10 | n/a | 23 | 1.0 | 0.03 | ≤10 | n/a | ≤10 | n/a | 0.10 |
| Cerebrovascular disease | 19 | 3.4 | 104 | 4.5 | 0.05 | 19 | 3.4 | 21 | 3.7 | 0.02 |
| Stroke | 16 | 2.8 | 63 | 2.7 | 0.01 | 16 | 2.8 | 16 | 2.8 | 0.00 |
| Diabetes | 67 | 11.8 | 252 | 10.9 | 0.03 | 67 | 11.9 | 75 | 13.3 | 0.04 |
| Hyperglycemia | 27 | 4.8 | 55 | 2.4 | 0.13 | 26 | 4.6 | 19 | 3.4 | 0.06 |
| Deyo-Charlson Index (DCI) |  |  |  |  |  |  |  |  |  |  |
| 0-3 | 19 | 3.4 | 259 | 11.2 | 0.30 | 19 | 3.4 | 23 | 4.1 | 0.04 |
| 4-7 | 12 | 2.1 | 57 | 2.5 | 0.02 | 12 | 2.1 | ≤10 | n/a | 0.12 |
| 8-11 | 512 | 90.5 | 1937 | 83.6 | 0.20 | 511 | 90.4 | 517 | 91.5 | 0.04 |
| 12 or more | 23 | 4.1 | 63 | 2.7 | 0.07 | 23 | 4.1 | 20 | 3.5 | 0.03 |
| **ALI related risk factors** | | | | | | | | | | |
| Chronic liver disease or Alcoholism | 74 | 13.1 | 246 | 10.6 | 0.09 | 74 | 13.1 | 69 | 12.2 | 0.03 |
| Chronic or acute hepatitis | ≤10 | n/a | ≤10 | n/a | 0.04 | ≤10 | n/a | ≤10 | n/a | 0.00 |
| Chronic or acute disease of gallbladder or pancreas | 36 | 6.4 | 145 | 6.3 | 0.01 | 36 | 6.4 | 34 | 6.0 | 0.01 |
| Hepatic, Biliary or pancreatic cancer | 160 | 28.3 | 458 | 19.8 | 0.18 | 160 | 28.3 | 143 | 25.3 | 0.07 |
| Congestive heart failure | 29 | 5.1 | 159 | 6.9 | 0.07 | 29 | 5.1 | 29 | 5.1 | 0.00 |
| **Medications association with liver injury** | | | | | | | | | | |
| **Any medication (of list below)** | 467 | 82.51 | 1749 | 75.52 | 0.17 | 466 | 82.48 | 466 | 82.48 | 0.00 |
| Acarbose | 0 | 0.00 | 0 | 0.00 | n/a | 0 | 0.00 | 0 | 0.00 | n/a |
| Acetaminophen (prescription) | 204 | 36.04 | 899 | 38.82 | 0.06 | 204 | 36.11 | 202 | 35.75 | 0.01 |
| Allopurinol | ≤10 | n/a | 19 | 0.82 | 0.02 | ≤10 | n/a | ≤10 | n/a | 0.02 |
| Amiodarone | ≤10 | n/a | 14 | 0.60 | 0.01 | ≤10 | n/a | ≤10 | n/a | 0.02 |
| Amitriptyline | ≤10 | n/a | 35 | 1.51 | 0.01 | ≤10 | n/a | ≤10 | n/a | 0.03 |
| Amoxicillin + clavulanic acid | 33 | 5.83 | 116 | 5.01 | 0.04 | 33 | 5.84 | 28 | 4.96 | 0.04 |
| Anabolic steroids | 0 | 0.00 | 0 | 0.00 | n/a | 0 | 0.00 | 0 | 0.00 | n/a |
| Aripiprazole | ≤10 | n/a | ≤10 | n/a | 0.07 | ≤10 | n/a | ≤10 | n/a | 0.04 |
| Azathioprine | ≤10 | n/a | ≤10 | n/a | 0.01 | ≤10 | n/a | ≤10 | n/a | 0.03 |
| Baclofen | ≤10 | n/a | 16 | 0.69 | 0.04 | ≤10 | n/a | ≤10 | n/a | 0.03 |
| Bupropion | 0 | 0.00 | 0 | 0.00 | n/a | 0 | 0.00 | 0 | 0.00 | n/a |
| Captopril | 0 | 0.00 | ≤10 | n/a | n/a | 0 | 0.00 | 0 | 0.00 | n/a |
| Carbamazepine | ≤10 | n/a | ≤10 | n/a | 0.04 | ≤10 | n/a | ≤10 | n/a | 0.00 |
| Chlorpromazine | ≤10 | n/a | ≤10 | n/a | 0.04 | 0 | 0.00 | 0 | 0.00 | n/a |
| Ciprofloxacin | 46 | 8.13 | 238 | 10.28 | 0.07 | 46 | 8.14 | 39 | 6.90 | 0.05 |
| Clindamycin | 23 | 4.06 | 74 | 3.20 | 0.06 | 23 | 4.07 | 25 | 4.42 | 0.02 |
| Clopidogrel | ≤10 | n/a | 57 | 2.46 | 0.06 | ≤10 | n/a | ≤10 | n/a | 0.01 |
| Cyproheptadine | ≤10 | n/a | ≤10 | n/a | 0.05 | 0 | 0.00 | 0 | 0.00 | n/a |
| Duloxetine | 23 | 4.06 | 66 | 2.85 | 0.06 | 23 | 4.07 | 24 | 4.25 | 0.01 |
| Enalapril | ≤10 | n/a | 33 | 1.42 | 0.07 | ≤10 | n/a | ≤10 | n/a | 0.07 |
| Erythromycins | 0 | 0.00 | ≤10 | n/a | n/a | 0 | 0.00 | 0 | 0.00 | n/a |
| Estrogens | 63 | 11.13 | 265 | 11.44 | 0.01 | 63 | 11.15 | 63 | 11.15 | 0.00 |
| Fluoxetine | 12 | 2.12 | 47 | 2.03 | 0.01 | 12 | 2.12 | 11 | 1.95 | 0.01 |
| Flutamide | 0 | 0.00 | 0 | 0.00 | n/a | 0 | 0.00 | 0 | 0.00 | n/a |
| HAART drugs | 0 | 0.00 | ≤10 | n/a | n/a | 0 | 0.00 | 0 | 0.00 | n/a |
| Irbesartan | ≤10 | n/a | ≤10 | n/a | 0.03 | ≤10 | n/a | ≤10 | n/a | 0.03 |
| Isoniazid | 0 | 0.00 | 0 | 0.00 | n/a | 0 | 0.00 | 0 | 0.00 | n/a |
| Ketoconazole | ≤10 | n/a | ≤10 | n/a | 0.07 | ≤10 | 0.88 | ≤10 | n/a | 0.02 |
| Lamotrigine | ≤10 | n/a | 17 | 0.73 | 0.05 | ≤10 | 0.35 | ≤10 | n/a | 0.11 |
| Lisinopril | 76 | 13.43 | 237 | 10.23 | 0.10 | 76 | 13.45 | 80 | 14.16 | 0.02 |
| Losartan | 54 | 9.54 | 121 | 5.22 | 0.17 | 54 | 9.56 | 48 | 8.50 | 0.04 |
| Methotrexate | ≤10 | n/a | 14 | 0.60 | 0.07 | ≤10 | n/a | ≤10 | n/a | 0.06 |
| Mirtazapine | ≤10 | n/a | 34 | 1.47 | 0.04 | ≤10 | n/a | ≤10 | n/a | 0.03 |
| Nitrofurantoin | ≤10 | n/a | 71 | 3.07 | 0.08 | ≤10 | n/a | 13 | 2.30 | 0.04 |
| NSAIDs | 129 | 22.79 | 398 | 17.18 | 0.13 | 128 | 22.65 | 124 | 21.95 | 0.02 |
| Omeprazole | 76 | 13.43 | 305 | 13.17 | 0.01 | 76 | 13.45 | 81 | 14.34 | 0.03 |
| Oral contraceptives | ≤10 | n/a | ≤10 | n/a | 0.02 | 0 | 0.00 | 0 | 0.00 | n/a |
| Paroxetine | ≤10 | n/a | 30 | 1.30 | 0.02 | ≤10 | n/a | ≤10 | n/a | 0.03 |
| Phenobarbital | 0 | 0.00 | ≤10 | n/a | n/a | 0 | 0.00 | 0 | 0.00 | n/a |
| Phenothiazines | 92 | 16.25 | 276 | 11.92 | 0.12 | 92 | 16.28 | 84 | 14.87 | 0.04 |
| Phenytoin | 0 | 0.00 | ≤10 | n/a | n/a | 0 | 0.00 | ≤10 | n/a | n/a |
| Pyrazinamide | 0 | 0.00 | 0 | 0.00 | n/a | 0 | 0.00 | 0 | 0.00 | n/a |
| Rifampicin | 0 | 0.00 | ≤10 | n/a | n/a | 0 | 0.00 | ≤10 | n/a | n/a |
| Risperidone | ≤10 | n/a | ≤10 | n/a | 0.02 | ≤10 | n/a | ≤10 | n/a | 0.03 |
| Sertraline | 19 | 3.36 | 72 | 3.11 | 0.01 | 19 | 3.36 | 23 | 4.07 | 0.04 |
| Statins | 125 | 22.08 | 502 | 21.68 | 0.01 | 124 | 21.95 | 121 | 21.42 | 0.01 |
| Sulfonamides | 0 | 0.00 | ≤10 | n/a | n/a | 0 | 0.00 | ≤10 | n/a | n/a |
| Terbinafine | ≤10 | n/a | ≤10 | n/a | 0.01 | ≤10 | n/a | ≤10 | n/a | 0.06 |
| Tetracyclines | 31 | 5.48 | 69 | 2.98 | 0.12 | 31 | 5.49 | 33 | 5.84 | 0.02 |
| Trazodone | 15 | 2.65 | 60 | 2.59 | 0.01 | 15 | 2.65 | 20 | 3.54 | 0.05 |
| Tricyclics | 0 | 0.00 | ≤10 | n/a | n/a | 0 | 0.00 | 0 | 0.00 | n/a |
| Trimethoprim-sulfamethoxazole | 28 | 4.95 | 123 | 5.31 | 0.00 | 28 | 4.96 | 30 | 5.31 | 0.02 |
| Trovafloxacin | 0 | 0.00 | 0 | 0.00 | n/a | 0 | 0.00 | 0 | 0.00 | n/a |
| Valproic acid | ≤10 | n/a | ≤10 | n/a | 0.04 | ≤10 | n/a | ≤10 | n/a | 0.08 |
| Verapamil | ≤10 | n/a | 22 | 0.95 | 0.05 | ≤10 | n/a | ≤10 | n/a | 0.00 |
| Abbreviations: Feb., February; ER, estrogen receptor; HER2, human epidermal growth factor receptor 2; N, number; STD, standard deviation; ALI, acute liver injury; CT, computed tomography; MRI, magnetic resonance imaging; HAART, highly active antiretroviral therapy; NSAIDs, nonsteroidal anti-inflammatory drugs. | | | | | | | | | | |
| *All characteristics are measured as presence within six months prior to the index date, unless otherwise specified. | | | | | | | | | | |
| ^The following variables were included in the propensity score: age, region, Deyo-Charlson Index, number of outpatient visits, number of emergency room visits, secondary malignancy to lymph nodes of head, face, and neck, secondary malignancy to other specified sites, secondary malignancy to respiratory sites, tamoxifen, everolimus, anastrazole, denosumab or pamidronate, exemestane, chemotherapy, corticosteroids, diagnostic imaging, breast cancer surgery, letrozole, HER2 positive therapy, radiation therapy, CT imaging, mammography, MRI imaging, anticonvulsants, antidepressants, sedatives/hypnotics, secondary malignancy to breast, breast cancer diagnosis code, in situ breast cancer diagnosis, hyperglycemia, cerebrovascular disease, Chronic liver disease or Alcoholism, Chronic or acute disease of gallbladder or pancreas, Hepatic, Biliary or pancreatic cancer, Congestive heart failure, any medication associated with ALI- Acetaminophen, Allopurinol, Amiodarone, Amitriptyline, + clavulanic acid, Aripiprazole, Baclofen, Ciprofloxacin, Clindamycin, Clopidogrel, Duloxetine, Estrogens, Fluoxetine, Ketoconazole, Lisinopril, Losartan, Mirtazapine, Nitrofurantoin, NSAIDs, Omeprazole, Paroxetine, Phenothiazine, Sertraline, Statins, Tetracycline, Trazodone, and Trimethoprim. | | | | | | | | | | |

# **Supplemental Table 7:** Unadjusted and Adjusted Hazard Ratios of ALI in New Users of Palbociclib and Fulvestrant and New Users of Fulvestrant Monotherapy (Historical Comparator)

| **Event** | **Unadjusted Hazard Ratios** | | | | | **Adjusted Hazard Ratios^** | | | | |
| --- | --- | --- | --- | --- | --- | --- | --- | --- | --- | --- |
|  | **Palbociclib -fulvestrant** | **Fulvestrant monotherapy** | **HR** | **95% LCL** | **95% UCL** | **Palbociclib-fulvestrant** | **Fulvestrant monotherapy** | **aHR** | **95% LCL** | **95% UCL** |
|  | **IR (per 100 person-years)** | |  |  |  | **IR (per 100 person-years)** | |  |  |  |
| Acute liver injury (primary algorithm*) | 4.0 | 1.3 | 2.8 | 1.4 | 5.6 | 4.0 | 1.2 | 3.0 | 1.1 | 8.4 |
| Acute liver injury - 2 (original with labs algorithm*) | 4.0 | 1.2 | 3.1 | 1.5 | 6.2 | 4.0 | 1.7 | 2.2 | 0.9 | 5.4 |
| Acute liver injury - 3 (sensitive algorithm*) | 12.3 | 2.1 | 5.4 | 3.4 | 8.5 | 12.3 | 2.5 | 4.6 | 2.3 | 9.1 |
| Acute liver injury – 4 (specific algorithm*) | -- | -- | -- | -- | -- |  |  | -- | -- | -- |
| Abbreviations: LCL, lower confidence limit; UCL, confidence limit; HR, hazard ratio; aHR, adjusted hazard ratio; IR, incidence rate. | | | | | | | | | | |
| *Algorithm definitions provided in Supplemental Table 1 | | | | | | | | | | |
| ^The propensity score included the following variables: age, region, DCI, number of outpatient visits, number of emergency room visits, secondary malignancy to lymph nodes of head, face, and neck, secondary malignancy to other specified sites, secondary malignancy to respiratory sites, tamoxifen, everolimus, anastrazole, denosumab or pamidronate, exemestane, chemotherapy, corticosteroids, diagnostic imaging, breast cancer surgery, letrozole, HER2+ therapy, radiation therapy, CT imaging, mammography, MRI imaging, anticonvulsants, antidepressants, sedatives/hypnotics, secondary malignancy to breast, breast cancer diagnosis code, in situ breast cancer diagnosis, hyperglycemia, cerebrovascular disease, chronic liver disease or Alcoholism, chronic or acute disease of gallbladder or pancreas, hepatic, biliary or pancreatic cancer, congestive heart failure, any medication associated with ALI, including acetaminophen, allopurinol, amiodarone, amitriptyline, clavulanic acid, aripiprazole, baclofen, ciprofloxacin, clindamycin, clopidogrel, duloxetine, estrogens, fluoxetine, ketoconazole, lisinopril, losartan, mirtazapine, nitrofurantoin, NSAIDs, omeprazole, paroxetine, phenothiazine, sertraline, statins, tetracycline, trazodone, and trimethoprim. | | | | | | | | | | |

# **Supplemental Table 8:** Characteristics of New Users of Palbociclib and Fulvestrant and New Users of Fulvestrant Monotherapy (Contemporaneous Comparison Group)

| **Characteristics*** | **New users of palbociclib-fulvestrant** | | **New users of fulvestrant monotherapy (Feb. 2015 or later)** | | **Standardized difference** | **New users of palbociclib-fulvestrant** | | **New users of fulvestrant monotherapy (Feb. 2015 or later)** | | **Standardized difference** |
| --- | --- | --- | --- | --- | --- | --- | --- | --- | --- | --- |
|  | **N/Mean** | **%/STD** | **N/Mean** | **%/STD** |  | **N/Mean** | **%/STD** | **N/Mean** | **%/STD** |  |
| **Overall** | **566** | **100%** | **961** | **100%** |  | **292** | **100%** | **292** | **100%** |  |
|  |  |  |  |  |  |  |  |  |  |  |
| **Demographics** | | | | | | | | | | |
| Age at index date (in years) | 59.5 | 11.4 | 66 | 13.5 | 0.52 | 64.1 | 10.8 | 65.6 | 12.7 | 0.13 |
| Age (years) |  |  |  |  |  |  |  |  |  |  |
| <45 | 51 | 9.0 | 54 | 5.6 | 0.13 | 8 | 2.7 | 16 | 5.5 | 0.14 |
| 45-64 | 337 | 59.5 | 381 | 39.6 | 0.41 | 153 | 52.4 | 110 | 37.7 | 0.30 |
| 65+ | 178 | 31.4 | 526 | 54.7 | 0.48 | 131 | 44.9 | 166 | 56.8 | 0.24 |
| Gender |  |  |  |  |  |  |  |  |  |  |
| Male | ≤10 | n/a | 15 | 1.6 | 0.00 | ≤10 | n/a | ≤10 | n/a | 0.07 |
| Female | 557 | 98.4 | 946 | 98.4 | 0.00 | 284 | 97.3 | 287 | 98.3 | 0.07 |
| Calendar year of index date |  |  |  |  |  |  |  |  |  |  |
| 2015 | 99 | 17.5 | 444 | 46.2 | 0.65 | 91 | 31.2 | 97 | 33.2 | 0.04 |
| 2016 | 269 | 47.5 | 325 | 33.8 | 0.28 | 125 | 42.8 | 114 | 39.0 | 0.08 |
| 2017 | 198 | 35.0 | 192 | 20.0 | 0.34 | 76 | 26.0 | 81 | 27.7 | 0.04 |
| Geographic region of residence |  |  |  |  |  |  |  |  |  |  |
| Midwest | 95 | 16.8 | 175 | 18.2 | 0.04 | 48 | 16.4 | 59 | 20.2 | 0.10 |
| South | 159 | 28.1 | 249 | 25.9 | 0.05 | 69 | 23.6 | 72 | 24.7 | 0.02 |
| Northeast | 166 | 29.3 | 264 | 27.5 | 0.04 | 84 | 28.8 | 87 | 29.8 | 0.02 |
| West | 146 | 25.8 | 273 | 28.4 | 0.06 | 91 | 31.2 | 74 | 25.3 | 0.13 |
| Duration of health plan enrollment prior to index date (days) | 1264 | 758.3 | 1322 | 721.5 | 0.08 | 1331 | 755.9 | 1406 | 751.2 | 0.10 |
| **Medical History** | | | | | | | | | | |
| Other primary cancer prior to first breast cancer diagnosis code | 223 | 39.4 | 365 | 38.0 | 0.03 | 125 | 42.8 | 123 | 42.1 | 0.01 |
| Secondary malignancy (metastasis) |  |  |  |  |  |  |  |  |  |  |
| Lymph nodes of head, face, and neck | 154 | 27.2 | 211 | 22.0 | 0.12 | 77 | 26.4 | 62 | 21.2 | 0.12 |
| Respiratory and digestive systems | 257 | 45.4 | 332 | 34.5 | 0.22 | 119 | 40.8 | 108 | 37.0 | 0.08 |
| Other specified sites | 463 | 81.8 | 662 | 68.9 | 0.30 | 216 | 74.0 | 217 | 74.3 | 0.01 |
| Deyo-Charlson comorbidity index (DCI) | 8.52 | 1.8 | 7.94 | 2.7 | 0.26 | 8.31 | 2.0 | 8.24 | 2.4 | 0.03 |
| Secondary malignant neoplasm of breast | 66 | 11.7 | 90 | 9.4 | 0.07 | 35 | 12.0 | 36 | 12.3 | 0.01 |
| Breast Cancer (Female) diagnosis code | 558 | 98.6 | 924 | 96.1 | 0.15 | 284 | 97.3 | 282 | 96.6 | 0.04 |
| InSitu Breast Cancer | 44 | 7.8 | 63 | 6.6 | 0.05 | 15 | 5.1 | 16 | 5.5 | 0.02 |
| History of Breast Cancer | 327 | 57.8 | 358 | 37.3 | 0.42 | 147 | 50.3 | 132 | 45.2 | 0.10 |
| **Cancer Therapy History** | | | | | | | | | | |
| **Radiation therapy** | 112 | 19.8 | 173 | 18.0 | 0.05 | 54 | 18.5 | 63 | 21.6 | 0.08 |
| Implantation (Brachytherapy) | 0 | 0.0 | ≤10 | n/a | n/a | 0 | 0.0 | 0 | 0.0 | n/a |
| External Beam | 112 | 19.8 | 173 | 18.0 | 0.05 | 54 | 18.5 | 63 | 21.6 | 0.08 |
| **Surgery** | 11 | 1.9 | 33 | 3.4 | 0.09 | ≤10 | n/a | 12 | 4.1 | 0.10 |
| Mastectomy in the last six months | ≤10 | n/a | 12 | 1.2 | 0.08 | ≤10 | n/a | ≤10 | n/a | 0.11 |
| Lumpectomy in the last six months | ≤10 | n/a | 17 | 1.8 | 0.12 | ≤10 | n/a | ≤10 | n/a | 0.12 |
| Radical Mastectomy in the last six months | ≤10 | n/a | ≤10 | n/a | 0.02 | ≤10 | n/a | ≤10 | n/a | 0.06 |
| **Chemotherapy** | 100 | 17.7 | 126 | 13.1 | 0.13 | 42 | 14.4 | 40 | 13.7 | 0.02 |
| Infusion based chemo (procedure) | ≤10 | n/a | ≤10 | n/a | 0.05 | ≤10 | n/a | ≤10 | n/a | 0.14 |
| **Imaging** |  |  |  |  |  |  |  |  |  |  |
| CT related imaging in the last six months | 139 | 24.6 | 178 | 18.5 | 0.15 | 62 | 21.2 | 64 | 21.9 | 0.02 |
| MR related imaging for needle placement | 0 | 0.0 | 0 | 0.0 | n/a | 0 | 0.0 | 0 | 0.0 | n/a |
| Diagnostic imaging in the last six months | 72 | 12.7 | 154 | 16.0 | 0.09 | 38 | 13.0 | 36 | 12.3 | 0.02 |
| Mammography | ≤10 | n/a | 21 | 2.2 | 0.06 | ≤10 | n/a | ≤10 | n/a | 0.05 |
| MRI related imaging | 25 | 4.4 | 47 | 4.9 | 0.02 | 13 | 4.5 | 13 | 4.5 | 0.00 |
| Tomosynthesis (3D Mammography) | 18 | 3.2 | 15 | 1.6 | 0.11 | 0 | 0.0 | 0 | 0.0 | n/a |
| **Healthcare Utilization (Six months prior to index date)** | | | | | | | | | | |
| Number of outpatient visits | 38.6 | 23.6 | 36.8 | 25.8 | 0.07 | 38.7 | 26.4 | 37.6 | 22.8 | 0.05 |
| Number of outpatient visits to an oncologist | 0.0 | 0.2 | 0.0 | 0.3 | 0.04 | 0.0 | 0.2 | 0.0 | 0.3 | 0.04 |
| Number of inpatient hospitalizations | 0.3 | 0.7 | 0.4 | 0.7 | 0.04 | 0.3 | 0.7 | 0.4 | 0.8 | 0.10 |
| Number of inpatient hospitalizations for breast cancer | 0.0 | 0.2 | 0.1 | 0.4 | 0.25 | 0.1 | 0.3 | 0.1 | 0.4 | 0.11 |
| Number of inpatient hospitalizations for any cancer | 0.3 | 0.6 | 0.3 | 0.7 | 0.03 | 0.3 | 0.6 | 0.4 | 0.7 | 0.12 |
| Number of emergency department visits | 0.3 | 0.8 | 0.3 | 0.7 | 0.03 | 0.3 | 0.8 | 0.3 | 0.6 | 0.03 |
| **Medication Use (breast cancer related)** | | | | | | | | | | |
| Palbociclib | 0 | 0.0 | 0 | 0.0 | n/a | 0 | 0.0 | 0 | 0.0 | n/a |
| Hormone Therapy | 326 | 57.6 | 482 | 50.2 | 0.15 | 160 | 54.8 | 158 | 54.1 | 0.0 |
| Letrozole | 116 | 20.5 | 147 | 15.3 | 0.14 | 55 | 18.8 | 51 | 17.5 | 0.0 |
| Anastrazole | 136 | 24.0 | 240 | 25.0 | 0.02 | 67 | 22.9 | 72 | 24.7 | 0.0 |
| Exemestane | 86 | 15.2 | 122 | 12.7 | 0.07 | 42 | 14.4 | 49 | 16.8 | 0.1 |
| HER2 positive Therapy | 15 | 2.7 | 96 | 10.0 | 0.31 | 14 | 4.8 | 16 | 5.5 | 0.0 |
| Trastuzumab | 25 | 4.4 | 131 | 13.6 | 0.33 | 17 | 5.8 | 29 | 9.9 | 0.2 |
| Lapatinib | ≤10 | n/a | ≤10 | n/a | 0.01 | ≤10 | n/a | ≤10 | n/a | 0.0 |
| Ado-trastuzumab | 0 | 0.0 | 0 | 0.0 | n/a | 0 | 0.0 | 0 | 0.0 | n/a |
| Pertuzumab | 0 | 0.0 | 0 | 0.0 | n/a | 0 | 0.0 | 0 | 0.0 | n/a |
| Tamoxifen | 140 | 24.7 | 189 | 19.7 | 0.1 | 66 | 22.6 | 61 | 20.9 | 0.0 |
| Fulvestrant | 0 | 0.0 | 0 | 0.0 | n/a | 0 | 0.0 | 0 | 0.0 | n/a |
| Denosumab or Pamidronate | 206 | 36.4 | 234 | 24.3 | 0.3 | 82 | 28.1 | 85 | 29.1 | 0.0 |
| Everolimus | 40 | 7.1 | 33 | 3.4 | 0.2 | 14 | 4.8 | 17 | 5.8 | 0.0 |
| **Medication Use (not breast cancer related)** | | | | | | | | | | |
| Anticonvulsants | 126 | 22.3 | 146 | 15.2 | 0.18 | 49 | 16.8 | 45 | 15.4 | 0.04 |
| Antidepressants | 178 | 31.4 | 252 | 26.2 | 0.12 | 86 | 29.5 | 83 | 28.4 | 0.02 |
| Antidiabetics | 67 | 11.8 | 112 | 11.7 | 0.01 | 26 | 8.9 | 34 | 11.6 | 0.09 |
| Antifungals | 28 | 4.9 | 42 | 4.4 | 0.03 | 17 | 5.8 | ≤10 | n/a | 0.11 |
| Antihypertensives | 173 | 30.6 | 252 | 26.2 | 0.10 | 80 | 27.4 | 105 | 36.0 | 0.18 |
| Antimycobacterials | 109 | 19.3 | 196 | 20.4 | 0.03 | 56 | 19.2 | 70 | 24.0 | 0.12 |
| Antivirals | 32 | 5.7 | 30 | 3.1 | 0.12 | 17 | 5.8 | 13 | 4.5 | 0.06 |
| Corticosteroids | 148 | 26.1 | 200 | 20.8 | 0.13 | 65 | 22.3 | 76 | 26.0 | 0.09 |
| Oral contraceptive use (progestin) | ≤10 | n/a | ≤10 | n/a | 0.02 | 0 | 0.0 | ≤10 | n/a | n/a |
| Oral contraceptive use (combination) | ≤10 | n/a | ≤10 | n/a | 0.01 | 0 | 0.0 | ≤10 | n/a | n/a |
| Oral contraceptive use (unspecified) | ≤10 | n/a | 11 | 1.1 | 0.01 | ≤10 | n/a | ≤10 | n/a | 0.14 |
| Lipid lowering agent | 132 | 23.3 | 212 | 22.1 | 0.03 | 67 | 22.9 | 66 | 22.6 | 0.01 |
| Vaginal estrogen (local hormone treatment) | 0 | 0.0 | 0 | 0.0 | n/a | 0 | 0.0 | 0 | 0.0 | n/a |
| Macrolides | ≤10 | n/a | 12 | 1.2 | 0.03 | ≤10 | n/a | ≤10 | n/a | 0.08 |
| Sedatives/hypnotics | 57 | 10.1 | 67 | 7.0 | 0.11 | 23 | 7.9 | 21 | 7.2 | 0.03 |
| Selective estrogen receptor modulators | ≤10 | n/a | ≤10 | n/a | 0.03 | 0 | 0.0 | 0 | 0.0 | n/a |
| Unopposed estrogen hormone replacement therapy (HRT) | 0 | 0.0 | ≤10 | n/a | n/a | 0 | 0.0 | ≤10 | n/a | n/a |
| **Co-morbidities (six months prior to index date)** | | | | | | | | | | |
| Pathologic fracture | 46 | 8.1 | 62 | 6.5 | 0.06 | 20 | 6.8 | 20 | 6.8 | 0.00 |
| Osteoporosis | 56 | 9.9 | 116 | 12.1 | 0.07 | 33 | 11.3 | 36 | 12.3 | 0.03 |
| Uterine malignancies | ≤10 | n/a | ≤10 | n/a | 0.07 | 0 | 0.0 | 0 | 0.0 | n/a |
| Pure hypercholesterolemia | 48 | 8.5 | 99 | 10.3 | 0.06 | 32 | 11.0 | 29 | 9.9 | 0.03 |
| Major adverse cardiac events (MACE) | 20 | 3.5 | 42 | 4.4 | 0.04 | 14 | 4.8 | 12 | 4.1 | 0.03 |
| Acute myocardial infarction (MI) | ≤10 | n/a | ≤10 | n/a | 0.04 | ≤10 | n/a | ≤10 | n/a | 0.04 |
| Cerebrovascular disease | 19 | 3.4 | 47 | 4.9 | 0.08 | 13 | 4.5 | 11 | 3.8 | 0.03 |
| Stroke | 16 | 2.8 | 34 | 3.5 | 0.04 | 12 | 4.1 | ≤10 | n/a | 0.06 |
| Diabetes | 67 | 11.8 | 112 | 11.7 | 0.01 | 26 | 8.9 | 34 | 11.6 | 0.09 |
| Hyperglycemia | 27 | 4.8 | 42 | 4.4 | 0.02 | 11 | 3.8 | ≤10 | n/a | 0.02 |
| Deyo-Charlson Index (DCI) |  |  |  |  |  |  |  |  |  |  |
| 0-3 | 19 | 3.4 | 123 | 12.8 | 0.35 | 15 | 5.1 | 30 | 10.3 | 0.19 |
| 4-7 | 12 | 2.1 | 45 | 4.7 | 0.14 | 12 | 4.1 | ≤10 | n/a | 0.06 |
| 8-11 | 512 | 90.5 | 739 | 76.9 | 0.37 | 252 | 86.3 | 238 | 81.5 | 0.13 |
| 12 or more | 23 | 4.1 | 54 | 5.6 | 0.07 | 13 | 4.5 | 15 | 5.1 | 0.03 |
| **ALI related risk factors** | | | | | | | | | | |
| Chronic liver disease or Alcoholism | 74 | 13.1 | 98 | 10.2 | 0.09 | 34 | 11.6 | 32 | 11.0 | 0.02 |
| Chronic or acute hepatitis | ≤10 | n/a | ≤10 | n/a | 0.02 | ≤10 | n/a | ≤10 | n/a | 0.04 |
| Chronic or acute disease of gallbladder or pancreas | 36 | 6.4 | 86 | 8.9 | 0.10 | 25 | 8.6 | 29 | 9.9 | 0.05 |
| Hepatic, Biliary or pancreatic cancer | 160 | 28.3 | 190 | 19.8 | 0.20 | 69 | 23.6 | 71 | 24.3 | 0.02 |
| Congestive heart failure | 29 | 5.1 | 64 | 6.7 | 0.07 | 17 | 5.8 | 20 | 6.8 | 0.04 |
| **Medications association with liver injury** | | | | | | | | | | |
| **Any medication (of list below)** | 467 | 82.5 | 689 | 71.7 | 0.26 | 220 | 75.3 | 226 | 77.4 | 0.05 |
| Acarbose | 0 | 0.0 | 0 | 0.0 | 0.00 | 0 | 0.0 | 0 | 0.0 | n/a |
| Acetaminophen (prescription) | 204 | 36.0 | 292 | 30.4 | 0.12 | 91 | 31.2 | 86 | 29.5 | 0.04 |
| Allopurinol | ≤10 | n/a | 13 | 1.4 | 0.03 | ≤10 | n/a | ≤10 | n/a | 0.03 |
| Amiodarone | ≤10 | n/a | ≤10 | n/a | 0.04 | ≤10 | n/a | ≤10 | n/a | 0.05 |
| Amitriptyline | ≤10 | n/a | ≤10 | n/a | 0.06 | ≤10 | n/a | ≤10 | n/a | 0.04 |
| Amoxicillin + clavulanic acid | 33 | 5.8 | 62 | 6.5 | 0.03 | 18 | 6.2 | 23 | 7.9 | 0.07 |
| Anabolic steroids | 0 | 0.0 | ≤10 | n/a | n/a | 0 | 0.0 | 0 | 0.0 | n/a |
| Aripiprazole | ≤10 | n/a | ≤10 | n/a | 0.07 | 0 | 0.0 | 0 | 0.0 | n/a |
| Azathioprine | ≤10 | n/a | 0 | 0.0 | n/a | 0 | 0.0 | 0 | 0.0 | n/a |
| Baclofen | ≤10 | n/a | ≤10 | n/a | 0.04 | ≤10 | n/a | ≤10 | n/a | 0.08 |
| Bupropion | 0 | 0.0 | 0 | 0.0 | n/a | 0 | 0.0 | 0 | 0.0 | n/a |
| Captopril | 0 | 0.0 | 0 | 0.0 | n/a | 0 | 0.0 | 0 | 0.0 | n/a |
| Carbamazepine | ≤10 | n/a | ≤10 | n/a | 0.08 | 0 | 0.0 | 0 | 0.0 | n/a |
| Chlorpromazine | ≤10 | n/a | ≤10 | n/a | 0.02 | 0 | 0.0 | 0 | 0.0 | n/a |
| Ciprofloxacin | 46 | 8.1 | 87 | 9.1 | 0.03 | 25 | 8.6 | 28 | 9.6 | 0.04 |
| Clindamycin | 23 | 4.1 | 33 | 3.4 | 0.03 | ≤10 | n/a | ≤10 | n/a | 0.04 |
| Clopidogrel | ≤10 | n/a | 21 | 2.2 | 0.04 | ≤10 | n/a | ≤10 | n/a | 0.00 |
| Cyproheptadine | ≤10 | n/a | ≤10 | n/a | 0.03 | ≤10 | n/a | ≤10 | n/a | 0.00 |
| Duloxetine | 23 | 4.1 | 32 | 3.3 | 0.04 | 14 | 4.8 | ≤10 | n/a | 0.09 |
| Enalapril | ≤10 | n/a | ≤10 | n/a | 0.02 | ≤10 | n/a | ≤10 | n/a | 0.08 |
| Erythromycins | 0 | 0.0 | ≤10 | n/a | n/a | 0 | 0.0 | 0 | 0.0 | n/a |
| Estrogens | 63 | 11.1 | 99 | 10.3 | 0.03 | 34 | 11.6 | 31 | 10.6 | 0.03 |
| Fluoxetine | 12 | 2.1 | 14 | 1.5 | 0.05 | ≤10 | n/a | ≤10 | n/a | 0.00 |
| Flutamide | 0 | 0.0 | 0 | 0.0 | n/a | 0 | 0.0 | 0 | 0.0 | n/a |
| HAART drugs | 0 | 0.0 | 0 | 0.0 | n/a | 0 | 0.0 | 0 | 0.0 | n/a |
| Irbesartan | ≤10 | n/a | ≤10 | n/a | 0.06 | 0 | 0.0 | 0 | 0.0 | n/a |
| Isoniazid | 0 | 0.0 | 0 | 0.0 | n/a | 0 | 0.0 | 0 | 0.0 | n/a |
| Ketoconazole | ≤10 | n/a | ≤10 | n/a | 0.01 | ≤10 | n/a | ≤10 | n/a | 0.04 |
| Lamotrigine | ≤10 | n/a | ≤10 | n/a | 0.04 | ≤10 | n/a | ≤10 | n/a | 0.00 |
| Lisinopril | 76 | 13.4 | 87 | 9.1 | 0.14 | 29 | 9.9 | 33 | 11.3 | 0.04 |
| Losartan | 54 | 9.5 | 72 | 7.5 | 0.07 | 23 | 7.9 | 35 | 12.0 | 0.14 |
| Methotrexate | ≤10 | n/a | ≤10 | n/a | 0.06 | ≤10 | n/a | ≤10 | n/a | 0.00 |
| Mirtazapine | ≤10 | n/a | ≤10 | n/a | 0.02 | ≤10 | n/a | ≤10 | n/a | 0.06 |
| Nitrofurantoin | 10 | 1.8 | 31 | 3.2 | 0.09 | ≤10 | n/a | ≤10 | n/a | 0.02 |
| NSAIDs | 129 | 22.8 | 161 | 16.8 | 0.15 | 41 | 14.0 | 58 | 19.9 | 0.16 |
| Omeprazole | 76 | 13.4 | 125 | 13.0 | 0.01 | 34 | 11.6 | 35 | 12.0 | 0.01 |
| Oral contraceptives | ≤10 | n/a | ≤10 | n/a | 0.01 | 0 | 0.0 | ≤10 | n/a | n/a |
| Paroxetine | ≤10 | n/a | 22 | 2.3 | 0.05 | ≤10 | n/a | ≤10 | n/a | 0.04 |
| Phenobarbital | 0 | 0.0 | 0 | 0.0 | n/a | 0 | 0.0 | 0 | 0.0 | n/a |
| Phenothiazines | 92 | 16.3 | 90 | 9.4 | 0.21 | 25 | 8.6 | 34 | 11.6 | 0.10 |
| Phenytoin | 0 | 0.0 | ≤10 | n/a | n/a | 0 | 0.0 | 0 | 0.0 | n/a |
| Pyrazinamide | 0 | 0.0 | 0 | 0.0 | n/a | 0 | 0.0 | 0 | 0.0 | n/a |
| Rifampicin | 0 | 0.0 | ≤10 | n/a | n/a | 0 | 0.0 | 0 | 0.0 | n/a |
| Risperidone | ≤10 | n/a | ≤10 | n/a | 0.08 | 0 | 0.0 | 0 | 0.0 | n/a |
| Sertraline | 19 | 3.4 | 34 | 3.5 | 0.01 | ≤10 | n/a | 16 | 5.5 | 0.10 |
| Statins | 125 | 22.1 | 197 | 20.5 | 0.04 | 65 | 22.3 | 61 | 20.9 | 0.03 |
| Sulfonamides | 0 | 0.0 | ≤10 | n/a | n/a | 0 | 0.0 | ≤10 | n/a | n/a |
| Terbinafine | ≤10 | n/a | ≤10 | n/a | 0.02 | 0 | 0.0 | 0 | 0.0 | n/a |
| Tetracyclines | 31 | 5.5 | 44 | 4.6 | 0.04 | 14 | 4.8 | 16 | 5.5 | 0.03 |
| Trazodone | 15 | 2.7 | 24 | 2.5 | 0.01 | 11 | 3.8 | 13 | 4.5 | 0.03 |
| Tricyclics | 0 | 0.0 | 0 | 0.0 | n/a | 0 | 0.0 | 0 | 0.0 | n/a |
| Trimethoprim-sulfamethoxazole | 28 | 4.9 | 58 | 6.0 | 0.05 | 13 | 4.5 | 19 | 6.5 | 0.09 |
| Trovafloxacin | 0 | 0.0 | 0 | 0.0 | n/a | 0 | 0.0 | 0 | 0.0 | n/a |
| Valproic acid | ≤10 | n/a | ≤10 | n/a | 0.03 | 0 | 0.0 | 0 | 0.0 | n/a |
| Verapamil | ≤10 | n/a | 11 | 1.1 | 0.07 | ≤10 | n/a | ≤10 | n/a | 0.05 |
| Abbreviations: Feb., February; ER, estrogen receptor; HER2, human epidermal growth factor receptor 2; N, number; STD, standard deviation; CT, computed tomography; MRI, magnetic resonance imaging; ALI, acute liver injury; HAART, highly active antiretroviral therapy; NSAIDs, nonsteroidal anti-inflammatory drugs. | | | | | | | | | | |
| *All characteristics are measured as presence within six months prior to the index date, unless otherwise specified. | | | | | | | | | | |
| ^The following variables were included in the propensity score: age, calendar year of index date, region, Deyo-Charlson Index, number of outpatient visits, number of emergency room visits, secondary malignancy to lymph nodes of head, face, and neck, secondary malignancy to other specified sites, secondary malignancy to respiratory sites, tamoxifen, everolimus, anastrazole, denosumab or pamidronate, exemestane, chemotherapy, corticosteroids, diagnostic imaging, breast cancer surgery, letrozole, HER2 positive therapy, radiation therapy, CT imaging, mammography, MRI imaging, anticonvulsants, antidepressants, sedatives/hypnotics, secondary malignancy to breast, breast cancer diagnosis code, in situ breast cancer diagnosis, hyperglycemia, cerebrovascular disease, Chronic liver disease or Alcoholism, Chronic or acute disease of gallbladder or pancreas, Hepatic, Biliary or pancreatic cancer, Congestive heart failure, any medication associated with ALI- Acetaminophen, Allopurinol, Amiodarone, Amitriptyline, + clavulanic acid, Aripiprazole, Baclofen, Ciprofloxacin, Clindamycin, Clopidogrel, Duloxetine, Estrogens, Fluoxetine, Ketoconazole, Lisinopril, Losartan, Mirtazapine, Nitrofurantoin, NSAIDs, Omeprazole, Paroxetine, Phenothiazine, Sertraline, Statins, Tetracycline, Trazodone, and Trimethoprim. | | | | | | | | | | |

# **Supplemental Table 9:** Unadjusted and Adjusted Hazard Ratios of ALI in New Users of Palbociclib and Fulvestrant and New Users of Fulvestrant Monotherapy (Contemporaneous Comparator)

|  | **Unadjusted Hazard Ratios** | | | | | **Adjusted Hazard Ratios^*^** | | | | | |
| --- | --- | --- | --- | --- | --- | --- | --- | --- | --- | --- | --- |
| **Event** | **Palbociclib-fulvestrant** | **Fulvestrant monotherapy** | **HR** | **95% LCL** | **95% UCL** | **Palbociclib-fulvestrant** | | **Fulvestrant monotherapy** | **aHR** | **95% LCL** | **95% UCL** |
|  | **IR per 100 person-years** | |  |  |  | **IR per 100 person-years** | | |  |  |  |
| Acute liver injury (primary algorithm*) | 4.0 | 2.0 | 2.1 | 0.9 | 4.7 | 1.6 | 3.6 | | 0.5 | 0.1 | 2.2 |
| Acute liver injury - 2 (original with labs algorithm*) | 4.0 | 2.9 | 1.5 | 0.7 | 3.0 | 2.7 | 4.3 | | 0.7 | 0.2 | 2.4 |
| Acute liver injury - 3 (sensitive algorithm*) | 12.3 | 5.3 | 2.3 | 1.4 | 3.8 | 9.8 | 8.7 | | 1.2 | 0.6 | 2.5 |
| Acute liver injury - 4 (specific algorithm*) | 0 | 0 | -- | -- | -- | 0 | 0 | | -- | -- | -- |
| Abbreviations: LCL, lower confidence limit; UCL, confidence limit; IR, incidence rate; HR, hazard ratio; aHR, adjusted hazard ratio. | | | | | | | | | | | |
| *Algorithm definitions provided in Supplemental Table 1 | | | | | | | | | | | |
| ^The propensity score included the following variables: age, region, DCI, number of outpatient visits, number of emergency room visits, secondary malignancy to lymph nodes of head, face, and neck, secondary malignancy to other specified sites, secondary malignancy to respiratory sites, tamoxifen, everolimus, anastrazole, denosumab or pamidronate, exemestane, chemotherapy, corticosteroids, diagnostic imaging, breast cancer surgery, letrozole, HER2+ therapy, radiation therapy, CT imaging, mammography, MRI imaging, anticonvulsants, antidepressants, sedatives/hypnotics, secondary malignancy to breast, breast cancer diagnosis code, in situ breast cancer diagnosis, hyperglycemia, cerebrovascular disease, chronic liver disease or Alcoholism, chronic or acute disease of gallbladder or pancreas, hepatic, biliary or pancreatic cancer, congestive heart failure, any medication associated with ALI, including acetaminophen, allopurinol, amiodarone, amitriptyline, clavulanic acid, aripiprazole, baclofen, ciprofloxacin, clindamycin, clopidogrel, duloxetine, estrogens, fluoxetine, ketoconazole, lisinopril, losartan, mirtazapine, nitrofurantoin, NSAIDs, omeprazole, paroxetine, phenothiazine, sertraline, statins, tetracycline, trazodone, and trimethoprim. | | | | | | | | | | | |

**Supplemental Table 10:** Incidence of ALI in the HealthCore Integrated Database (HIRD) Between April 2014 and March 2017

| **Event** | **April 2014 to December 2014** | | | **January 2015 to September 2015** | | | **October 2015 to June 2016** | | | **July 2016 to March 2017** | | |
| --- | --- | --- | --- | --- | --- | --- | --- | --- | --- | --- | --- | --- |
|  | **IR (per 100 person-years)** | | | **IR (per 100 person-years)** | | | **IR (per 100 person-years)** | | | **IR (per 100 person-years)** | | |
|  | **IR** | **95% Lower CI** | **95% Upper CI** | **IR** | **95% Lower CI** | **95% Upper CI** | **IR** | **95% Lower CI** | **95% Upper CI** | **IR** | **95% Lower CI** | **95% Upper CI** |
| **Overall - All HIRD*** | | | | | | | | | | | | |
| ALI (primary algorithm) | 0.31 | 0.31 | 0.32 | 0.34 | 0.33 | 0.35 | 0.36 | 0.35 | 0.37 | 0.37 | 0.36 | 0.38 |
| Abbreviations: ALI, acute liver injury; IR, incidence rate; CI, confidence interval; HIRD, HealthCore Integrated Research Database. | | | | | | | | | | | | |
| *Included all individuals in the HIRD, regardless of whether they were dispensed palbociclib or fulvestrant | | | | | | | | | | | | |

# **Supplemental Table 11:** ALI Algorithm Signal Refinement – Validation of Claims Algorithms Compared to Medical Record Adjudication

| **Code or combination of codes** | **Number of provisional cases with collected medical record** | | **Number of confirmed cases and non-cases** | | **Number of confirmed cases** | | **PPV (95% CI)** | **95% CI** | |
| --- | --- | --- | --- | --- | --- | --- | --- | --- | --- |
| **ALI - All adjudication results*** | | | | | | | | | |
| Algorithm 1 (primary algorithm) | | 29 | | 25 | | 21 | 0.84 | 0.64 | 0.95 |
| Algorithm 2 (original with labs) | | 29 | | 25 | | 18 | 0.72 | 0.51 | 0.88 |
| Algorithm 3 (sensitive) | | 52 | | 40 | | 29 | 0.73 | 0.56 | 0.85 |
| Abbreviations: ALI, acute liver injury; PPV, positive predictive value; CI, confidence interval. | | | | | | | | | |
| *Algorithm definitions provided in Supplemental Table 1. ALI algorithm 4 (specific) did not have sufficient number of cases to report, as there are privacy restrictions in providing results in counts ≤10. | | | | | | | | | |

**Supplemental Table 12:** PPV Adjusted Hazard Ratios of ALI in New Users of Palbociclib and Fulvestrant and New Users of Fulvestrant Monotherapy (Historical Comparator)

|  | **Adjusted Hazard Ratios^^^**^&^ | | | | |
| --- | --- | --- | --- | --- | --- |
| **Event** | **Palbociclib-fulvestrant** | **Fulvestrant monotherapy** | **aHR** | **95% LCL** | **95% UCL** |
|  | **IR per 100 person-years** | |  |  |  |
| Acute liver injury (primary algorithm*) | 3.0 | 1.2 | 4.0 | 1.4 | 11.2 |
| Acute liver injury - 2 (original with labs algorithm*) | 3.7 | 0.6 | 0.7 | 0.3 | 1.8 |
| Acute liver injury - 3 (sensitive algorithm*) | 8.6 | 0.8 | 2.1 | 1.1 | 4.3 |
| Acute liver injury - 4 (specific algorithm*) | 0 | 0 | -- | -- | -- |
| Abbreviations: PT, person time; IR, incidence rate; CI, confidence interval; aHR, adjusted hazards ratio; LCL, lower confidence limit; UCL, upper confidence limit; ALI, acute liver injury; PPV, positive predictive value | | | | | |
| ^This propensity score matched population was further adjusted using PPVs from the validation study, assuming 100% sensitivity for each algorithm. | | | | | |
| ^&^The following variables were included in the propensity score: age, region, Deyo-Charlson Index, number of outpatient visits, number of emergency room visits, secondary malignancy to lymph nodes of head, face, and neck, secondary malignancy to other specified sites, secondary malignancy to respiratory sites, tamoxifen, everolimus, anastrazole, denosumab or pamidronate, exemestane, chemotherapy, corticosteroids, diagnostic imaging, breast cancer surgery, letrozole, HER2 positive therapy, radiation therapy, CT imaging, mammography, MRI imaging, anticonvulsants, antidepressants, sedatives/hypnotics, secondary malignancy to breast, breast cancer diagnosis code, in situ breast cancer diagnosis, hyperglycemia, cerebrovascular disease, Chronic liver disease or Alcoholism, Chronic or acute disease of gallbladder or pancreas, Hepatic, Biliary or pancreatic cancer, Congestive heart failure, any medication associated with ALI- Acetaminophen, Allopurinol, Amiodarone, Amitriptyline, + clavulanic acid, Aripiprazole, Baclofen, Ciprofloxacin, Clindamycin, Clopidogrel, Duloxetine, Estrogens, Fluoxetine, Ketoconazole, Lisinopril, Losartan, Mirtazapine, Nitrofurantoin, NSAIDs, Omeprazole, Paroxetine, Phenothiazine, Sertraline, Statins, Tetracycline, Trazodone, and Trimethoprim. | | | | | |

# **Supplemental Figure 1:** E-value to Explain the Association Between Palbociclib-Fulvestrant and the Primary ALI algorithm (in Historical Fulvestrant Analyses)


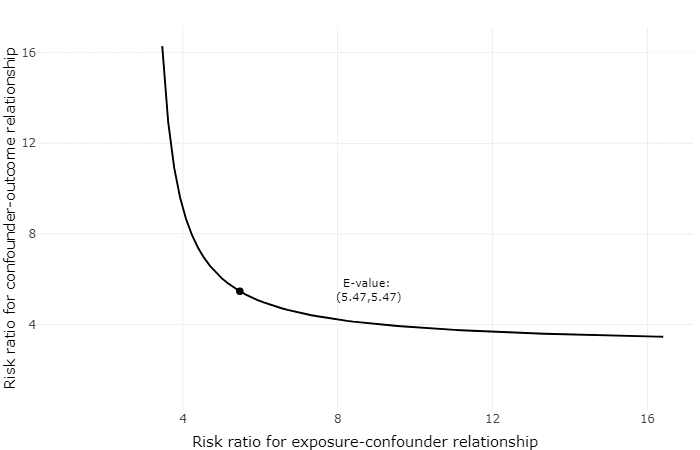


*Minimum strength required for both the palbociclib-confounder and confounder-acute liver injury (ALI) relationships to explain away the estimated relationship between palbociclib and ALI. The E-value for the 95% confidence interval (CI) to remain significant is 1.34.
